# Image segmentation of treated and untreated tumor spheroids by fully convolutional networks

**DOI:** 10.1093/gigascience/giaf027

**Published:** 2025-05-07

**Authors:** Matthias Streller, Soňa Michlíková, Willy Ciecior, Katharina Lönnecke, Leoni A Kunz-Schughart, Steffen Lange, Anja Voss-Böhme

**Affiliations:** DataMedAssist Group, HTW Dresden–University of Applied Sciences, 01069 Dresden, Germany; Faculty of Informatics/Mathematics, HTW Dresden–University of Applied Sciences, 01069 Dresden, Germany; Helmholtz-Zentrum Dresden-Rossendorf, Institute of Radiooncology–OncoRay, 01328 Dresden, Germany; OncoRay–National Center for Radiation Research in Oncology, Faculty of Medicine and University Hospital Carl Gustav Carus, TUD Dresden University of Technology, Helmholtz-Zentrum Dresden-Rossendorf, 01307 Dresden, Germany; DataMedAssist Group, HTW Dresden–University of Applied Sciences, 01069 Dresden, Germany; Faculty of Informatics/Mathematics, HTW Dresden–University of Applied Sciences, 01069 Dresden, Germany; DataMedAssist Group, HTW Dresden–University of Applied Sciences, 01069 Dresden, Germany; Faculty of Informatics/Mathematics, HTW Dresden–University of Applied Sciences, 01069 Dresden, Germany; OncoRay–National Center for Radiation Research in Oncology, Faculty of Medicine and University Hospital Carl Gustav Carus, TUD Dresden University of Technology, Helmholtz-Zentrum Dresden-Rossendorf, 01307 Dresden, Germany; National Center for Tumor Diseases (NCT), NCT/UCC Dresden, 69192 Heidelberg, Germany; DataMedAssist Group, HTW Dresden–University of Applied Sciences, 01069 Dresden, Germany; OncoRay–National Center for Radiation Research in Oncology, Faculty of Medicine and University Hospital Carl Gustav Carus, TUD Dresden University of Technology, Helmholtz-Zentrum Dresden-Rossendorf, 01307 Dresden, Germany; DataMedAssist Group, HTW Dresden–University of Applied Sciences, 01069 Dresden, Germany; Faculty of Informatics/Mathematics, HTW Dresden–University of Applied Sciences, 01069 Dresden, Germany

**Keywords:** spheroids, organoids, brightfield microscopy, segmentation, deep learning, fully convolutional networks, high-content screening, 3D cancer models, cancer therapy, interobserver variability

## Abstract

**Background:**

Multicellular tumor spheroids (MCTS) are advanced cell culture systems for assessing the impact of combinatorial radio(chemo)therapy as they exhibit therapeutically relevant *in vivo–*like characteristics from 3-dimensional cell–cell and cell–matrix interactions to radial pathophysiological gradients. State-of-the-art assays quantify long-term curative endpoints based on collected brightfield image time series from large treated spheroid populations per irradiation dose and treatment arm. This analyses require laborious spheroid segmentation of up to 100,000 images per treatment arm to extract relevant structural information from the images (e.g., diameter, area, volume, and circularity). While several image analysis algorithms are available for spheroid segmentation, they all focus on compact MCTS with a clearly distinguishable outer rim throughout growth. However, they often fail for the common case of treated MCTS, which may partly be detached and destroyed and are usually obscured by dead cell debris.

**Results:**

To address these issues, we successfully train 2 fully convolutional networks, UNet and HRNet, and optimize their hyperparameters to develop an automatic segmentation for both untreated and treated MCTS. We extensively test the automatic segmentation on larger, independent datasets and observe high accuracy for most images with Jaccard indices around 90%. For cases with lower accuracy, we demonstrate that the deviation is comparable to the interobserver variability. We also test against previously published datasets and spheroid segmentations.

**Conclusions:**

The developed automatic segmentation can not only be used directly but also integrated into existing spheroid analysis pipelines and tools. This facilitates the analysis of 3-dimensional spheroid assay experiments and contributes to the reproducibility and standardization of this preclinical *in vitro* model.

**Key Points:**

- Three-dimensional tumor spheroids as an advocated preclinical model lack reliable image segmentation.
- An automatic spheroid segmentation even for treated spheroids obscured by dead cell debris is developed.
- Segmentation is tested against independent datasets and compared to previous deep learning models.
- High-quality training dataset with challenging cases of treated spheroids is provided.
- Complementary software tool is released for high-throughput image analysis of spheroids.

## Introduction

One of the most challenging problems in oncology is the development of therapeutic approaches for tumor growth suppression and eradication, as well as designing and optimizing treatment protocols. In this context, 3-dimensional (3D) multicellular tumor spheroids (MCTS) are an advocated preclinical, *in vitro* model to systematically study possible means of tumor suppression, assess the curative effect of combinatorial radio(chemo)therapy, and predict the response of *in vivo* tumors [1–8]. In contrast to 2-dimensional (2D) clonogenic survival assays, which are known to reflect the therapeutic response of cancer cells in the tissue comparatively poorly [9], multicellular spheroids are reproducible 3D avascular clusters of several thousand tumor cells without or in advanced settings with stromal cell compartments mimicking the pathophysiological milieu of tumor microareas or micrometastases. Due to their more or less radial 3D histomorphology and structure, they exhibit many characteristic features affecting tumor growth dynamics, including 3D reciprocal cell–cell and cell–extracellular matrix interactions as well as metabolic gradients of oxygen, nutrients, and waste products, which strongly impair the cells’ proliferative activity and therapy response [4–8], in particular oxygen deficiency (hypoxia), which is associated with substantial radioresistance [10–13]. After a long phase in which spheroids were used only in specialized laboratories, methodological advances in serial culturing and live imaging have led to an exponential spread of this *in vitro* model system over the past 2 decades [7, 8, 14].

While 3D tumor spheroids provide a physiologically more realistic *in vitro* framework to study tumor growth dynamics and therapeutic outcomes, the analysis of the experiments is much more complex than for traditional 2D cultures. Three-dimensional tumor spheroids’ dynamics with and without treatment are most frequently monitored via microscopy imaging. State-of-the-art long-term spheroid-based assays assess therapy response from these image time series by classifying each spheroid within a population as either controlled or relapsed based on their growth kinetics. By averaging the therapeutic response over ensembles of spheroids for each treatment dose, the spheroid control probabilities and spheroid control doses are computed as analytical endpoints [15–18], analogous to the tumor control dose employed in *in vivo* radiotherapy experiments with tumor-bearing mice [19, 20]. Moreover, time points of relapse are identified to quantify growth recovery in terms of Kaplan–Meier curves. These metrics depend on the spheroid type (cell line), the size of the spheroids at the onset of treatment, and the applied treatment and often require the extraction of growth curves (i.e., spheroid volume over time) from the image time series of all individual spheroids.

The associated data analysis poses a significant, interdisciplinary challenge: a single, typical experimental series of a long-term spheroid-based assay, that is, 2 to 3 cell models at 10 different doses with 3 to 5 agents and 30 spheroids per treatment arm monitored up to 60 days after treatment generate up to $10^5$ images. In the past, several tools were developed for spheroid image analysis [21–26], including SpheroidSizer [27], AnaSP [28, 29], TASI [30], SpheroidJ [31], and INSIDIA [25, 32]. The most crucial part of spheroid image analysis is the identification of the set of pixels in an image that corresponds to the spheroid, as this forms the basis for the extraction of spheroid characteristics like diameter, volume, and circularity, as well as other morphologic features. This identification or classification of pixels in a given image is denoted semantic image segmentation [33] and represents the greatest challenge in spheroid image analysis. Previous spheroid segmentations were based on classical segmentation techniques, including thresholding methods (Yen [34], Otsu [28, 35]), watershed algorithm [36], shape-based detections (Hough transform algorithms [37], active contour models/snake  [38]), or edge detection (Canny, Sobel [31]), and are also available as ImageJ plugins [22, 25], MATLAB packages [27, 28, 30], or dedicated segmentation programs [39–41]. Since the characteristics of spheroid images, including the size, shape, and texture of the actual spheroid, vary with cell line, treatment, and microscopy method (e.g., brightfield [25, 29, 31, 32, 42], fluorescence [25, 26, 31, 32, 43, 44], differential, interference contrast [45]), most of these approaches and tools are specialized for specific experimental conditions or even selected microscopes [43, 44] and often fail to generalize to arbitrary conditions. In recent years, data-based methods, especially deep learning models, have been increasingly employed for spheroid segmentation to tackle this issue of generalization [29, 31, 42, 45–49]. Deep learning segmentation also allows more complex analysis (e.g., identification of time-lapse migratory patterns [45] or distinction of the spheroids’ core and invasive edge [32]).

**Figure 1:**
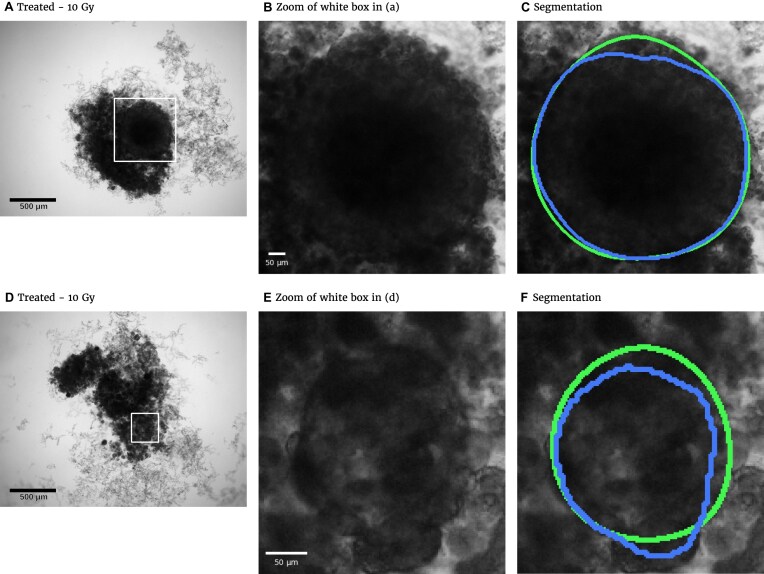
Representative examples of images for automatic segmentation with the optimized U-Net (blue) compared to the manual segmentation (green) for 3D tumor spheroids after treatment. The overlap with the manual segmentation is excellent for standard size and larger spheroids obscured by cell debris (top row) and sufficient for small, heavily obscured spheroids (bottom row). Displayed are (A, D) the original images, which are also the input for the U-Net; (B, D) magnified image details around the spheroids, as indicated by the white box in (A, D) for visibility; and (C, E) corresponding contours from the segmentations. The metrics for evaluation of the automatic segmentation are (top row) Jaccard distance (JCD) $= 7.6\%$, relative diameter deviation (RDD) $= 3.7\%$, and relative circularity deviation (RCD) $= 1.6\%$; bottom row: $\text{JCD} = 22.7\%$, $\text{RDD}= 7.5\%$, and $\text{RCD}= 14.7\%$; see text for details.

However, these segmentation and analysis tools have primarily been developed for experimental conditions where the resulting images are relatively clean with well-visible, unobscured MCTS. In contrast, radiotherapy, one of the most common cancer treatments, regularly causes spheroids to shed dead cells or even to detach completely. Consequently, dead cell debris often obscures the remaining shrunk MCTS and the 3D cultures putatively regrowing from surviving viable cells after detachment (see Fig. 1 for 2 examples). This debris can cover a much larger domain than the actual spheroid and can be locally even thicker and thus darker, which makes segmentation difficult even for human experts. Both classical segmentation techniques and previous deep learning models typically fail in these cases and require manual adjustments. We illustrate this challenge of spheroid segmentation by applying the 4 most recently proposed deep learning models [29, 31, 48] to images with and without severe cell debris (see Supplementary Fig. S1 for representative examples and Supplementary Table S1 for statistical results).

We train deep learning models to segment spheroid images after radiotherapy based on annotated images from a previous experiment assessing the radioresponse of human head and neck squamous cell carcinoma (HNSCC). After systematic optimization of hyperparameters and preprocessing for 2 selected network architectures, U-Net and HRNet, the automatic segmentation exhibits high accuracy for images of both treated and untreated spheroids. We further validate the automatic segmentation with the optimized U-Net on larger, independent datasets of 2 cell types of head and neck cancer exposed to both radiotherapy and hyperthermia. For most images, we find excellent overlap between manual and automatic segmentation, even in the case of small, heavily obscured spheroids (see Fig. 1). In particular, we compare segmentations of different biological experts and find that imprecisions of the automatic segmentation are comparable to variations across different humans. The optimized automatic segmentation is provided in a minimal tool with a graphical user interface.

## Materials

The original data are compiled from several datasets of time images of FaDu cell line spheroids, 1 of the 2 head and neck cancer cell types from the previously published study on combinatorial radioresponse in human HNSCC spheroid models [16]: spheroid populations were irradiated with X-rays at single doses of 2.5 Gy, 5.0 Gy, 7.5 Gy, or 10.0 Gy, with each dose being represented approximately equally in the dataset. Spheroids were imaged for up to 60 days after treatment with brightfield microscopy (i.e., at different times after treatment and with potentially different lighting conditions). In total, 1,095 spheroids were manually segmented by a biological expert (human H1) to train and test the fully convolutional networks (FCNs). We use the train-validation-test-split technique for validation during training, which is appropriate for the large datasets and commonly used to train spheroid segmentation models [29, 31, 48]. We split the annotated data into 3 separate datasets: 883 images for training, 108 for validation, and 104 for testing (hold-out test set). Images from a single spheroid are exclusively assigned to one of the groups to rule out unwanted correlations and data leakage between training, validation, and testing datasets. For further testing, another set of 6,574 images of head and neck cancer (FaDu and SAS) spheroids from the same study [16], treated with different combinations and doses of X-ray irradiation and hyperthermia, is manually segmented by several independent biological experts (humans H2–H5). Note that while the automatic segmentation is performed on individual images, biological experts always take precedent and subsequent images of a spheroid into account for manual segmentation.

The original images were taken with a Zeiss Axiovert 200M at a resolution of 1,300 × 1,030 pixels, representing $2.04\ \mu$m/pixel with 16-bit gray-scale per pixel [16]. Images are converted to 8-bit images to ensure compatibility with the employed libraries FastAi [50] and SemTorch [51]. While this conversion, in principle, reduces the contrast level, the effect is negligible as only a small fraction of the 16-bit range is utilized during imaging (e.g., the mean gray-scale value in the images is $1300\pm 130$, and values are rescaled such that the smallest gray-scale value of the original image becomes 0 and the biggest value becomes 255).

As deep learning models, we use FCNs, a particular group of convolutional neural networks intended for semantic image segmentation [52], in particular the U-Net [53] and the HRNet [54]. The U-Net is one of the most established FCN frameworks. The HRNet has a special architecture compared to other typical FCN models and has already been successfully employed in the case of clean images with clearly visible spheroids [31]. The pipeline to train the models employs FastAi [50] for the U-Net and its backbones and SemTorch [51] for the HRNet. Before each training, a suitable learning rate is picked using LR Range [55]. The training follows the 1-cycle policy: the learning rate is cycled once between 2 bounds, allowing fast convergence [55]. During training, we use validation-based early stopping, where the Jaccard distance (JCD) is the underlying metric. The models are trained on a GPU NVIDIA GeForce RTX 3080 with a mini-batch size of 2. For training on images with higher resolution or to compare some specific backbones, online learning is used instead of mini-batches to avoid memory overflow (i.e., the batch size, the amount of data used in each sub-epoch weight change, is reduced to 1).

## Methods

The segmentation is evaluated via several metrics (evaluation metrics: JCD, relative diameter deviation [RDD], relative circularity deviation [RCD], ambiguous spheroid fraction [ASF], invalid spheroid fraction [ISF]) additional to the standard accuracy quantified by the Jaccard coefficient of the whole dataset, which is used during training. These metrics are computed for individual images to report not only their mean values but also their deviations, and thus reliability, across the test dataset. These metrics are used to optimize the hyperparameters (“Hyperparameter optimization” section) and data augmentation (“Data augmentation” section) of the selected FCN models U-Net and HRNet for maximal accuracy.

### Evaluation metrics

**Figure 2:**
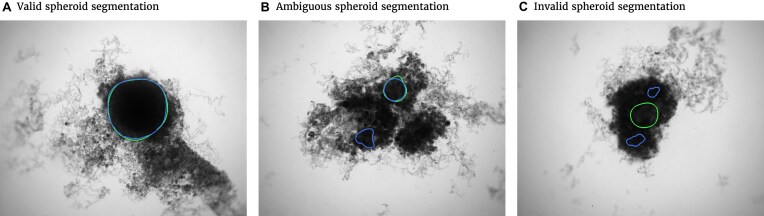
Exemplary evaluation of the automatic segmentation (blue) with respect to the manual one (green) for (A) standard case and (B, C) rare artifacts; see text for details. (A) Correctly segmented spheroid: no contribution to invalid spheroid fraction (ISF) or ambiguous spheroid fraction (ASF); JCD, RDD, and RCD are well defined. (B) Excessive spheroids are detected beyond the actual spheroid: no contribution to ISF, one count added to ASF; JCD, RDD, and RCD are well defined and computed for the larger, upper spheroid. (C) No overlap between automatic and manual segmentation: 1 count added to ISF, no contribution to ASF; JCD, RDD, and RCD are set to 1.

Several metrics are employed to asses the accuracy of the segmentation. The most important one is the JCD, which measures the relative difference between 2 sets of pixels *P* and *T*,


(1) \begin{eqnarray*}
\text{JCD} = 1 - \text{IoU} = 1 - \frac{|P \cap T|}{|P \cup T|}.
\end{eqnarray*}

with the automatically segmented (predicted) pixel set *P* from the FCN and the (target) pixel set *T* of the manually segmented spheroid. Note that in the literature, often the opposite metric, intersection over union $\text{IoU} = 1 - \text{JCD}$, also called Jaccard index, or Jaccard similarity coefficient, is used. The JCD takes values between 0 (automatic segmentation perfectly overlaps with the manual one) and 1 (no intersection between the 2 segmentations). The JCD can be understood as the relative area error of the segmentation; that is, in the example of Fig. 2A, $\text{JCD} = 0.042$ is obtained, which means $4.2\%$ error in the segmented area. A selection of sample images with different JCDs is displayed in Supplementary Figs. S2–S4 to give an optical reference for this metric. From these sample images and in accordance with the biological experts, it may be concluded that a JCD below 0.2 is justifiable, and such a deviation is observed between segmentations of different humans; see also “Further independent validation” section.

It is possible that the spheroid is correctly segmented, but additionally, another nonexistent spheroid is detected (see Fig. 2B, where additionally to the manually segmented spheroid in the top right, another contour in the bottom left is suggested by the automatic segmentation). In this case, the JCD would be below 1 but differ from 0. The fraction of these cases among all images is additionally denoted ASF. Furthermore, a JCD of 1 can mean that the spheroid is found in the wrong place or the spheroid is not found at all (see Fig. 2C). The fraction of these cases is denoted ISF.

In practice, only the average diameter $d_T = 2 \sqrt{|T|/\pi }$ of the segmented spheroid is extracted to estimate the 3D volume $\pi d_T^3/6$ of the spheroid under the assumption of a spherical shape. Additionally, the circularity $4 \pi |T|/L^2$ of the spheroid is computed from its area $|T|$ and perimeter *L* to assess the validity of this assumption. Note that this is just a common assumption in the field, although even a circular projection does not imply a spherical shape. Thus, we also assess the automatic segmentation by the resulting error of these 2 values and measure the RDD and RCD between the segmentations


(2) \begin{eqnarray*}
\text{RDD}, \text{RCD} = \frac{|c_P-c_T|}{c_T}
\end{eqnarray*}

where $c_P$ is either mean diameter or circularity based on the automatic segmentation and $c_T$ is the corresponding characteristic calculated based on the manual segmentation. In the rare case that several spheroids are detected in an image, the largest one is chosen for computation of the JCD. This strategy is in accordance with experimental standard practice for single spheroid-based assays with curative analytical endpoints, as the largest regrowing spheroid is sufficient to detect relapse after treatment. Note that if there were several spheroids present and of interest, also all of them could be segmented. In the case of $\text{JCD} = 1$, which corresponds to an invalid spheroid, RDD and RCD are also set to 1. The combination of the JCD, ASF, ISF, RDD, and RCD quantifies the accuracy of the segmentation with the FCN, where always a value closer to zero means higher agreement between the segmentations.

**Figure 3:**
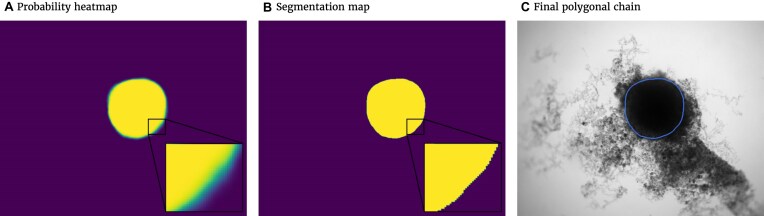
Postprocessing steps for the output of the FCN model to transform the probability heatmap to the spheroid contour. (A) Probability heatmap as output of the FCN model. Each pixel takes probability values between 0 (black) and 1 (white) predicting the target (spheroid). Note that due to the steep gradient, the gradual change from black to white is hard to see. (B) By using a threshold of 0.5, the pixels are classified into outside spheroid (black) and inside spheroid (white). (C) Contour of the spheroid border extracted as a polygonal chain (blue line displayed on original image).

The spheroid’s pixel set and perimeter are required to calculate the metrics. Thus, probability heatmaps, created by the Softmax function in the last layer of the FCN, are transformed into binary images and polygonal chains, as illustrated in Fig. 3. First, the binary image is created. For this purpose, every pixel with a probability value higher than or equal to 50% will be counted as a spheroid pixel and set to 1. Values of the other pixels are set to 0. The coordinates from the outer border of the emerged shape in the binary image can be extracted by the algorithm proposed in [56], thereby creating the final polygonal chain.

### Hyperparameter optimization

Several hyperparameters and preprocessing methods can be adjusted to improve the accuracy of the selected FCN models. We successively optimize the most relevant parameters, starting from the ones with the most significant expected impact on accuracy to the one with the lowest impact (i.e., *backbone, transfer learning, data augmentation, input resolution, loss function*, and *optimizer*). The *backbone* defines the exact architecture of the FCN model and is accordingly the most important hyperparameter. For *transfer learning*, the ImageNet [57] dataset is utilized. The *data augmentation* techniques are described in the “Data augmentation” section. Furthermore, the impact of changing the *input resolution* is investigated. Afterward, the *loss function* for quantifying the FCN’s error is optimized, particularly by comparing distribution-based loss and a region-based loss. We pick the Dice loss for region-based loss and the Cross-Entropy loss as a distribution-based loss function. We also test the Focal loss, which is a variation of Cross-Entropy and suitable for imbalanced class scenarios [33]. Finally, 2 different optimizers for minimizing the loss function are evaluated. One is Adam [58], a very common optimizer, and the other one is a combination of some more modern optimizers, RAdam [59] and Lookahead [60]. For the backbone, the optimization is initiated with a default setting (i.e., transfer learning, no data augmentation, input resolution half of original image, Cross-Entropy loss function, and Adam optimizer). These initial parameters are then individually optimized in consecutive order.

### Data augmentation

Data augmentation is a method to generate additional training data via transformations of the original dataset. Since this increases the total amount of training data, data augmentation can improve performance and avoid overfitting [61]. In our case, every original training image is transformed once, doubling the size of the original training dataset. For each image in the training dataset, 1 of 3 transformations is picked randomly using Albumentations [61] (i.e., vertical flip, horizontal flip, or rotation by $180^\circ$). These transformations are valid since the spheroids do not have a specific orientation within an image. Note that each of these transformation conserves the original rectangular resolution of the image. This avoids extrapolating pixels on the border of the image, which would be necessary for arbitrary rotation angles and can lead to additional image artifacts the FCN model would have to be trained for.

## Results

### Hyperparameter optimization

For each hyperparameter set and preprocessing method, a model is trained, and the performance of these trained models is compared for the validation dataset based on the proposed evaluation metrics from the “Evaluation metrics” section. For JCD, RDD, and RCD, not only the average value but also the standard deviation over the whole validation dataset is reported to estimate reliability of the segmentation. For the ISF and the ASF, the standard error of the mean of the corresponding binomial distribution is reported. All results of the optimization are listed in Supplementary Figs. S5–S14 and briefly discussed in the following.

#### Backbones

For the U-Net, the ResNet 34 and the VGG 19-bn architecture perform best (see Supplementary Fig. S5), with a JCD 3 times smaller than the worst-performing backbones, like AlexNet or SqueezeNet1.0. The 2 ResNet backbones stand out with a very low ASF. Note that the accuracy of the VGG architectures is improving with an increasing number of used layers. The ResNet 34 is the optimal backbone as it not only performs best in virtually every metric but also requires less computational time than the VGG 19-bn. Note that a previous study on deep learning models for spheroid segmentation observed a higher performance for ResNet 18 compared to VGG 19 and ResNet 50 [29]. For the HRNet, the backbones differ in the number of kernels used, and the accuracy increases with the number of kernels (see Supplementary Fig. S6). The W48 backbone is preferred, as for the majority of metrics, it performs best and has the lowest standard deviations.

#### Data augmentation and transfer learning

The combination of data augmentation and transfer learning leads to the best performance for both U-Net and HRNet (see Supplementary Fig. S7 and Supplementary Fig. S8). While this is in principle expected, the improvement is substantial as the JCD is more than halved when both transfer learning and data augmentation are introduced. Note that for the U-Net, transfer learning improves accuracy more than data augmentation, while the opposite is true for the HRNet.

#### Resolution

Using input images with half the resolution $650 \times 515$ of the original images leads to the best performance (see Supplementary Fig. S9 and Supplementary Fig. S10). This input resolution is already used to optimize the previous parameters. Higher resolution (3/4, 1) reduces accuracy, presumably because the increase in details makes generalization of the appearance of spheroids more difficult, and the receptive fields of the kernels are getting smaller relative to the whole image. Lower resolution (1/4) also reduces accuracy, presumably due to the loss of relevant information. Half resolution displays the best accuracy and lowest standard deviations across the validation dataset.

#### Loss

We find the Dice loss to be the optimal loss function for the U-Net (see Supplementary Fig. S11). Note that Dice is considered the default loss parameter when using the U-Net for cell segmentation (i.e., binary classification concerned with the accuracy of the edge). However, the advantage is much smaller than for previous hyperparameters. It does not suggest a general advantage of region-based loss for the segmentation of spheroids by the U-Net. For the HRNet, there is a more significant difference between the region-based loss and the distribution-based loss (see Supplementary Fig. S12). The Focal loss and the Cross-Entropy loss perform better than the Dice loss—especially, the standard deviations differ by a factor of up to 3. One reason for this behavior may be that the Dice loss provides less detailed feedback during training than the distribution-based losses. Cross-Entropy loss and Focal loss achieve nearly the same results, and we choose the default Cross-Entropy loss.

#### Optimizer

In case of the U-Net, the difference between the tested optimizers is marginal (see Supplementary Fig. S13). We choose the slightly better-performing RAdam combined with Lookahead over the default optimizer Adam. In contrast, the accuracy of the HRNet is much worse for the combined optimizer (see Supplementary Fig. S14). The Adam optimizer is optimal with evaluation metrics 1.5 times better than for the RAdam combined with Lookahead.

### U-Net vs. HRNet

The automatic segmentations with the final optimized U-Net and HRNet model are applied to the test dataset. While this test dataset is not taken into account during training of the models, the segmentations display virtually the same high accuracy on these new images as for the validation dataset (see Table 1A and Supplementary Fig. S15; cf. Supplementary Fig. S13 and Supplementary Fig. S14). Mostly, the standard deviations of the metrics are higher in the test dataset, although the JCD remains even with this deviation well below $20 \%$. As for the validation dataset, the U-Net performs slightly better than the HRNet on the test data. The JCD and RDD for the HRNet are 0.01 higher, and their standard deviation is 0.04 higher. Two representative examples of the automatic segmentation by the U-Net are shown in Fig. 1: the overlap with the manual segmentation is excellent for standard size and larger spheroids obscured by cell debris (see Fig. 1, top row) and sufficient for small, heavily obscured spheroids (see Fig. 1, bottom row). In the latter case, the model does not segment the spheroid boundary accurately but detects the spheroid at the correct position JCD $= 23 \%$ with a comparable size $\text{RDD} = 7.5 \%$ within the much larger and often darker cloud of surrounding cell debris. In such difficult cases, similar variations are observed upon manual segmentation by different biological experts.

**Table 1:** (A) Evaluation of the segmentation with the optimized U-Net and HRNet models on the test dataset shows higher accuracy of the U-Net. Note that all metrics can range between 0 and 1, where lower values mean higher accuracy and 0 means perfect agreement with the manual segmentation. Bold values highlight the optimum for each metric. (B) Evaluation of the optimized segmentations on images of untreated (not irradiated) spheroids shows high accuracy also for the case of clean images without cell debris. The optimized U-Net and HRNet achieve almost equal accuracy. (C) The optimal hyperparameter configurations.

Model	JCD	RDD	RCD	ISF	ASF
(A) Segmentation test dataset					
U-Net	**0.062**	**0.020**	0.040	**0.000**	**0.010**
$\pm$	0.060	0.026	0.034	0.000	0.010
HRNet	0.072	0.028	**0.036**	**0.000**	**0.010**
$\pm$	0.100	0.080	0.029	0.000	0.010
(B) Segmentation images without cell debris					
U-Net	**0.028**	**0.008**	0.034	**0.000**	**0.000**
$\pm$	0.010	0.008	0.032	0.000	
HRNet	0.029	**0.008**	**0.033**	**0.000**	**0.000**
$\pm$	0.011	0.007	0.026	0.000	

Parameter	U-Net	HRNet
(C) Optimized parameters		
Backbone	ResNet 34	W48
Transfer learning	Yes	
Data augmentation	Yes	
Resize factor	1/2	
Loss function	Dice	Cross-Entropy
Optimizer	RAdam & Lookahead	Adam

While the models are mainly trained on images with treated spheroids that are obscured by cell debris, they also accurately segment untreated spheroids with a clear boundary and a clean background (see Table 1B): on a test set of 100 images of untreated spheroids, the accuracy of both models is nearly the same with a JCD below $3\%$. In comparison, a recent deep learning approach for spheroids with a clear boundary and a clean background with a focus on generalizability to different experimental conditions and microscopes achieved a mean JCD of $8\%$ with a standard deviation of $12\%$ for bright-field microscopic images [31]. This suggests that the standard case of clean images without cell debris is included in our training of images with heavily obscured spheroids.

The final U-Net model has a size of 158 MB and the final HRNet model a size of 251 MB. For both models, it takes about 1.8 seconds to segment 1 image on the CPU (Intel Core i7-4770). When the segmentation is performed serially on the GPU (NVIDIA GeForce RTX 3080), the U-Net needs only 0.03 seconds per image and the HRNet 0.08 seconds. As computation time for both models is comparable, the U-Net is chosen for the automatic segmentation due to its slightly higher accuracy in our setting. Note that in a recently published deep learning approach, the comparison of HRNet and U-Net implied that HRNet achieved the highest accuracy [31].

### Further independent validation

**Figure 4:**
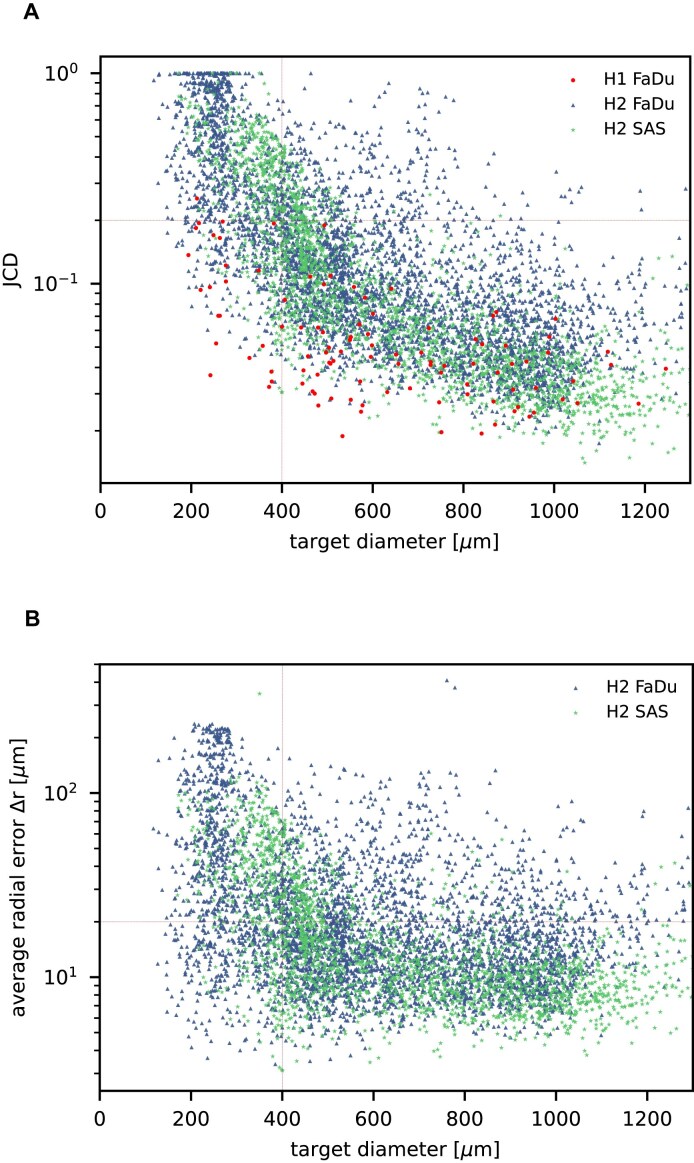
Validation of the automatic segmentation with the optimized U-Net on larger, independent datasets shows high accuracy for most cases. (A) JCD and (B) average radial error $\Delta r$ over diameter of the manually segmented (target) spheroid $d_T$ for 6,574 images of FaDu (blue triangles) and SAS (green stars) spheroids treated with different combinations and doses of X-ray irradiation and hyperthermia [16]. Manual segmentation is performed by a second biological expert (human H2, blue triangles and green stars) independently from the manual segmentation (human H1, red dots) of the training, validation, and test datasets. (Results for 104 images of the test dataset are displayed as red dots for comparison.) Note that the segmentation is developed only based on images of FaDu spheroids. Most deviations are small ($\text{JCD}<0.2$, $\Delta r < 20\ \mu$m, red horizontal lines as guide to the eye), average (median) values are $\text{JCD} = 0.17 (0.09) \pm 0.2$, $\Delta r = 25 (15) \pm 32\ \mu$m for the whole dataset, and $\text{JCD} = 0.1 (0.07) \pm 0.09$, $\Delta r = 18 (13) \pm 17 \ \mu$m for spheroids larger $d_T\ge 400\ \mu$m (red vertical lines as guide to the eye) than the initial, standard size of spheroids. Larger imprecisions for smaller spheroids are due to biologically difficult, unclear, or ambiguous cases; see text.

In addition to the standard check with the Hold-out test dataset, we systematically validate the automatic segmentation with the optimized U-Net on larger, independent Hold-out test datasets of 2 cell types of head and neck cancer (see Fig. 4). This dataset contains 6,574 images of FaDu or SAS spheroids treated with different combinations and doses of X-ray irradiation and hyperthermia [16]. These images were manually segmented by another biological expert (human H2) independent from the original training/validation/test-dataset used to develop the automatic segmentation (human H1). For most images, we find excellent overlap between this manual and the automatic segmentation, quantified by a JCD around 0.1 (see Fig. 4A). Larger deviations are mostly observed for images with smaller spheroids, that is, below the size of standard spheroids for treatment (diameter $370-400\ \mu$m according to [15–17]). To make the evaluation intuitive for the biological experts, who are less familiar with the JCD defined in Eq. (1), we additionally introduce a measure of the average radial error $\Delta r$ based on the automatically segmented (predicted) domain *P* and the manually segmented (target) domain *T*:


(3) \begin{eqnarray*}
\Delta r = \sqrt{\pi ^{-1}\left(|P \cup T|-|P \cap T|\right) + \frac{d_T^2}{4}}-\frac{d_T}{2}
\end{eqnarray*}

with the average (target) diameter $d_T$ of the manually segmented domain. The error $\Delta r$ quantifies the thickness a circular layer with the size of the mismatched area $|P \cup T|-|P \cap T| = |P\setminus T| + |T\setminus P|$ around a circle with the target area $|T|$ would have (see Supplementary Fig. S16 for an illustration). Since additional $P\setminus T$ and missing areas $T\setminus P$ do not compensate each other in Eq. (3), the error $\Delta r$ is considerably larger than the radial error implied by the relative difference between target and predicted diameter RDD (see Supplementary Fig. S17). The average radial error $\Delta r$ reported in Fig. 4B assesses the segmentation analogous to the JCD with the majority of errors $\Delta r < 20\ \mu$m and larger deviations mostly for spheroids with diameter clearly smaller than before treatment ($d_T\le 370-400\ \mu$m). For reference, note that the resolution of the images is $2.04 \ \mu \text{m}/\text{px}$; that is, a deviation of $20\ \mu \text{m}$ corresponds to 10px ($<1$% deviation with respect to the original 1,300 $\times$ 1,030 image) or 5px in the half-resolution image for application of the U-Net.

**Figure 5:**
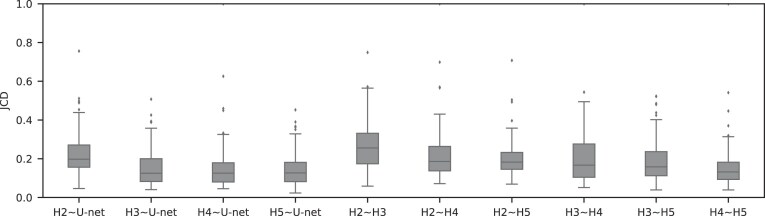
For treated spheroids smaller than the initial, standard size before treatment ($d\le 400\ \mu$m), deviations from the manual segmentation are not higher than variations across segmentations by different humans, suggesting that the segmentation of images with small spheroids surrounded by heavy debris is often difficult or ambiguous: compared are segmentations from the optimized U-Net and 4 independent human experts (H2–H5) for the same 101 images, which are randomly selected from the pool of small spheroids of the extended Hold-out test dataset; see Fig. 4. The Friedman test score of all JCDs 217.2 ($p \ll 0.001$) indicates significant differences among the pairwise segmentation deviations. The order according to the average JCD is (from low to high values) H5$\sim$U-Net, H3$\sim$U-Net, H3$\sim$H5, H2$\sim$H5 $<$ H4$\sim$H5, H4$\sim$U-Net, H2$\sim$U-Net, H3$\sim$H4, H2$\sim$H4, H2$\sim$H3, where the $<$ indicates a significant ($p<0.005$) difference between the sets of JCDs according to a Dunn–Bonferroni pairwise post hoc test.

Only a few images with larger spheroids exhibit JCD bigger than 0.2. Most of these are ambiguous cases, with 2 spheroids attached to each other, which are inconsistently recognized as either 1 or 2 spheroids even by the human; see examples with $\text{JCD}= 0.28-0.34$ and 0.42 in Supplementary Fig. S4. While the training dataset did not contain such double-spheroid cases, the optimized U-Net often segments them correctly; see examples with $\text{JCD}=0.08, 0.12$ in Supplementary Figs. S2 and S3, respectively. Apart from this particular case, most images with larger deviations between the segmentations refer to smaller spheroids with surrounding cell debris. Note that smaller spheroids without cell debris (i.e., untreated or shortly after treatment) do not exhibit such deviations but are accurately segmented. To thoroughly examine these larger deviations in more detail, 3 biological experts independently segmented 101 randomly selected images with spheroid diameter $d_T\le 400\ \mu$m. For these 101 images, the JCDs between the humans and the U-Net and between different humans are compared in Fig. 5. We find that on average, the discrepancies between humans and U-Net are comparable to the variations across segmentations from different humans. This implies that these images represent biologically difficult, unclear, or ambiguous cases. It suggests that the larger JCD observed for some smaller spheroids rather reflects this uncertainty and not a low performance of the U-Net. Accordingly, the evaluation in Fig. 4 refers to a worse-case scenario, as for each spheroid, the biological expert intentionally segmented all images over time, while in practice, segmentation is only required and performed for a fraction of these images, in particular for clearly distinguishable and larger spheroids.

Finally, we also test the automatic segmentation with the optimized U-Net on 8 published test datasets from previous deep learning approaches [22, 29, 31]; see Supplementary Table S2. Note that the total of 496 images originate from different conditions, including bright-field and fluorescence microscopy, RGB and 16-bit gray-scale, and different microscopes, magnifications, image resolutions, and cell models, but do not contain significant cell debris. While our automatic segmentation performs well on roughly half of the datasets, sometimes surpassing the original model corresponding to the dataset, 2 types of images turn out problematic: (i) images with ambiguous ground truth and (ii) images on which the spheroids appears semi-transparent, with individual cells being visible throughout the spheroid. However, classical segmentation techniques work sufficiently well for both types of images (i) and (ii), making the use of deep learning approaches in these cases unnecessary. In detail, images of type (i) contain spheroids with peculiar intensity profiles (i.e., with a compact sphere-like core surrounded by a flat patch). The corresponding published ground truth just defines the outer boundary of these patches as the boundary of the spheroid, while our automatic segmentation restricts the spheroid to the compact core. Either choice may be unreasonable depending on the goal of the analysis. The outer boundary of the patches allows computation of the projected area of the spheroid while ignoring the considerable variations in thickness encoded in the intensity across the spheroid. Accordingly, it is not suited to estimate the spheroid volume necessary to evaluate volume growth. Presumably, the compact core contains most of the 3D organized cells and cellular volume. It is the main component exhibiting metabolic gradients affecting therapy response [4–8], but certainly, it underestimates the total cell volume. Segmentation of such incompletely formed spheroids is debatable, even biologically, as the formation of proliferative and metabolic gradients remains ambiguous and may depend on the question pursued with the experiment. For images of type (ii), the apparent visibility of individual cells throughout the spheroid may be due to its small size or the chosen microscopy method.

## Discussion

We develop an automatic segmentation for images of 3D tumor spheroids both with and without (radio)therapy. We systematically validate the automatic segmentation on larger, independent Hold-out test datasets of 2 cell types of head and neck cancer spheroid types, including combinations with hyperthermia treatment. For most images, we find excellent overlap between manual and automatic segmentation. These include clearly discernable spheroids and the previously neglected cases of spheroids critically obscured by cell debris. For images showing poor overlap of the segmentations, we demonstrate that this error is comparable to the variations between segmentations from different biological experts (interobserver variability). This suggests that considerable deviations between automatic and manual segmentations do not necessarily reflect a low performance of the former but rather a general uncertainty or ambiguity in spheroid identification.

While the accuracy of (spheroid) segmentations is usually only quantified in terms of the JCD or Jaccard index (IoU), we choose to additionally report the corresponding implications on the spheroid diameter derived from the segmentation by $\Delta r$ and RDD. This makes the evaluation more intuitive for biological experts, as the spheroid diameter is the central metric for analysis. For instance, the spheroid volume required for growth curve documentation and growth delay is estimated from this diameter, which is connected to the projected area of the spheroid. We do not explicitly translate the evaluation to the corresponding spheroid area and volume. However, this may be estimated from basic scaling arguments (i.e., area and volume scale square and cubic with the diameter). The circularity of the spheroid, as quality control for its desired spherical shape, is taken into account by the RCD. All of these metrics will converge to their correct value, when the JCD goes to 0 (or Jaccard index to 1).

In fact, it should be pointed out that the imprecisions of the segmentation have a smaller effect on the quantification of therapy response than suggested by the reported JCD: as most informative parameters in long-term spheroid-based assays are the average diameters, volumes, and circularities of the spheroids. Thus, the relevant metric to estimate the error of the automatic analysis is not the JCD but the RDD and RCD, which are considerably smaller as even an imprecise segmentation can result in the correct size or shape of the spheroid. Furthermore, higher deviations are primarily observed for for treated 3D cultures with diameters below the initial diameter of standard spheroids $d_T= 370-400 \ \mu$m before treatment. These are usually images of spheroids in the final steps of detachment or before cell reaggregation, growth recovery, and spheroid relapse. In experimental practice, such cases are typically not segmented as they are less relevant for the analytical endpoints. For instance, computation of growth delay requires accuracy of the spheroid segmentation immediately before treatment (for images without obscuring cell debris) and at large spheroid sizes $d_T>600 \ \mu$m, while the images in between have no impact on the growth delay. Moreover, the validation in Fig. 4 is based on images from the time series of relapsed and controlled spheroids. However, in practical routine, segmentations of complete time series are only performed for growth curves and growth delay assessment in spheroid populations at 100% growth recovery. The average JCD is substantially smaller if the validation is restricted to these cases.

It is important to note that the initial automatic segmentation is driven by images of only 1 HNSCC spheroid model (FaDu) with and without treatment. However, we show that it works equally well for spheroids from another cell type (SAS), although these SAS spheroids display a different peripheral shape during regrowth after treatment. Beyond the systematic validation highlighted herein, the automatic segmentation has been continuously tested and applied by several biological/biomedical experts and researchers for over a year during their ongoing experiments and to retrospectively reanalyze MCTS from earlier studies. So far, it has been reported that resegmentation is only necessary in a small fraction of cases, mainly due to optical artifacts, like out-of-focus images. Overall, the segmentation has in the meantime been successfully applied to numerous untreated multicellular spheroid types of different tumor entities and cell lines, respectively (FaDu, SAS—head and neck; Panc-02(mouse), Panc-1, PaTu 8902—pancreas; HCT-116, HT29—colon; A549, NCI-H23, NCI-H460—lung; BT474—breast; LNCap, DU145—prostate; U87-MG, U138-MG, U251-MG—brain/GBM; Hek293—kidney). These spheroids ranged between $200 - 1000 \ \mu$m in diameter and images were taken at different magnifications and resolutions (e.g., $1300 \times 1030$, $1388\times 1040$  $1920 \times 1440$, $1920 \times 1216$, and $1.6 - 2.6 \ \mu$m/pixel) as single or Z-stack images at diverse microscopic devices (Axiovert 200M, AxioObserver Z1—both from Zeiss; BioTek Cytation 5 Cell Imaging Multimode Reader—Agilent). Many more spheroid types are in the pipeline for implementation. The developed automatic segmentation has also already been applied to contour the images of selected spheroid types (e.g., FaDu, SAS, Panc-02, or DU145) after various treatments such as radiotherapy (X-ray, proton), hyperthermia, chemotherapy, and combinatorial treatment.

We also test the automatic segmentation on 8 published test datasets from previous deep learning approaches [22, 29, 31]. We observe a high accuracy, except for special cases, which, however, turn out to be well segmentable by classical techniques. While the automatic segmentation has been continuously tested for over a year on different cell lines, microscopes, and treatments, it does not necessarily generalize to arbitrary experimental conditions and imaging, which will be the focus of future improvements. However, this problem of domain shift, which can always arise when a model is applied to datasets with a different data distribution than the training data, is also an ongoing challenge for deep learning models focused on images with clear spheroids and clean background [49]. In contrast, the focus of this study is the inclusion of the case of treated spheroids surrounded by severe cell debris, which is frequent after treatment but often neglected in automatic segmentation.

Note that the automatic segmentation’s accuracy and reliability may not be exclusive to the finally chosen network architecture and hyperparameters. Indeed, we find that 2 very different network architectures, U-Net and HRNet, achieve very similar performance, which is consistent with previously reported optimizations of deep learning models  [31, Tab. 2] [29, Tab. 3]. The segmentation’s accuracy also seems relatively insensitive to the choice of several hyperparameters (e.g., loss function, optimizer function, and, to some extent, the resize factor). Instead, it is plausible that applying challenging training data (i.e., images with extensive, severe debris) is crucial for the performance of the resulting automatic segmentation. The network trained primarily on such images also works on images with clearly visible spheroids. Hence, the datasets compiled and annotated for this work are also publicly provided to support future improvements of spheroid segmentations. This dataset can facilitate the development of more individual, more customized models (e.g., using the recently introduced nnU-Net tool [62]).

The automatic segmentation can be incorporated into existing or future tools for spheroid analysis or medical image analysis as basis for further feature extraction, including perimeter, complexity, and multiparametric analysis [47]. The segmentation provides a basis for the development of an automatic classification of spheroid image time series into control and relapse and could support machine learning methods to forecast tumor spheroid fate early. To make the deep learning model available, we provide a minimal tool with graphical user interface. The ONNX Runtime [63] is used to compile the model and to take it into production.

## Availability of Source Code and Requirements

- Project name: Spheroidsegdedeb (Spheroid segmentation despite debris)
- Project homepage: https://igit.informatik.htw-dresden.de/aagef650/spheroidsegdedeb
- Operating system(s): Platform independent
- Programming language: Python >3.8.10
- Other requirements: Additionally download the model from https://wwwpub.zih.tu-dresden.de/~s6079592/SEGMODEL.onnx. Requirements and usage are documented in the provided README, and some example images for testing are included in the corresponding folder.
- License: GNU GPL
- BioTool ID: spheroidsegdedeb
- SciCrunch ID: SpheroidSegDeDeb, RRID:SCR_026409
- SpheroidSegDeDeb is also archived in Software Heritage [64].

## Supplementary Material

giaf027_Supplemental_File

giaf027_Authors_Response_To_Reviewer_Comments_Original_Submission

giaf027_Authors_Response_To_Reviewer_Comments_Revision_1

giaf027_GIGA-D-24-00570_Original_Submission

giaf027_GIGA-D-24-00570_Revision_1

giaf027_GIGA-D-24-00570_Revision_2

giaf027_Reviewer_1_Report_Original_Submission Mariachiara Stellato -- 2/3/2025

giaf027_Reviewer_2_Report_Original_Submission Jónathan Heras -- 2/4/2025

## Data Availability

Training, validation, and test data used for this work are available [65], including data on clean spheroids and the extended dataset as well as the code to train and evaluate the model (see above repo training_scripts/). All additional supporting data are available in the *GigaScience* repository, GigaDB [66] (i.e., all networks optimized for different hyperparameters in the.pth format). DOME-ML annotations are available in DOME registry [67].

## Additional Files

**Supplementary Fig. S1**. Example images illustrating the current challenge of segmenting tumor spheroids: (a) While previously developed deep learning models generalize excellently to new image data with well-distinguished, unobscured spheroids, typical for untreated cultures, (b) these models fail for cases of detached/relapsing spheroids after radiotherapy (one of the most common cancer treatments) due to debris and dead cells obscuring the spheroid. Shown are representative images of FaDu spheroids (a) without treatment and (b) several days after radiotherapy. Different segmentations are indicated by their outer contours. In particular, the manually set ground truth (green solid line) is compared to the four most recent deep learning models provided by SpheroidJ [31] (HRNet-Seg with HRNet W30 backbone, yellow close-dashed line), SpheroScan [48] (region-based convolutional neural network trained on images from IncuCyte Live-Cell Analysis System [purple dashed line] and ordinary microscope [torquoise dashed line] with recommended threshold 0.8), and AnaSP [29] (ResNet18, red dotted line). Only the U-Net presented in this article (blue solid line) performs sufficiently in both scenarios. Size of each image corresponds to $2,650 \ \mu \text{m} \times 2,100 \ \mu \text{m}$ with a resolution of 1,300 $\times$ 1,030 pixels.

**Supplementary Fig. S2**. Selection of representative images as optical reference for JCD$\le 0.1$ with automatic segmentation from the optical U-Net (orange) and manual segmentation from biological expert H2 (green). From the tested images, $52 \%$ fall into this range of JCD$\le 0.1$ and $67 \%$ when only spheroids beyond the standard size $d_T>400 \ \mu$m are considered. Size of each image corresponds to $2,650 \ \mu \text{m} \times 2,100 \ \mu \text{m}$. Note that the U-Net is trained on independent manual segmentations from another biological expert H1. Images are selected from the extended validation dataset (Fig. 4), in particular, from the subset of larger, better discriminated spheroids at intermediate levels of debris for illustration. Note that in the example for $\text{JCD}=0.08$, the 2 attached spheroids are correctly segmented, although the training dataset does not contain such cases.

**Supplementary Fig. S3**. Selection of representative images as optical reference for $0.1<\text{JCD}\le 0.2$ with automatic segmentation from the optical U-Net (orange) and manual segmentation from biological expert H2 (green), analogous to Supplementary Fig. S2. From the tested images, $22 \%$ fall into the range $0.1<\text{JCD}\le 0.2$ and $23 \%$ when only spheroids beyond the standard size $d_T>400 \ \mu$m are considered. Note that in the example of $\text{JCD}=0.12$, the 2 attached spheroids are correctly segmented, although the training dataset does not contain such cases.

**Supplementary Fig. S4**. Selection of representative images as an optical reference for larger deviations $\text{JCD}> 0.2$ of the automatic segmentation from the optical U-Net (orange) and manual segmentation from biological expert H2 (green), analogous to Supplementary Fig. S2. From the tested images, $26 \%$ fall into the range $\text{JCD}> 0.2$, but merely $10 \%$ when only spheroids beyond the standard size $d_T>400 \ \mu$m are considered. Note that deviations at larger spheroids are often due to cases of double spheroids (e.g., $\text{JCD}=0.27, 0.28, 0.29, 0.34, 0.42$), which are inconsistently recognized as either 1 or 2 spheroids even by the human.

**Supplementary Fig. S5**. Values of evaluation metrics for different backbones of the U-Net. Bold values highlight the optimum in each column. The other hyperparameters are fixed at Optimizer: Adam, Loss: Cross-Entropy, Resize factor: 1/2, Transfer learning: Yes, Data augmentation: No. Overall, the ResNet 34 achieved the best results and is picked as the backbone for the U-Net.

**Supplementary Fig. S6**. Values of evaluation metrics for different backbones of the HRNet. Bold values highlight the optimum in each column. The other hyperparameters are fixed at Optimizer: Adam, Loss: Cross-Entropy, Resize factor: 1/2, Transfer learning: Yes, Data augmentation: No. Overall, the W48 achieved the best results and is picked as the backbone for the HRNet.

**Supplementary Fig. S7**. Values of evaluation metrics for different extensions of the training dataset, which is used by the U-Net. Bold values highlight the optimum in each column. The other hyperparameters are fixed at Backbone: ResNet 34, Optimizer: Adam, Loss: Cross-Entropy, Resize factor: 1/2. The accuracy of the U-Net is highest when transfer learning and data augmentation are used.

**Supplementary Fig. S8**. Values of evaluation metrics for different extensions of the training dataset, which is used by the HRNet. Bold values highlight the optimum in each column. The other hyperparameters are fixed at Backbone: W48, Optimizer: Adam, Loss: Cross-Entropy, Resize factor: 1/2. The accuracy of the HRNet is highest when transfer learning and data augmentation are used.

**Supplementary Fig. S9**. Values of evaluation metrics for different image sizes. Bold values highlight the optimum in each column. The other hyperparameters are fixed at Backbone: ResNet 34, Optimizer: Adam, Loss: Cross-Entropy, Transfer learning: Yes, Data augmentation: Yes. The U-Net can achieve the highest accuracy when the original image size is reduced by half.

**Supplementary Fig. S10**. Values of evaluation metrics for different image sizes. Bold values highlight the optimum in each column. The other hyperparameters are fixed at Backbone: W48, Optimizer: Adam, Loss: Cross-Entropy, Transfer learning: Yes, Data augmentation: Yes. The HRNet can achieve the highest accuracy when the original image size is reduced by half.

**Supplementary Fig. S11**. Values of evaluation metrics for different loss functions, used by the U-Net. Bold values highlight the optimum in each column. The other hyperparameters are fixed at Backbone: ResNet 34, Optimizer: Adam, Resize factor: 1/2, Transfer learning: Yes, Data augmentation: Yes. The U-Net can achieve the highest accuracy when the Dice loss is used.

**Supplementary Fig. S12**. Values of evaluation metrics for different loss functions, used by the HRNet. Bold values highlight the optimum in each column. The other hyperparameters are fixed at Backbone: W48, Optimizer: Adam, Resize factor: 1/2, Transfer learning: Yes, Data augmentation: Yes. The HRNet can achieve the highest accuracy if the Cross-Entropy or the Focal loss is used.

**Supplementary Fig. S13**. Values of evaluation metrics for different optimizers, used by the U-Net. Bold values highlight the optimum in each column. The other hyperparameters are fixed at Backbone: ResNet 34, Loss: Dice, Resize factor: 1/2, Transfer learning: Yes, Data augmentation: Yes. The U-Net can achieve the highest accuracy when the optimization is done by RAdam combined with Lookahead.

**Supplementary Fig. S14**. Values of evaluation metrics for different optimizers, used by the HRNet. Bold values highlight the optimum in each column. The other hyperparameters are fixed at Backbone: W48, Loss: Cross-Entropy, Resize factor: 1/2, Transfer learning: Yes, Data augmentation: Yes. The HRNet can achieve the highest accuracy when the optimization is done by Adam.

**Supplementary Fig. S15**. Evaluation of the segmentation with the optimized U-Net and HRNet models on the test dataset shows higher accuracy of the U-Net. The optimal hyperparameter configuration for the U-Net is Backbone: ResNet 34, Optimizer: RAdam & Lookahead, Loss: Dice, Resize factor: 1/2, Transfer learning: Yes, Data augmentation: Yes. The final hyperparameter configuration for the HRNet is Backbone: W48, Optimizer: Adam, Loss: Cross-Entropy, Resize factor: 1/2, Transfer learning: Yes, Data augmentation: Yes.

**Supplementary Fig. S16**. Illustration of the average radial error $\Delta r$ defined in Eq. (3) (example with large $\text{JCD} > 0.2$ for visibility). (a) Zoom of image from Supplementary Fig. S4 with $\text{JCD} = 0.24$ with corresponding automatic (orange line containing predicted area *P*) and manual segmentation (green line containing target area *T*). (b) Segmentations from (a) with mismatched areas shown in gray (missing area $T\setminus P$ in bright and dark gray, additional area $P\setminus T$ in intermediate gray). (c) Circle (green line) and added rim (gray areas) with areas corresponding to (b): area within green circle is equal to area within manual segmentation in (b), and gray areas within the adjacent rim are equal to corresponding mismatched areas in (b). Then the radial thickness of the rim is equal to the average radial error $\Delta r$ defined in Eq. (3).

**Supplementary Fig. S17**. Validation based on diameter of automatic segmentation with the optimized U-Net on larger, independent datasets, analogous to Fig. 4. (a) RDD shows that most deviations are below $10 \%$ and higher deviations occur mostly below $d_T < 400 \ \mu$m. (b) Direct comparison of diameters (gray points) resulting from automatic segmentation (predicted diameter) and manual segmentation (target diameter) shows high accuracy (red line represents perfect match) with larger deviations mostly at $d_T < 400 \ \mu$m.

**Supplementary Table S1**. Statistical results reflecting the current challenge of segmenting tumor spheroids, corroborating Supplementary Fig. S1. While previously developed deep learning models generalize excellently to new image data with well-visible, unobscured spheroids (top line), these models fail for typical cases of detached/relapsing spheroids with debris of dead cells (bottom lines). Intersection over Union (IoU or Jaccard index) is reported (average [median] $\pm$ standard deviation) for each of the 4 most recent deep learning models (provided by SpheroidJ [31], SpheroScan [48] [with recommended threshold 0.8], and AnaSP [29]) and each of our datasets. Only the U-Net presented in this article performs sufficiently in both scenarios. Bold values highlight the optimum for each dataset.

**Supplementary Table S2**. Further validation of the trained U-Net on a wide variety of published test datasets from previous deep learning models. While the trained U-Net performs well on on roughly half of the datasets or 38% of the images (with average IoU above 0.8), sometimes surpassing the original model corresponding to the dataset, 2 types of images turn out problematic: (i) images with ambiguous ground truth (20% of images) for which the U-Net may actually segment reasonably (see main text for detailed discussion) and (ii) images on which the spheroid appears semi-transparent (42% of images), with individual cells being visible throughout the spheroid, potentially due to its small size or the chosen microscopy method. However, classical segmentation techniques work sufficiently well for both types of images (i) and (ii), making the use of deep learning approaches in these cases unnecessary. For demonstration, the last 2 columns report the performance of the classical approach from the original publication and simple Otsu thresholding (sometimes after some image erosion for (ii)) performed by us for images of types (i) and (ii). Note that the notation for datasets from Refs. [22, 31] stands for brightfield/fluorescence microscopy (B/F), Nikon Eclipse/Leica DMi8/Olympus microscope (N,L,O), 2×/5×/10× magnification (2/5/10), and suspension/collagen culture (S/C). Bold values highlight the optimum for each dataset.

## Abbreviations

ASF: ambiguous spheroid fraction; FCN: fully convolutional network; ISF: invalid spheroid fraction; JCD: Jaccard distance; MCT: multicellular tumor spheroid; RCD: relative circularity deviation; RDD: relative diameter deviation; SM: supplemental material.
