# Supplementary material for: Image segmentation of treated and untreated tumor spheroids by fully convolutional networks
Source: Gigascience. 2025 May 7;14:giaf027. doi: 10.1093/gigascience/giaf027 (PMC12056507; doi:10.1093/gigascience/giaf027)
Supplement: giaf027_Supplemental_File [file giaf027_supplemental_file.pdf]

A Supplemental figures and tables (SM)

(a) Untreated spheroid w/o cell debris **(b)** Treated spheroid with cell debris

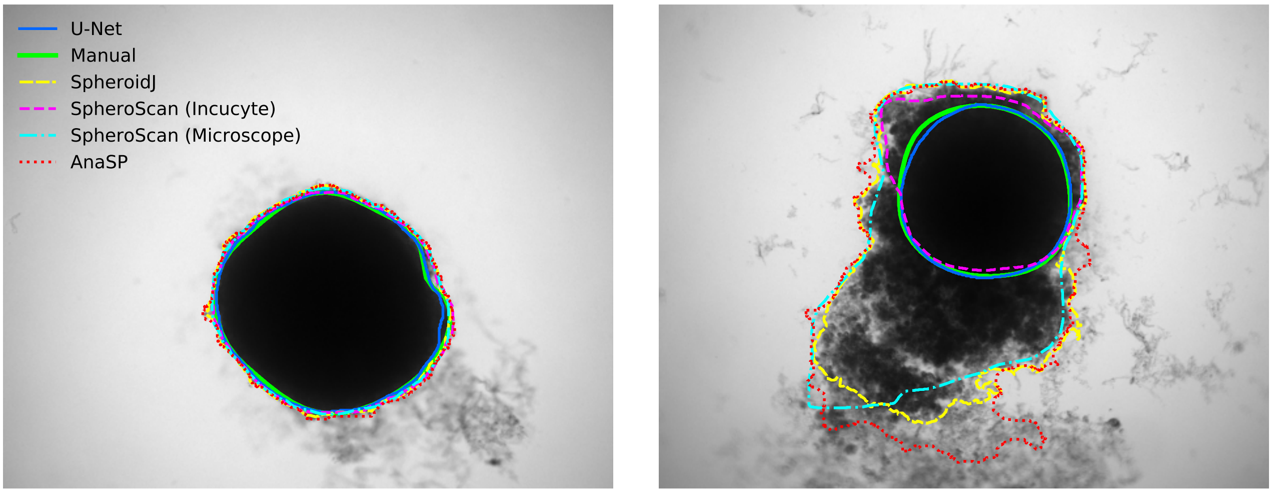

Figure SM.1. Example images illustrating the current challenge of segmenting tumor spheroids: (a) While previously developed deep-learning models generalize excellently to new image data with well-distinguished, unobscured spheroids, typical for untreated cultures, (b) these models fail for cases of detached/relapsing spheroids after radiotherapy (one of the most common cancer treatments) due to debris and dead cells obscuring the spheroid. Shown are representative images of FaDu spheroids (a) without treatment and (b) several days after radiotherapy. Different segmentations are indicated by their outer contours. In particular, the manually set ground truth (green solid line) is compared to the four most recent deep-learning models provided by SpheroidJ [31] (HRNet-Seg with HRNet W30 backbone, yellow close-dashed line), SpheroScan [48] (Region-based Convolutional Neural Network trained on images from IncuCyte Live-Cell Analysis System (purple dashed line) and ordinary microscope (torquoise dashed line) with recommended threshold 0.8), and AnaSP [29] (ResNet18, red dotted line). Only the U-Net presented in this manuscript (blue solid line) performs sufficiently in both scenarios. Size of each image corresponds to 2650 μm $\times$ 2100 μm with a resolution of 1300 $\times$ 1030 pixel.

Table SM.1. Statistical results reflecting the current challenge of segmenting tumor spheroids, corroborating Fig. SM.1: While previously developed deep-learning models generalize excellently to new image data with well-visible, unobscured spheroids (top line), these models fail for typical cases of detached/relapsing spheroids with debris of dead cells (bottom lines). Intersection over Union (IoU or Jaccard index) is reported (average (median) ± standard deviation) for each of the four most recent deep-learning models (provided by SpheroidJ [31], SpheroScan [48] (with recommended threshold 0.8), and AnaSP [29]) and each of our data sets. Only the U-Net presented in this manuscript performs sufficiently in both scenarios. Bold values highlight the optimum for each dataset.

Data set	#Images	Intersection over Union (IoU = 1-JCD)				
		U-Net	SpheroidJ [31]	SpheroScan (Incu) [48]	SpheroScan (Micro) [48]	AnaSP [29]
Spheroids without cell debris	200	**0.97(0.97)** ± **0.01**	0.89(0.90) ± 0.05	0.90(0.94) ± 0.16	0.72(0.92) ± 0.38	0.81(0.87) ± 0.22
Training	883	**0.94(0.95)** ± **0.05**	0.51(0.53) ± 0.27	0.68(0.86) ± 0.34	0.48(0.59) ± 0.37	0.46(0.44) ± 0.28
Validation	108	**0.95(0.96)** ± **0.04**	0.31(0.29) ± 0.19	0.59(0.80) ± 0.39	0.36(0.26) ± 0.32	0.28(0.25) ± 0.19
Test	104	**0.94(0.96)** ± **0.06**	0.41(0.38) ± 0.27	0.66(0.88) ± 0.36	0.48(0.50) ± 0.38	0.35(0.33) ± 0.24

Table SM.2. Further validation of the trained U-Net on wide variety of published test data sets from previous deep-learning models. While the trained U-Net performs well on on roughly half of the data sets or 38% of the images (with average IoU above 0.8), sometimes surpassing the original model corresponding to the data set, two types of images turn out problematic: (i) images with ambiguous ground truth (20% of images) for which the U-Net may actually segment reasonably, see main text for detailed discussion, and (ii) images on which the spheroids appears semi-transparent (42% of images), with individual cells being visible throughout the spheroid, potentially due to its small size or the chosen microscopy method. However, classical segmentation techniques work sufficiently well for both types of images (i) and (ii), making the use of deep-learning approaches in these cases unnecessary. For demonstration, the last two columns report the performance of the classical approach from the original publication and simple Otsu thresholding (sometimes after some image erosion for (ii)) performed by us for images of types (i) and (ii). Note that the notation for data sets from Refs. [22, 31] stands for brightfield/fluorescence microscopy (B/F), Nikon Eclipse/Leica DMi8/Olympus microscope (N,L,O), 2x/5x/10x magnification (2/5/10), and suspension/collagen culture (S/C). Bold values highlight the optimum for each dataset.

Data set	#Images	Comments	Intersection over Union (IoU = 1-JCD)			
			deep-learning model		classical segmentation	
			Original	U-Net	Original	Otsu thresholding
BO10S [22]	66		0.92 ± 0.03 [31]	**0.97(0.98)** ± **0.05**	0.94 ± 0.03 [31]	-
BN10S [31]	21<br>84	 ambiguous ground truth	**0.97** ± **0.01**	0.89(0.89) ± 0.01<br>0.61(0.62) ± 0.15	0.95 ± 0.01	0.84(0.85) ± 0.13
BN2S [31]	154	semi-transparent	**0.96** ± **0.01**	0.30(0.27) ± 0.22	0.94 ± 0.02	0.87(0.95) ± 0.27
BL5S [31]	50	semi-transparent	0.75 ± 0.25	0.37(0.13) ± 0.39	0.64 ± 0.30	**0.85(0.93)** ± **0.20**
FN2S [31]	30<br>4	 semi-transparent	0.78 ± 0.20	0.91(0.94) ± 0.08<br>0	**0.82** ± **0.17**	-<br>0.83(0.83) ± 0.02
FL5C[31]	4<br>15	 ambiguous ground truth	0.71 ± 0.30	0.92(0.91) ± 0.01<br>0.45(0.45) ± 0.16	0.67 ± 0.17	-<br>**0.80(0.85)** ± **0.11**
FL5S [31]	50		0.70 ± 0.26	**0.91(0.91)** ± **0.05**	0.89 ± 0.07	-
AnaSP [29]	16<br>2	 semi-transparent	~ 0.92	0.97(0.98) ± 0.02<br>0.01(0.01) ± 0.01		-<br>0.97(0.97) ± 0.001

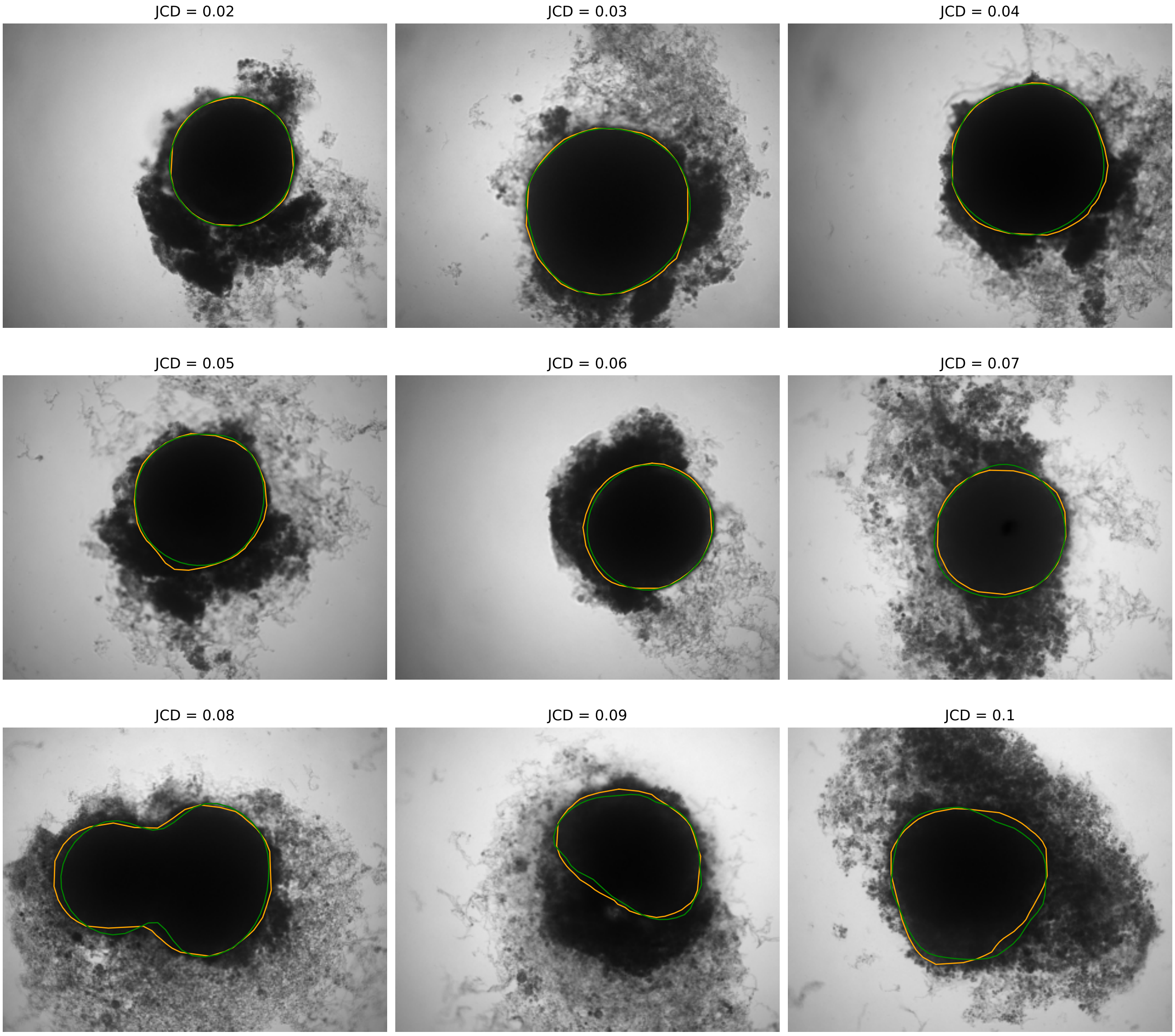

Figure SM.2. Selection of representative images as optical reference for JCD$\leq$ 0.1 with automatic segmentation from the optical U-Net (orange) and manual segmentation from biological expert H2 (green). From the tested images, 52% fall into this range of JCD$\leq$ 0.1 and 67% when only spheroids beyond the standard size d_T > 400 µm are considered. Size of each image corresponds to 2650 µm × 2100 µm. Note that the U-Net is trained on an independent manual segmentations from another biological expert H1. Images are selected from the extended validation data set (Fig. 4), in particular, from the subset of larger, better discriminated spheroids at intermediate levels of debris for illustration. Note that in the example for JCD = 0.08, the two attached spheroids are correctly segmented, although the training data set does not contain such cases.

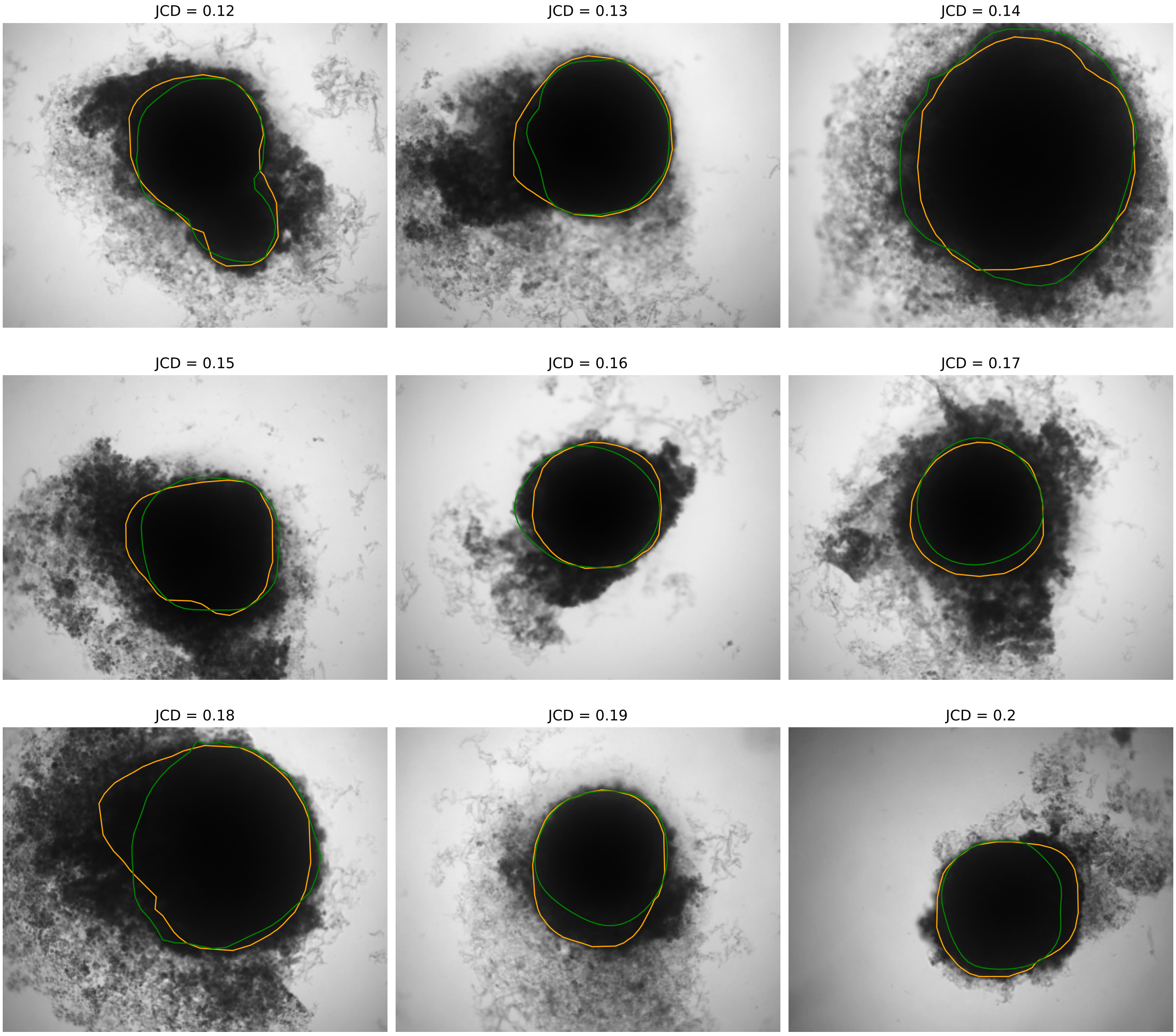

Figure SM.3. Selection of representative images as optical reference for 0.1 < JCD $\leq$ 0.2 with automatic segmentation from the optical U-Net (orange) and manual segmentation from biological expert H2 (green), analogous to Fig. SM.2. From the tested images, 22% fall into the range 0.1 < JCD $\leq$ 0.2 and 23% when only spheroids beyond the standard size $d_T > 400$ µm are considered. Note that in the example of JCD = 0.12, the two attached spheroids are correctly segmented, although the training data set does not contain such cases.

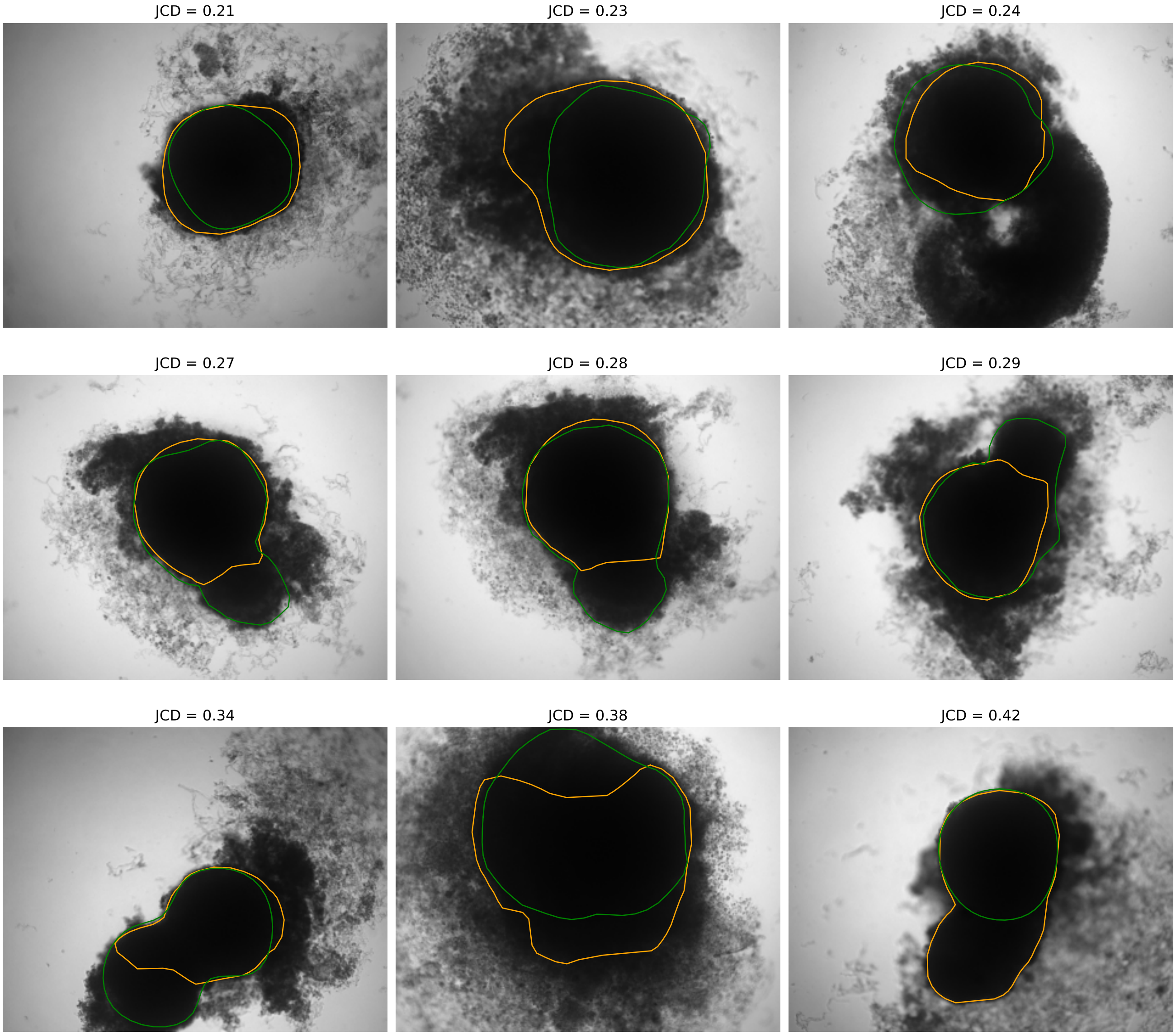

Figure SM.4. Selection of representative images as optical reference for larger deviations JCD > 0.2 of the automatic segmentation from the optical U-Net (orange) and manual segmentation from biological expert H2 (green), analogous to Fig. SM.2. From the tested images, 26% fall into the range JCD > 0.2, but merely 10% when only spheroids beyond the standard size $d_T > 400$ µm are considered. Note that deviations at larger spheroids are often due to cases of double-spheroids, e.g., JCD = 0.27, 0.28, 0.29, 0.34, 0.42, which are inconsistently recognized as either one or two spheroids even by the human.

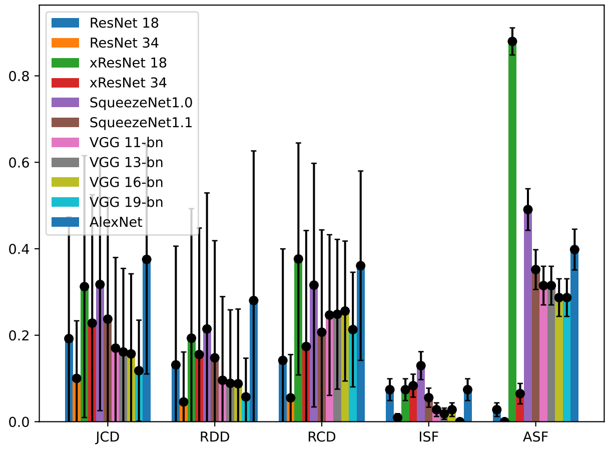

Backbone	JCD	RDD	RCD	ISF	ASF
ResNet 18	0.192	0.131	0.142	0.074	0.028
±	0.282	0.275	0.258	0.025	0.016
ResNet 34	**0.100**	**0.046**	**0.055**	0.009	**0.000**
±	0.133	0.116	0.100	0.009	0.000
xResNet 18	0.312	0.193	0.376	0.074	0.880
±	0.303	0.300	0.268	0.025	0.031
xResNet 34	0.228	0.155	0.174	0.083	0.065
±	0.297	0.292	0.268	0.027	0.024
SqueezeNet1.0	0.317	0.215	0.316	0.130	0.491
±	0.292	0.315	0.282	0.032	0.048
SqueezeNet1.1	0.237	0.148	0.207	0.056	0.352
±	0.281	0.271	0.237	0.022	0.046
VGG 11-bn	0.170	0.096	0.247	0.028	0.315
±	0.210	0.193	0.186	0.016	0.045
VGG 13-bn	0.161	0.089	0.248	0.019	0.315
±	0.193	0.170	0.173	0.013	0.045
VGG 16-bn	0.157	0.088	0.256	0.028	0.287
±	0.185	0.173	0.162	0.016	0.044
VGG 19-bn	0.118	0.057	0.213	**0.000**	0.287
±	0.117	0.090	0.133	0.000	0.044
AlexNet	0.375	0.280	0.361	0.074	0.398
±	0.265	0.346	0.219	0.025	0.047

Figure SM.5. Values of evaluation metrics for different backbones of the U-Net. Bold values highlight the optimum in each column. The other hyperparameters are fixed at Optimizer: Adam, Loss: Cross-Entropy, Resize factor: 1/2, Transfer learning: Yes, Data augmentation: No. Overall the ResNet 34 achieved the best results and is picked as the backbone for the U-Net.

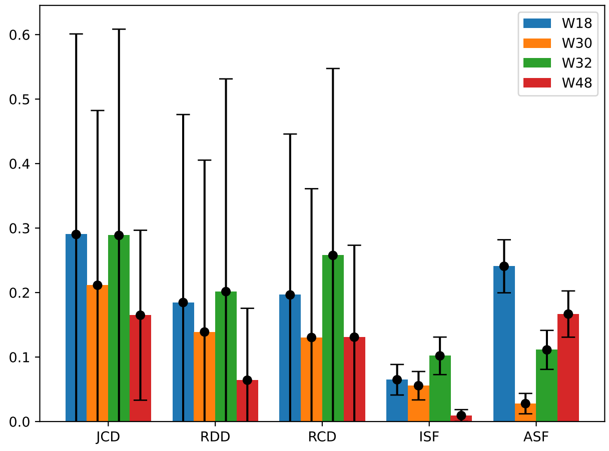

Backbone	JCD	RDD	RCD	ISF	ASF
W18	0.290	0.185	0.196	0.065	0.241
±	0.311	0.291	0.249	0.024	0.041
W30	0.211	0.139	**0.130**	0.056	**0.028**
±	0.271	0.266	0.231	0.022	0.016
W32	0.289	0.201	0.258	0.102	0.111
±	0.320	0.330	0.290	0.029	0.030
W48	**0.165**	**0.064**	0.131	**0.009**	0.167
±	0.132	0.111	0.143	0.009	0.036

Figure SM.6. Values of evaluation metrics for different backbones of the HRNet. Bold values highlight the optimum in each column. The other hyperparameters are fixed at Optimizer: Adam, Loss: Cross-Entropy, Resize factor: 1/2, Transfer learning: Yes, Data augmentation: No. Overall the W48 achieved the best results and is picked as the backbone for the HRNet.

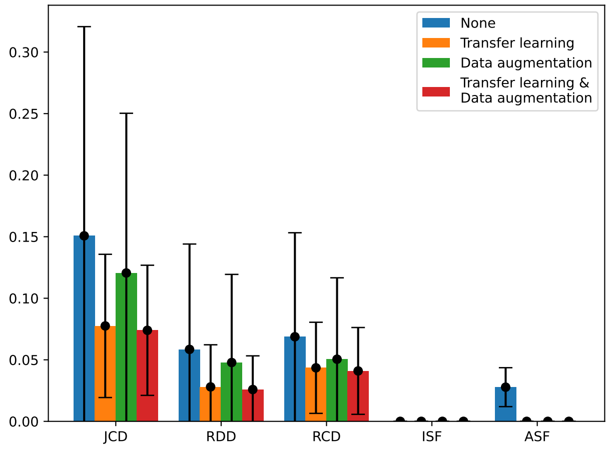

Extension	JCD	RDD	RCD	ISF	ASF
None	0.151	0.058	0.069	**0.000**	0.028
±	0.170	0.086	0.084	0.000	0.016
Transfer learning	0.078	0.028	0.043	**0.000**	**0.000**
±	0.058	0.034	0.037	0.000	0.000
Data augmentation	0.121	0.048	0.050	**0.000**	**0.000**
±	0.130	0.072	0.066	0.000	0.000
Transfer learning &	**0.074**	**0.026**	**0.041**	**0.000**	**0.000**
Data augmentation ±	0.053	0.027	0.035	0.000	0.000

Figure SM.7. Values of evaluation metrics for different extensions of the training data set, which is used by the U-Net. Bold values highlight the optimum in each column. The other hyperparameters are fixed at Backbone: ResNet 34, Optimizer: Adam, Loss: Cross-Entropy, Resize factor: 1/2. The accuracy of the U-Net is highest when transfer learning and data augmentation is used.

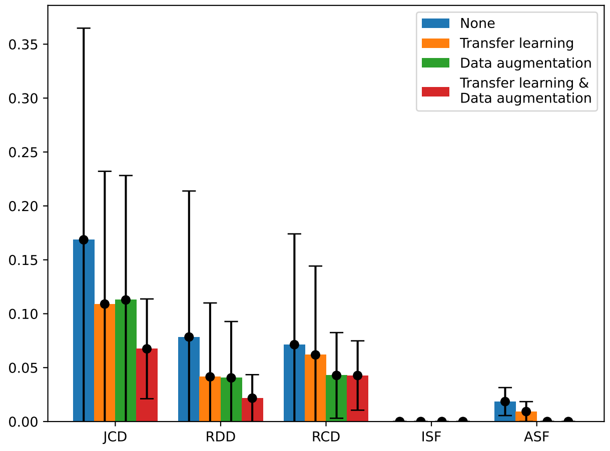

Extension	JCD	RDD	RCD	ISF	ASF
None	0.169	0.078	0.071	**0.000**	0.019
±	0.196	0.135	0.103	0.000	0.013
Transfer learning	0.109	0.041	0.062	**0.000**	0.009
±	0.123	0.069	0.082	0.000	0.009
Data augmentation	0.113	0.040	**0.043**	**0.000**	**0.000**
±	0.115	0.052	0.040	0.000	0.000
Transfer learning &	**0.067**	**0.022**	**0.043**	**0.000**	**0.000**
Data augmentation ±	0.046	0.022	0.032	0.000	0.000

Figure SM.8. Values of evaluation metrics for different extensions of the training data set, which is used by the HRNet. Bold values highlight the optimum in each column. The other hyperparameters are fixed at Backbone: W48, Optimizer: Adam, Loss: Cross-Entropy, Resize factor: 1/2. The accuracy of the HRNet is highest when transfer learning and data augmentation is used.

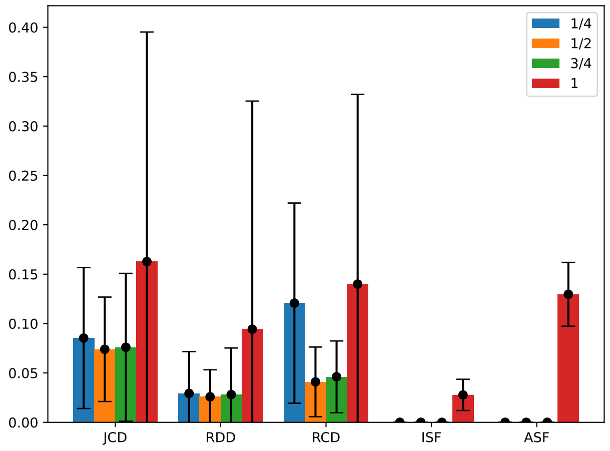

Resize factor	JCD	RDD	RCD	ISF	ASF
1/4	0.085	0.029	0.121	**0.000**	**0.000**
±	0.071	0.042	0.101	0.000	0.000
1/2	**0.074**	**0.026**	**0.041**	**0.000**	**0.000**
±	0.053	0.027	0.035	0.000	0.000
3/4	0.076	0.028	0.046	**0.000**	**0.000**
±	0.075	0.047	0.036	0.000	0.000
1	0.163	0.094	0.140	0.028	0.130
±	0.232	0.231	0.192	0.016	0.032

Figure SM.9. Values of evaluation metrics for different image sizes. Bold values highlight the optimum in each column. The other hyperparameters are fixed at Backbone: ResNet 34, Optimizer: Adam, Loss: Cross-Entropy, Transfer learning: Yes, Data augmentation: Yes. The U-Net can achieve the highest accuracy when the original image size is reduced by half.

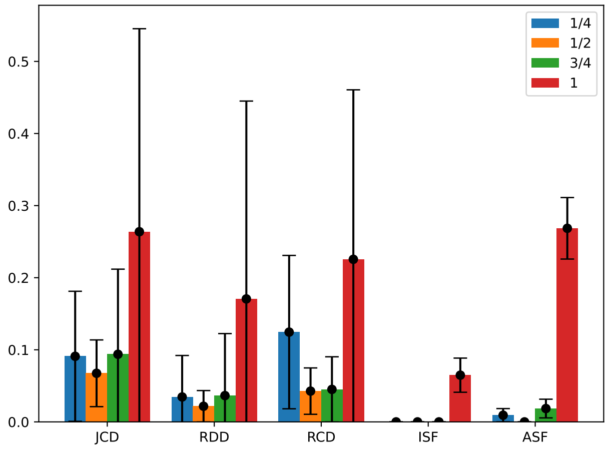

Resize factor	JCD	RDD	RCD	ISF	ASF
1/4	0.091	0.035	0.125	**0.000**	0.009
±	0.090	0.058	0.106	0.000	0.009
1/2	**0.067**	**0.022**	**0.043**	**0.000**	**0.000**
±	0.046	0.022	0.032	0.000	0.000
3/4	0.094	0.037	0.045	**0.000**	0.019
±	0.118	0.086	0.045	0.000	0.013
1	0.264	0.171	0.226	0.065	0.269
±	0.282	0.274	0.235	0.024	0.043

Figure SM.10. Values of evaluation metrics for different image sizes. Bold values highlight the optimum in each column. The other hyperparameters are fixed at Backbone: W48, Optimizer: Adam, Loss: Cross-Entropy, Transfer learning: Yes, Data augmentation: Yes. The HRNet can achieve the highest accuracy when the original image size is reduced by half.

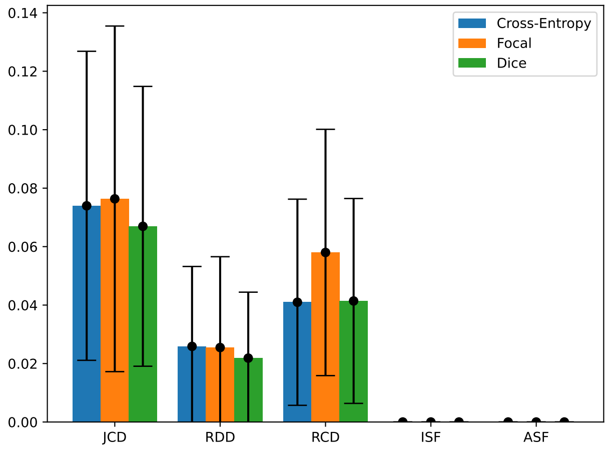

Loss function	JCD	RDD	RCD	ISF	ASF
Cross-Entropy	0.074	0.026	0.041	**0.000**	**0.000**
±	0.053	0.027	0.035	0.000	0.000
Focal	0.076	0.025	0.058	**0.000**	**0.000**
±	0.059	0.031	0.042	0.000	0.000
Dice	**0.067**	**0.022**	**0.041**	**0.000**	**0.000**
±	0.048	0.023	0.035	0.000	0.000

Figure SM.11. Values of evaluation metrics for different loss functions, used by the U-Net. Bold values highlight the optimum in each column. The other hyperparameters are fixed at Backbone: ResNet 34, Optimizer: Adam, Resize factor: 1/2, Transfer learning: Yes, Data augmentation: Yes. The U-Net can achieve the highest accuracy when the Dice loss is used.

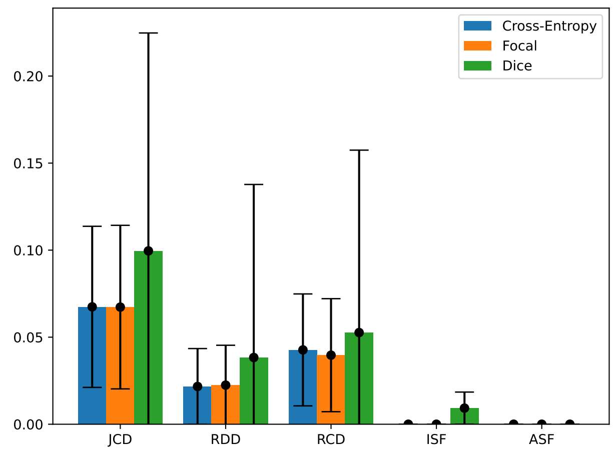

Loss function	JCD	RDD	RCD	ISF	ASF
Cross-Entropy	**0.067**	**0.022**	0.043	**0.000**	**0.000**
±	0.046	0.022	0.032	0.000	0.000
Focal	**0.067**	**0.022**	**0.040**	**0.000**	**0.000**
±	0.047	0.023	0.032	0.000	0.000
Dice	0.100	0.038	0.053	0.009	**0.000**
±	0.125	0.099	0.105	0.009	0.000

Figure SM.12. Values of evaluation metrics for different loss functions, used by the HRNet. Bold values highlight the optimum in each column. The other hyperparameters are fixed at Backbone: W48, Optimizer: Adam, Resize factor: 1/2, Transfer learning: Yes, Data augmentation: Yes. The HRNet can achieve the highest accuracy, if the Cross-Entropy or the Focal loss is used.

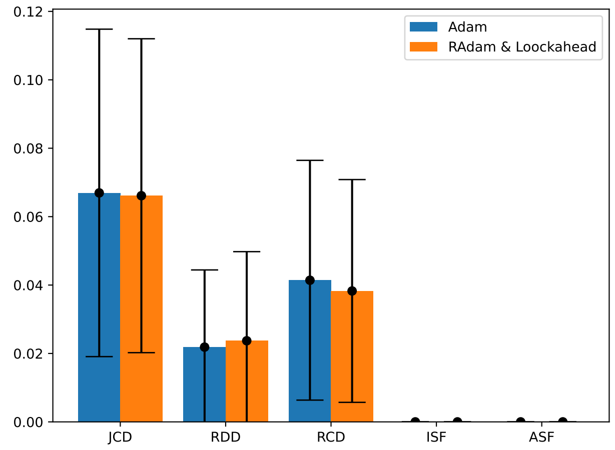

Optimizer function	JCD	RDD	RCD	ISF	ASF
Adam	0.067	**0.022**	0.041	**0.000**	**0.000**
±	0.048	0.023	0.035	0.000	0.000
RAdam & Lookahead	**0.066**	0.024	**0.038**	**0.000**	**0.000**
±	0.046	0.026	0.033	0.000	0.000

Figure SM.13. Values of evaluation metrics for different optimizers, used by the U-Net. Bold values highlight the optimum in each column. The other hyperparameters are fixed at Backbone: ResNet 34, Loss: Dice, Resize factor: 1/2, Transfer learning: Yes, Data augmentation: Yes. The U-Net can achieve the highest accuracy when the optimization is done by RAdam combined with Lookahead.

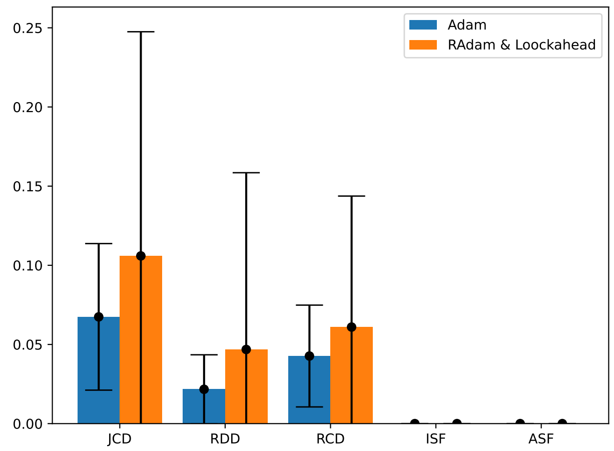

Optimizer function	JCD	RDD	RCD	ISF	ASF
Adam	**0.067**	**0.022**	**0.043**	**0.000**	**0.000**
±	0.046	0.022	0.032	0.000	0.000
RAdam & Lookahead	0.106	0.047	0.061	**0.000**	**0.000**
±	0.142	0.112	0.083	0.000	0.000

Figure SM.14. Values of evaluation metrics for different optimizers, used by the HRNet. Bold values highlight the optimum in each column. The other hyperparameters are fixed at Backbone: W48, Loss: Cross-Entropy, Resize factor: 1/2, Transfer learning: Yes, Data augmentation: Yes. The HRNet can achieve the highest accuracy when the optimization is done by Adam.

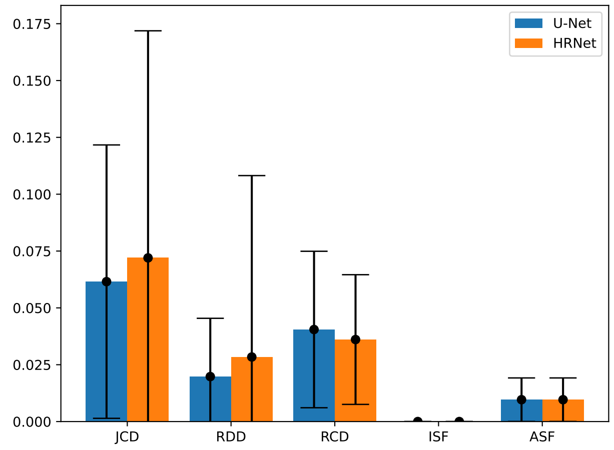

Model	JCD	RDD	RCD	ISF	ASF
U-Net	**0.062**	**0.020**	0.040	**0.000**	**0.010**
±	0.060	0.026	0.034	0.000	0.010
HRNet	0.072	0.028	**0.036**	**0.000**	**0.010**
±	0.100	0.080	0.029	0.000	0.010

Figure SM.15. Evaluation of the segmentation with the optimized U-Net and HRNet models on the test data set shows higher accuracy of the U-Net. The optimal hyperparameter configuration for the U-Net is Backbone: ResNet 34, Optimizer: RAdam & Lookahead, Loss: Dice, Resize factor: 1/2, Transfer learning: Yes, Data augmentation: Yes. The final hyperparameter configuration for the HRNet is Backbone: W48, Optimizer: Adam, Loss: Cross-Entropy, Resize factor: 1/2, Transfer learning: Yes, Data augmentation: Yes.

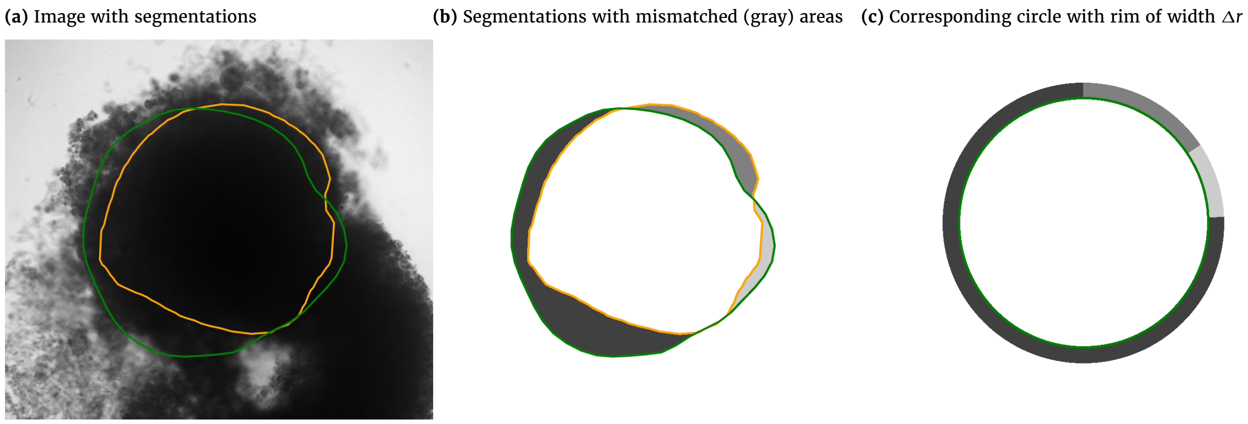

Figure SM.16. Illustration of the average radial error Δr defined in Eq. (3) (example with large JCD $>$ 0.2 for visibility). (a) Zoom of image from Fig. SM.4 with JCD = 0.24 with corresponding automatic (orange line containing predicted area P) and manual segmentation (green line containing target area T). (b) Segmentations from (a) with mismatched areas shown in gray (missing area $T \setminus P$ in bright and dark gray, additional area $P \setminus T$ in intermediate gray). (c) Circle (green line) and added rim (gray areas) with areas corresponding to (b): Area within green circle is equal to area within manual segmentation in (b) and gray areas within the adjacent rim are equal to corresponding mismatched areas in (b). Then the radial thickness of the rim is equal to the average radial error Δr defined in Eq. (3).

(a) RDD over spheroid diameter

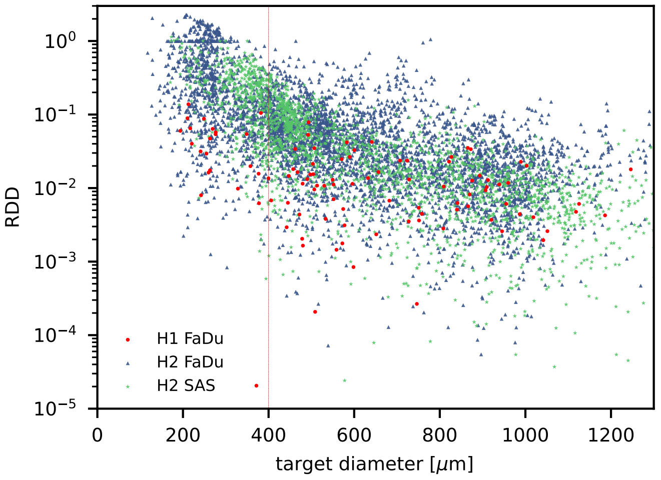

(b) Comparison diameters from manual and automatic segmentation

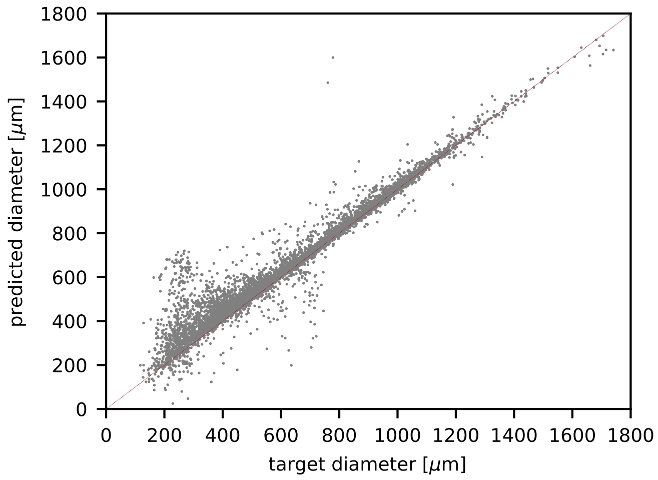

Figure SM.17. Validation based on diameter of automatic segmentation with the optimized U-Net on larger, independent data sets, analogous to Fig. 4. (a) RDD shows that majority of deviations are below 10% and higher deviations occur mostly below $d_T < 400$ µm. (b) Direct comparison of diameters (gray points) resulting from automatic segmentation (predicted diameter) and manual segmentation (target diameter) shows high accuracy (red line represents perfect match) with larger deviations mostly at $d_T < 400$ µm.
