# Supplementary material for: Image segmentation of treated and untreated tumor spheroids by fully convolutional networks
Source: Gigascience. 2025 May 7;14:giaf027. doi: 10.1093/gigascience/giaf027 (PMC12056507; doi:10.1093/gigascience/giaf027)
Supplement: giaf027_GIGA-D-24-00570_Revision_1 [file giaf027_giga-d-24-00570_revision_1.pdf]

GigaScience

Image segmentation of treated and untreated tumor spheroids by Fully Convolutional Networks

--Manuscript Draft--

**Manuscript Number:**	GIGA-D-24-00570R1
**Full Title:**	Image segmentation of treated and untreated tumor spheroids by Fully Convolutional Networks
**Article Type:**	Technical Note
**Funding Information:**	Europäischer Sozialfonds (SAB-Nr. 100382145) — Prof. Dr. Anja Voss-Böhme<br>Bundesministerium für Bildung und Forschung (16dkwn001a) — Prof. Dr. Leoni A. Kunz-Schughart<br>Bundesministerium für Bildung und Forschung (16dkwn001b) — Prof. Dr. Anja Voss-Böhme
**Abstract:**	Background Multicellular tumor spheroids (MCTS) are advanced cell culture systems for assessing the impact of combinatorial radio(chemo)therapy as they exhibit therapeutically relevant in-vivo-like characteristics from 3D cell-cell and cell-matrix interactions to radial pathophysiological gradients. State-of-the-art assays quantify long-term curative endpoints based on collected brightfield image time series from large treated spheroid populations per irradiation dose and treatment arm. This analyses require laborious spheroid segmentation of up to 100.000 images per treatment arm to extract relevant structural information from the images, e.g., diameter, area, volume and circularity. While several image analysis algorithms are available for spheroid segmentation, they all focus on compact MCTS with clearly distinguishable outer rim throughout growth. However, they often fail for the common case of treated MCTS, which may partly be detached and destroyed and are usually obscured by dead cell debris.<br>Results To address these issues, we successfully train two Fully Convolutional Networks, UNet and HRNet, and optimize their hyperparameters to develop an automatic segmentation for both untreated and treated MCTS. We extensively test the automatic segmentation on larger, independent data sets and observe high accuracy for most images with Jaccard indices around 90%. For cases with lower accuracy, we demonstrate that the deviation is comparable to the inter-observer variability. We also test against previously published datasets and spheroid segmentations.<br>Conclusions The developed automatic segmentation cannot only be used directly but also integrated into existing spheroid analysis pipelines and tools. This facilitates the analysis of 3D spheroid assay experiments and contributes to the reproducibility and standardization of this preclinical in-vitro model.
**Corresponding Author:**	Steffen Lange<br>TU Dresden: Technische Universitat Dresden<br>Dresden, GERMANY
**Corresponding Author Secondary Information:**	
**Corresponding Author's Institution:**	TU Dresden: Technische Universitat Dresden
**Corresponding Author's Secondary Institution:**	
**First Author:**	Matthias Streller
**First Author Secondary Information:**	
**Order of Authors:**	Matthias Streller<br>Soňa Michl'íková<br>Willy Ciecior

Powered by Editorial Manager® and ProduXion Manager® from Aries Systems Corporation

	Katharina Lönnecke
	Leoni A. Kunz-Schughart
	Steffen Lange
	Anja Voss-Böhme
**Order of Authors Secondary Information:**	
**Response to Reviewers:**	We detailed our revisions and reply to reviewers in attached review_reply_giga.pdf. We also attached a compiled version of the revised manuscript with changes highlighted (diff.pdf)
**Additional Information:**	
**Question**	**Response**
Are you submitting this manuscript to a special series or article collection?	No
**Experimental design and statistics**<br><br>Full details of the experimental design and statistical methods used should be given in the Methods section, as detailed in our Minimum Standards Reporting Checklist. Information essential to interpreting the data presented should be made available in the figure legends.<br><br>Have you included all the information requested in your manuscript?	Yes
**Resources**<br><br>A description of all resources used, including antibodies, cell lines, animals and software tools, with enough information to allow them to be uniquely identified, should be included in the Methods section. Authors are strongly encouraged to cite Research Resource Identifiers (RRIDs) for antibodies, model organisms and tools, where possible.<br><br>Have you included the information requested as detailed in our Minimum Standards Reporting Checklist?	No
If not, please give reasons for any omissions below.	The experimental data used in our study has been previously published and we refer for details to the original publication.

as follow-up to "**Resources**<br><br>A description of all resources used, including antibodies, cell lines, animals and software tools, with enough information to allow them to be uniquely identified, should be included in the Methods section. Authors are strongly encouraged to cite Research Resource Identifiers (RRIDs) for antibodies, model organisms and tools, where possible.<br><br>Have you included the information requested as detailed in our Minimum Standards Reporting Checklist?<br><br>"	However, the annotated image data, computational methods and software is provided open source and all necessary information is provided.
**Availability of data and materials**<br><br>All datasets and code on which the conclusions of the paper rely must be either included in your submission or deposited in publicly available repositories (where available and ethically appropriate), referencing such data using a unique identifier in the references and in the “Availability of Data and Materials” section of your manuscript.<br><br>Have you have met the above requirement as detailed in our Minimum Standards Reporting Checklist?	Yes
GigaScience has policies and guidelines in place for the use of generative AI-writing tools such as ChatGPT. If you have used such writing tools to assist with writing the manuscript this must be declared and cited in the text. Authors should not list AI-writing tools and other AI-assisted technologies as an author or co-author and should acknowledge that they are fully responsible for text generated or refined by AI-writing tools.<p><br><br>A summary of use (particularly in the introduction or among methods) needs to be included at the end of the paper, and	No

the outputs should also be included as a supplementary file hosted in GigaDB or other open repositories. Please read our guidelines for more information. <p><br><br>By submitting to GigaScience, you are aware of the journal's AI-writing tools policy, and if you have declared use of such tools below, you have acknowledged this where appropriate in your manuscript and have made a summary of use and outputs available. <p><br><b>AI-assisted writing tools have been used in the preparation of this manuscript?	

LaTeX - Main Document (TeX file) Click here to access/download;LaTeX - Main Document (TeX file);paper_giga.tex

GigaScience, 2024, 1–21

doi: xx.xxxx/xxxx
Manuscript in Preparation
Tech Note

TECH NOTE

Image segmentation of treated and untreated tumor spheroids by Fully Convolutional Networks

Matthias Streller[1,2], Soňa Michlíková[3,4], Willy Cieciór[1,2], Katharina Lönnecke[1,2], Leoni A. Kunz-Schughart[4,5], Steffen Lange[4,1,†,*] and Anja Voss-Böhme[1,2,*]

[1]DataMedAssist Group, HTW Dresden-University of Applied Sciences, 01069 Dresden, Germany
[2]Faculty of Informatics/Mathematics, HTW Dresden-University of Applied Sciences, 01069 Dresden, Germany
[3]Helmholtz-Zentrum Dresden-Rossendorf, Institute of Radiooncology - OncoRay, Dresden, Germany
[4]OncoRay - National Center for Radiation Research in Oncology, Faculty of Medicine and University Hospital Carl Gustav Carus, TUD Dresden University of Technology, Helmholtz-Zentrum Dresden-Rossendorf, Dresden, Germany
[5]National Center for Tumor Diseases Dresden (NCT/UCC), Germany: German Cancer Research Center (DKFZ), Heidelberg, Germany; Faculty of Medicine and University Hospital Carl Gustav Carus, TUD Dresden University of Technology, Dresden, Germany; Helmholtz-Zentrum Dresden-Rossendorf (HZDR), Dresden, Germany

[†]steffen.lange@tu-dresden.de
[*]These authors contributed equally to this work and share last authorship.

Abstract

Background Multicellular tumor spheroids (MCTS) are advanced cell culture systems for assessing the impact of combinatorial radio(chemo)therapy as they exhibit therapeutically relevant in-vivo-like characteristics from 3D cell-cell and cell-matrix interactions to radial pathophysiological gradients. State-of-the-art assays quantify long-term curative endpoints based on collected brightfield image time series from large treated spheroid populations per irradiation dose and treatment arm. This analyses require laborious spheroid segmentation of up to 100.000 images per treatment arm to extract relevant structural information from the images, e.g., diameter, area, volume and circularity. While several image analysis algorithms are available for spheroid segmentation, they all focus on compact MCTS with clearly distinguishable outer rim throughout growth. However, they often fail for the common case of treated MCTS, which may partly be detached and destroyed and are usually obscured by dead cell debris.
Results To address these issues, we successfully train two Fully Convolutional Networks, UNet and HRNet, and optimize their hyperparameters to develop an automatic segmentation for both untreated and treated MCTS. We extensively test the automatic segmentation on larger, independent data sets and observe high accuracy for most images with Jaccard indices around 90%. For cases with lower accuracy, we demonstrate that the deviation is comparable to the inter-observer variability. We also test against previously published datasets and spheroid segmentations.
Conclusions The developed automatic segmentation cannot only be used directly but also integrated into existing spheroid analysis pipelines and tools. This facilitates the analysis of 3D spheroid assay experiments and contributes to the reproducibility and standardization of this preclinical *in-vitro* model.

Key words: Spheroids, Organoids, Brightfield Microscopy, Segmentation, Deep Learning, Fully Convolutional Networks, High-content Screening, 3D Cancer Models, Cancer Therapy, Inter-observer Variability

Introduction

One of the most challenging problems in oncology is the development of therapeutic approaches for tumor growth suppression and eradication, as well as designing and optimizing treatment protocols. In this context, three-dimensional (3D) multicellular tumor spheroids (MCTS) are an advocated pre-clinical, in-vitro model to systematically study possible means of tumor suppression, assess the curative effect of combinatorial radio(chemo)therapy, and predict the response of *in-vivo* tumors [1–8]. In contrast to two-dimensional (2D) clonogenic survival assays, which are known to reflect the therapeutic response of cancer cells in the tissue comparatively poorly [9], multicellular spheroids are reproducible 3D avascular clusters of several thousand tumor cells without or in advanced settings with stromal cell compartments mimicking the pathophysiological milieu of tumor microareas or micrometastases. Due to their more or less radial 3D histomorphology and structure, they exhibit many characteristic features affecting tumor growth dynamics, including 3D reciprocal cell-cell and cell-extracellular-

Compiled on: February 7, 2025.
Draft manuscript prepared by the author.

Key Points

- 3D tumor spheroids as advocated pre-clinical model lack reliable image segmentation.
- An automatic spheroid segmentation even for treated spheroids obscured by dead cell debris is developed.
- Segmentation is tested against independent datasets and compared to previous deep learning models.
- High-quality training dataset with challenging cases of treated spheroids is provided.
- Complementary software tool is released for high throughput image analysis of spheroids.

matrix interactions as well as metabolic gradients of oxygen, nutrients and waste products, which strongly impair the cells' proliferative activity and therapy response [4–8], in particular oxygen deficiency (hypoxia), which is associated with substantial radioresistance [10–13]. After a long phase in which spheroids were used only in specialized laboratories, methodological advances in serial culturing and live imaging have led to an exponential spread of this in-vitro model system over the past two decades [7, 8, 14].

While 3D tumor spheroids provide a physiologically more realistic *in-vitro* framework to study tumor growth dynamics and therapeutic outcomes, the analysis of the experiments is much more complex than for traditional 2D cultures. 3D tumor spheroids' dynamics with and without treatment are most frequently monitored via microscopy imaging. State-of-the-art long-term spheroid-based assays assess therapy response from these image time series by classifying each spheroid within a population as either controlled or relapsed based on their growth kinetics. By averaging the therapeutic response over ensembles of spheroids for each treatment dose, the spheroid control probabilities and spheroid control doses are computed as analytical endpoints [15–18], analogous to the tumor control dose employed in *in-vivo* radiotherapy experiments with tumor-bearing mice [19, 20]. Moreover, time points of relapse are identified to quantify growth recovery in terms of Kaplan-Meier curves. These metrics depend on the spheroid type (cell line), the size of the spheroids at the onset of treatment, and the applied treatment and often require the extraction of growth curves, i.e., spheroid volume over time, from the image time series of all individual spheroids.

The associated data analysis poses a significant, interdisciplinary challenge: A single, typical experimental series of a long-term spheroid-based assay, i.e., 2-3 cell models at ten different doses with 3-5 agents and 30 spheroids per treatment arm monitored up to 60 days after treatment, generates up to 10^5 images. In the past, several tools were developed for spheroid image analysis [21–26], including SpheroidSizer [27], AnaSP [28, 29], TASI [30], SpheroidJ [31], and INSIDIA [25, 32]. The most crucial part of spheroid image analysis is the identification of the set of pixels in an image that corresponds to the spheroid, as this forms the basis for the extraction of spheroid characteristics like diameter, volume, and circularity as well as other morphologic features. This identification or classification of pixels in a given image is denoted semantic image segmentation [33] and represents the greatest challenge in spheroid image analysis. Previous spheroid segmentations were based on classical segmentation techniques, including thresholding methods (Yen [34], Otsu [28, 35]), watershed algorithm [36], shape-based detections (Hough transform algorithms [37], active contour models/snake [38]), or edge detection (Canny, Sobel [31]), and are also available as ImageJ plugins [22, 25], Matlab packages [27, 28, 30], or dedicated segmentation programs [39–41]. Since the characteristics of spheroid images, including the size, shape, and texture of the actual spheroid, vary with cell line, treatment, and microscopy method (e.g., brightfield [25, 29, 31, 32, 42], fluorescence [25, 26, 31, 32, 43, 44], differential, interference contrast [45]), most of these approaches and tools are specialized for specific experimental conditions or even selected microscopes [43, 44] and often fail to generalize to arbitrary conditions. In recent years, data-based methods, especially deep-learning models, have been increasingly employed for spheroid segmentation to tackle this issue of generalization [29, 31, 42, 45–49]. Deep-learning segmentation also allows more complex analysis, e.g., identification of time-lapse migratory patterns [45] or distinction of the spheroids' core and invasive edge [32].

However, these segmentation and analysis tools have primarily been developed for experimental conditions where the resulting images are relatively clean with well-visible, unobscured MCTS. In contrast, radiotherapy, one of the most common cancer treatments, regularly causes spheroids to shed dead cells or even to detach completely. Consequently, dead cell debris often obscures the remaining shrunk MCTS and the 3D cultures putatively regrowing from surviving viable cells after detachment, see Fig. 1 for two examples. This debris can cover a much larger domain than the actual spheroid and can be locally even thicker and thus darker, which makes segmentation difficult even for human experts. Both classical segmentation techniques and previous deep-learning models typically fail in these cases and require manual adjustments. We illustrate this challenge of spheroid segmentation by applying the four most recently proposed deep-learning models [29, 31, 48] to images with and without severe cell debris, see Fig. SM.1 for representative examples and Tab. SM.1 for statistical results.

We train deep-learning models to segment spheroid images after radiotherapy based on annotated images from a previous experiment assessing the radioresponse of human head-and-neck squamous cell carcinoma (HNSCC). After systematic optimization of hyperparameters and pre-processing for two selected network architectures, U-Net and HRNet, the automatic segmentation exhibits high accuracy for images of both treated and untreated spheroids. We further validate the automatic segmentation with the optimized U-Net on larger, independent data sets of two cell types of head-and-neck cancer exposed to both radiotherapy and hyperthermia. For the majority of images, we find excellent overlap between manual and automatic segmentation, even in the case of small, heavily obscured spheroids, see Fig. 1. In particular, we compare segmentations of different biological experts and find that imprecisions of the automatic segmentation are comparable to variations across different humans. The optimized automatic segmentation is provided in a minimal tool with a graphical user interface.

Materials

The original data is compiled from several data sets of time images of FaDu cell line spheroids, one of the two head-and-neck cancer cell types from the previously published study on combinatorial radioresponse in human head-and-neck squamous cell carcinoma spheroid models [16]: Spheroid populations were irradiated with X-rays at single doses of either 2.5 Gy, 5.0 Gy, 7.5 Gy or 10.0 Gy, with each dose being represented approximately equally in the data set. Spheroids were imaged for up to 60 days after treatment with brightfield microscopy, i.e., at different times after treatment and with potentially different lighting conditions. 1095 spheroids were manually segmented by a biological expert (human H1) to train and test the fully convolutional networks (FCNs). We use the

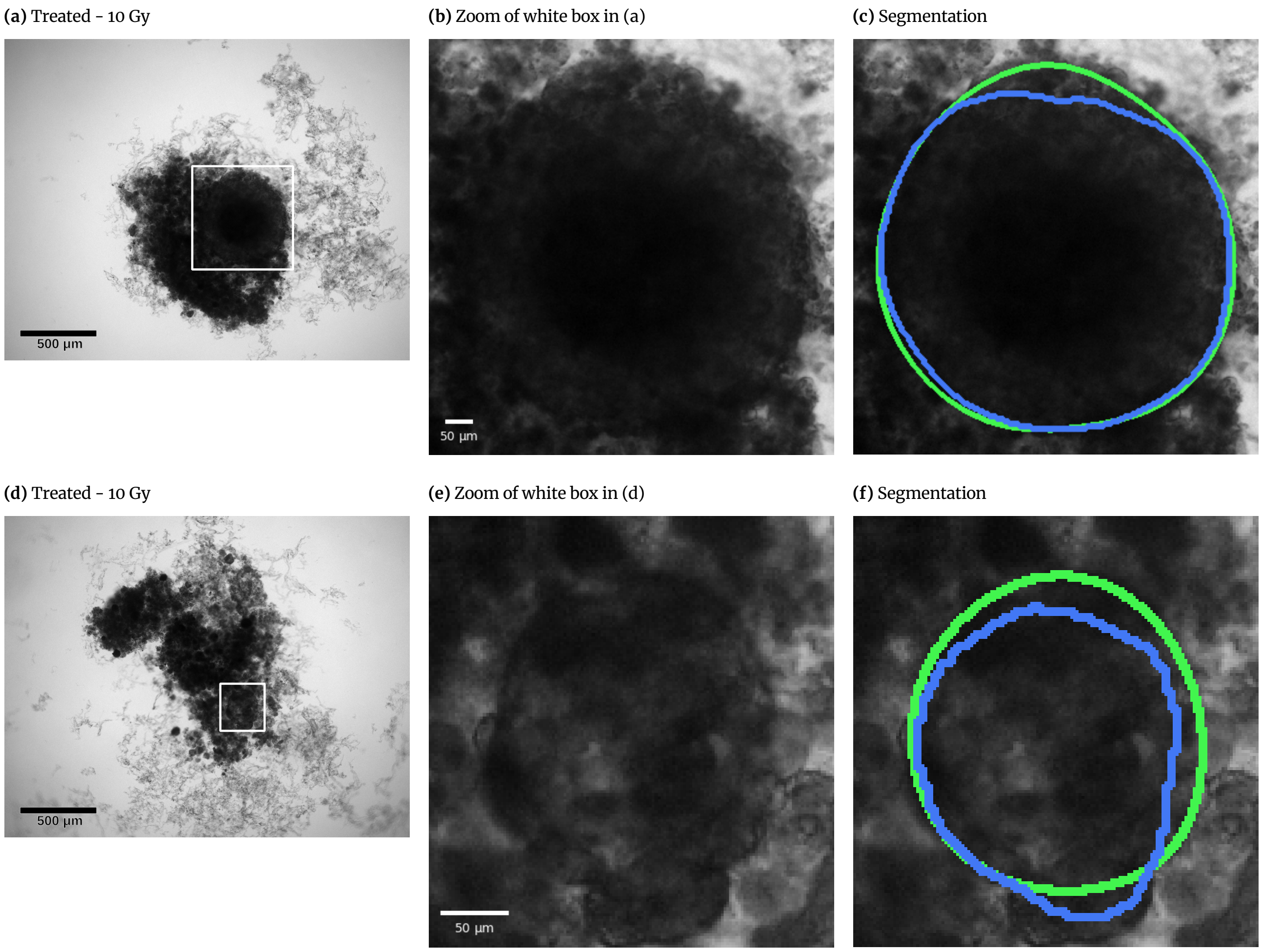

Figure 1. Representative examples of images for automatic segmentation with the optimized U-Net (blue) compared to the manual segmentation (green) for 3D tumor spheroids after treatment. The overlap with the manual segmentation is excellent for standard size and larger spheroids obscured by cell debris (top row) and sufficient for small, heavily obscured spheroids (bottom row). Displayed are (a,d) the original images, which are also the input for the U-Net, (b,d) magnified image details around the spheroids as indicated by the white box in (a,d) for visibility, and (c,e) corresponding contours from the segmentations. The metrics for evaluation of the automatic segmentation are: the top row - Jaccard Distance (JCD) = 7.6%, Relative Diameter Deviation (RDD) = 3.7% and Relative Circularity Deviation (RCD) = 1.6%; bottom row - JCD = 22.7%, RDD = 7.5% and RCD = 14.7%, see text for details.

train-validation-test-split technique for validation during training, which is appropriate for the large datasets and commonly used to train spheroid segmentation models [29, 31, 48]. We split the annotated data into 3 separate data sets: 883 images for training, 108 for validation, and 104 for testing (Hold-out test set). Images from a single spheroid are exclusively assigned to one of the groups to rule out unwanted correlations and data leakage between training, validation, and testing data sets. For further testing, another set of 6574 images of head-and-neck cancer (FaDu and SAS) spheroids from the same study [16], treated with different combinations and doses of X-ray irradiation and hyperthermia, is manually segmented by several independent biological experts (humans H2-H5). Note that while the automatic segmentation is performed on individual images, biological experts always take precedent and subsequent images of a spheroid into account for manual segmentation.

The original images were taken with a Zeiss Axiovert 200M at a resolution of 1300 × 1030 pixels, representing 2.04 µm/pixel with 16-bit gray-scale per pixel [16]. Images are converted to 8-bit images to ensure compatibility with the employed libraries FastAi [50] and SemTorch [51]. While this conversion, in principle, reduces the contrast level, the effect is negligible as only a small fraction of the 16-bit range is utilized during imaging, e.g., the mean gray-scale value in the images is 1300 ± 130, and values are rescaled such that smallest gray-scale value of the original image becomes 0 and the biggest value becomes 255.

As deep-learning models, we use FCNs, a particular group of convolutional neural networks intended for semantic image segmentation [52], in particular the U-Net [53] and the HRNet [54]. The U-Net is one of the most established FCN frameworks. The HRNet has a special architecture compared to other typical FCN models and has already been successfully employed in the case of clean images with clearly visible spheroids [31]. The pipeline to train the models employs FastAi [50] for the U-Net and its backbones and SemTorch [51] for the HRNet. Before each training, a suitable learning rate is picked using LR Range [55]. The training follows the 1-cycle policy: the learning rate is cycled once between two bounds, allowing fast convergence [55]. During training we use validation-based early stopping, where the JCD is the underlying metric. The models are trained on a GPU NVIDIA GeForce RTX 3080 with a mini-batch size of 2. For training on images with higher resolution or to compare some specific backbones, online learning is used instead of mini-batches to avoid memory overflow, i.e., the batch size, the amount of data used in each sub-epoch weight change, is reduced to one.

Methods

The segmentation is evaluated via several metrics (Section Evaluation metrics: Jaccard Distance (JCD), Relative Diameter Deviation (RDD), Relative Circularity Deviation (RCD), Ambiguous Spheroid Fraction (ASF), Invalid Spheroid Fraction (ISF)) additional to the standard accuracy quantified by the Jaccard coefficient of the whole data set, which is used during training. These metrics are computed for individual images to report not only their mean values but also their deviations, and thus reliability, across the test data set. These metrics are used to optimize the hyperparameters, Section Hyperparameter optimization , and data augmentation, Section Data Augmentation, of the selected FCN models U-Net and HRNet for maximal accuracy.

Evaluation metrics

Several metrics are employed to asses the accuracy of the segmentation. The most important one is the Jaccard Distance (JCD), which measures the relative difference between two sets of pixel P and T,

$$\mathrm{JCD} = 1 - \mathrm{IoU} = 1 - \frac{|P \cap T|}{|P \cup T|}. \tag{1}$$

with the automatically segmented (predicted) pixel set P from the FCN and the (target) pixel set T of the manually segmented spheroid. Note that in the literature often the opposite metric Intersection over Union IoU $= 1-$JCD also called Jaccard index, or Jaccard similarity coefficient, is used. The JCD takes values between 0 (automatic segmentation perfectly overlaps with the manual one) and 1 (no intersection between the two segmentations). The JCD can be understood as the relative area error of the segmentation, i.e., in the example of Fig. 2a JCD $= 0.042$ is obtained, which means 4.2% error in the segmented area. A selection of sample images with different JCDs is displayed in Figs. SM.2-SM.4 to give an optical reference for this metric. From these sample images and in accordance with the biological experts, it may be concluded that a JCD below 0.2 is justifiable and such a deviation is observed between segmentations of different humans, see also Section Further independent validation .

It is possible that the spheroid is correctly segmented, but additionally another non-existent spheroid is detected, see Fig. 2b, where additionally to the manually segmented spheroid in the top right another contour in the bottom left is suggested by the automatic segmentation. In this case, the JCD would be below 1 but differ from 0. The fraction of these cases among all images are additionally denoted Ambiguous Spheroid Fraction (ASF). Furthermore, a JCD of 1 can mean that the spheroid is found in the wrong place or the spheroid is not found at all, see Fig. 2c. The fraction of these cases is denoted Invalid Spheroid Fraction (ISF).

In practice, only the average diameter $d_T = 2\sqrt{|T|/\pi}$ of the segmented spheroid is extracted to estimate the three-dimensional volume $\pi d_T^3/6$ of the spheroid under the assumption of a spherical shape. Additionally, the circularity $4\pi|T|/L^2$ of the spheroid is computed from its area $|T|$ and perimeter L to assess the validity of this assumption. Note that this is just a common assumption in the field, although even a circular projection does not imply a spherical shape. Thus, we also assess the automatic segmentation by the resulting error of these two values and measure the Relative Diameter Deviation (RDD) and Relative Circularity Deviation (RCD) between the segmentations

$$\mathrm{RDD}, \mathrm{RCD} = \frac{|c_P - c_T|}{c_T} \tag{2}$$

where c_P is either mean diameter or circularity based on the automatic segmentation and c_T is the corresponding characteristic calculated based on the manual segmentation. In the rare case that several spheroids are detected in an image, the largest one is chosen for computation of the JCD. This strategy is in accordance with experimental standard practice for single spheroid-based assays with curative analytical endpoints, as the largest regrowing spheroid is sufficient to detect relapse after treatment. Note that if there were several spheroids present and of interest, also all of them could be segmented. In the case of JCD $= 1$, which corresponds to an invalid spheroid, RDD and RCD are also set to 1. The combination of the JCD, ASF, ISF, RDD, and RCD quantify the accuracy of the segmentation with the FCN, where always a value closer to zero means higher agreement between the segmentations.

The spheroid's pixel set and perimeter are required to calculate the metrics. Thus, probability heatmaps, created by the Softmax function in the last layer of the FCN, are transformed into binary images and polygonal chains as illustrated in Fig. 3. First, the binary image is created. For this purpose, every pixel with a probability value higher or equal to 50% will be counted as a spheroid pixel and set to 1. Values of the other pixels are set to 0. The coordinates from the outer border of the emerged shape in the binary image can be extracted by the algorithm proposed in [56], thereby creating the final polygonal chain.

Hyperparameter optimization

Several hyperparameters and pre-processing methods can be adjusted to improve the accuracy of the selected FCN models. We

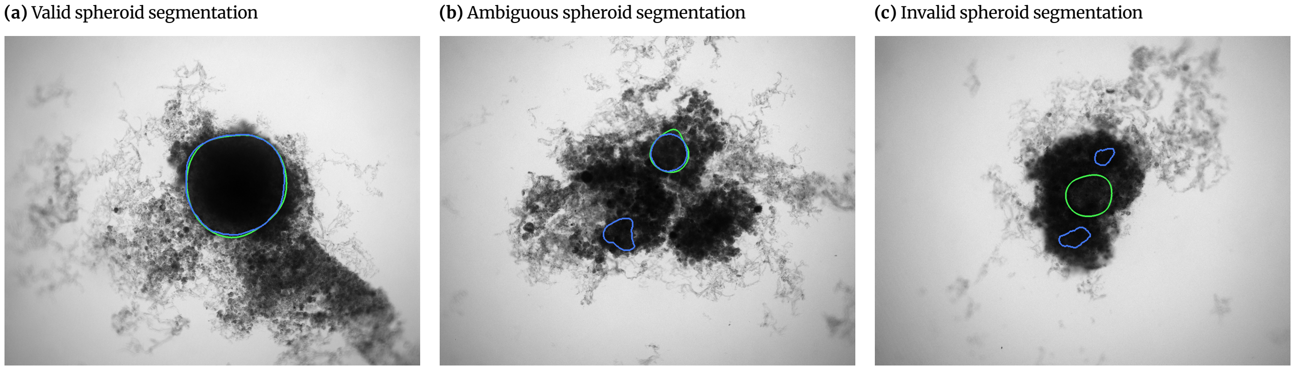

Figure 2. Exemplary evaluation of the automatic segmentation (blue) with respect to the manual one (green) for (a) standard case and (b),(c) rare artifacts, see text for details: (a) Correctly segmented spheroid: no contribution to Invalid Spheroid Fraction (ISF) or Ambiguous Spheroid Fraction (ASF); JCD, RDD and RCD are well defined. (b) Excessive spheroids are detected beyond the actual spheroid: no contribution to ISF, one count added to ASF; JCD, RDD and RCD are well defined and computed for the larger, upper spheroid. (c) No overlap between automatic and manual segmentation: one count added to ISF, no contribution to ASF; JCD, RDD and RCD are set to one.

successively optimize the most relevant parameters, starting from the ones with the most significant expected impact on accuracy to the one with the lowest impact, i.e., *backbone*, *transfer learning*, *data augmentation*, *input resolution*, *loss function*, and *optimizer*: The *backbone* defines the exact architecture of the FCN model and is accordingly the most important hyperparameter. For *transfer learning*, the ImageNet [57] data set is utilized. The *data augmentation* techniques are described in Section Data Augmentation. Furthermore, the impact of changing the *input resolution* is investigated. Afterward, the *loss function* for quantifying the FCN's error is optimized, particularly by comparing distribution-based loss and a region-based loss. We pick the Dice loss for region-based loss and the Cross-Entropy loss as a distribution-based loss function. We also test the Focal loss, which is a variation of Cross-Entropy and suitable for imbalanced class scenarios [33]. Finally, two different optimizers for minimizing the loss function are evaluated. One is Adam [58], a very common optimizer, and the other one is a combination of some more modern optimizers, RAdam [59] and Lookahead [60]. For the backbone, the optimization is initiated with a default setting, i.e., transfer learning, no data augmentation, input resolution half of original image, Cross-Entropy loss function, and Adam optimizer. These initial parameters are then individually optimized in consecutive order.

Data Augmentation

Data augmentation is a method to generate additional training data via transformations of the original data set. Since this increases the total amount of training data, data augmentation can improve performance and avoid overfitting [61]. In our case, every original training image is transformed once, doubling the size of the original training data set. For each image in the training data set, one of three transformations is picked randomly using Albumentations [61], i.e., vertical flip, horizontal flip, or rotation by 180°. These transformations are valid, since the spheroids do not have a specific orientation within an image. Note that each of these transformation conserves the original rectangular resolution of the image. This avoids extrapolating pixels on the border of the image, which would be necessary for arbitrary rotation angles and which can lead to additional image artifacts the FCN model would have to be trained for.

Results

Hyperparameter optimization

For each hyperparameter set and pre-processing method a model is trained and the performance of these trained models is compared for the validation data set based on the proposed evaluation metrics from Section Evaluation metrics. For JCD, RDD, and RCD not only the average value but also the standard deviation over the whole validation data set is reported to estimate reliability of the segmentation. For the ISF and the ASF the standard error of the mean of the corresponding binomial distribution is reported. All results of the optimization are listed in the supplemental material (Figs. SM.5-SM.14) and briefly discussed in the following.

Backbones
For the U-Net, the ResNet 34 and the VGG 19-bn architecture perform best, see Fig. SM.5, with a JCD three times smaller than the worst performing backbones, like AlexNet or SqueezeNet1.0. The two ResNet backbones stand out with a very low ASF. Note that the accuracy of the VGG architectures is improving with increasing number of used layers. The ResNet 34 is the optimal backbone as it not only performs best in virtually every metric, but also requires less computational time than the VGG 19-bn. Note that a previous study on deep-learning models for spheroid segmentation observed a higher performance for ResNet 18 compared to VGG 19 and ResNet 50 [29]. For the HRNet, the backbones differ in the number of kernels used and the accuracy increases with the number of kernels, see Fig. SM.6. The W48 backbone is preferred, as for the majority of metrics it performs best and has the lowest standard deviations.

Data augmentation and transfer learning
The combination of data augmentation and transfer learning leads to the best performance for both U-Net and HRNet, see Fig. SM.7 and Fig. SM.8. While this is in principle expected, the improvement is substantial as the JCD is more than halved when both transfer learning and data augmentation is introduced. Note that for the U-Net, transfer learning improves accuracy more than data augmentation, while the opposite is true for the HRNet.

Resolution
Using input images with half the resolution 650×515 of the original images leads to the best performance, see Fig. SM.9 and Fig. SM.10. This input resolution is already used to optimize the previous parameters. Higher resolution (3/4, 1) reduces accuracy, presumably because the increase in details makes generalization of the appearance of spheroids more difficult, and the receptive fields of the kernels are getting smaller relative to the whole image. Lower resolution (1/4) also reduces accuracy, presumably due to the loss of

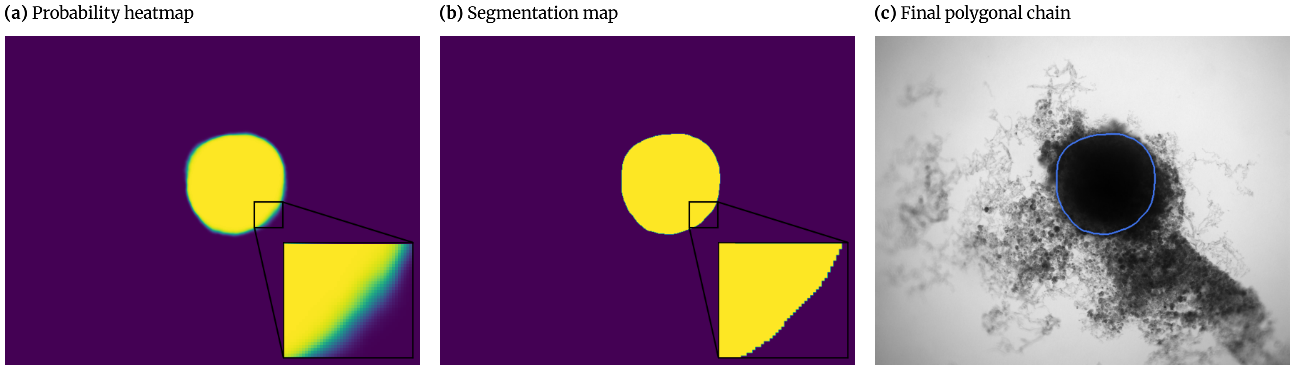

Figure 3. Post-processing steps for the output of the FCN model to transform probability heatmap to spheroid contour: (a) Probability heatmap as output of the FCN model. Each pixel takes probability values between 0 (black) and 1 (white) predicting the target (spheroid). Note that due to the steep gradient the gradual change from black to white is hard to see. (b) By using a threshold of 0.5, the pixels are classified into outside spheroid (black) and inside spheroid (white). (c) Contour of the spheroid border extracted as a polygonal chain (blue line displayed on original image).

relevant information. Half resolution displays the best accuracy and lowest standard deviations across the validation data set.

Loss

We find the Dice loss to be the optimal loss function for the U-Net, see Fig. SM.11. Note that Dice is considered the default loss parameter when using the U-Net for cell segmentation, i.e., binary classification concerned with the accuracy of the edge. However, the advantage is much smaller than for previous hyperparameters. It does not suggest a general advantage of region-based loss for the segmentation of spheroids by the U-Net. For the HRNet, there is a more significant difference between the region-based loss and the distribution-based loss, see Fig. SM.12. The Focal loss and the Cross-Entropy loss perform better than the Dice loss - especially, the standard deviations differ by a factor of up to three. One reason for this behavior may be that the Dice loss provides less detailed feedback during training than the distribution-based losses. Cross-Entropy loss and Focal loss achieve nearly the same results, and we choose the default Cross-Entropy loss.

Optimizer

In case of the U-Net the difference between the tested optimizers is marginal, see Fig. SM.13. We choose the slighly better performing RAdam combined with Lookahead over the default optimizer Adam. In contrast, the accuracy of the HRNet is much worse for the combined optimizer, see Fig. SM.14. The Adam optimizer is optimal with evaluation metrics 1.5 times better than for the RAdam combined with Lookahead.

Table 1. (a) Evaluation of the segmentation with the optimized U-Net and HRNet models on the test data set shows higher accuracy of the U-Net. Note that all metrics can range between zero and one, where lower values mean higher accuracy and zero means perfect agreement with the manual segmentation. (b) Evaluation of the optimized segmentations on images of untreated (not irradiated) spheroids shows high accuracy also for the case of clean images without cell debris. The optimized U-Net and HRNet achieve almost equal accuracy. (c) The optimal hyperparameter configurations.

(a) Segmentation test data set

Model	JCD	RDD	RCD	ISF	ASF
U-Net	**0.062**	**0.020**	0.040	**0.000**	**0.010**
±	0.060	0.026	0.034	0.000	0.010
HRNet	0.072	0.028	**0.036**	**0.000**	**0.010**
±	0.100	0.080	0.029	0.000	0.010

(b) Segmentation images without cell debris

Model	JCD	RDD	RCD	ISF	ASF
U-Net	**0.028**	**0.008**	0.034	**0.000**	**0.000**
±	0.010	0.008	0.032	0.000	
HRNet	0.029	**0.008**	**0.033**	**0.000**	**0.000**
±	0.011	0.007	0.026	0.000	

(c) Optimized parameters

Parameter	U-Net	HRNet
Backbone	ResNet 34	W48
Transfer learning	Yes	Yes
Data augmentation	Yes	Yes
Resize factor	1/2	1/2
Loss function	Dice	Cross-Entropy
Optimizer	RAdam & Lookahead	Adam

U-Net vs HRNet

The automatic segmentations with the final optimized U-Net and HRNet model are applied to the test data set. While this test data set is not taken into account during training of the models, the segmentations display virtually the same high accuracy on these new images as for the validation data set, see see Tab. 1a and Fig. SM.15 cf. Fig. SM.13 and Fig. SM.14. Mostly, the standard deviations of the metrics are higher in the test data set, though the JCD remains even with this deviation well below 20%. As for the validation data set, the U-Net performs slightly better than the HRNet on the test data. The JCD and RDD for the HRNet are 0.01 higher, and their standard deviation is 0.04 higher. Two representative examples of the automatic segmentation by the U-Net are shown in Fig. 1: The overlap with the manual segmentation is excellent for standard size and larger spheroids obscured by cell debris, see Fig. 1 top row, and sufficient for small, heavily obscured spheroids, see bottom row. In the latter case, the model does not segment the spheroid boundary accurately but detects the spheroid at the correct position JCD = 23% with a comparable size RDD = 7.5% within the much larger and often darker cloud of surrounding cell debris. In such difficult cases, similar variations are observed upon manual segmentation by different biological experts.

While the models are mainly trained on images with treated spheroids which are obscured by cell debris, they also accurately segment untreated spheroids with clear boundary and clean background, see Tab. 1b: On a test set of 100 images of untreated spheroids, the accuracy of both models is nearly the same with a JCD below 3%. In comparison, a recent deep-learning approach for spheroids with clear boundary and clean background with focus on generalizability to different experimental conditions and microscopes, achieved a mean JCD of 8% with a standard deviation of 12% for bright-field microscopic images [31]. This suggests that the standard case of clean images without cell debris is included in our training of images with heavily obscured spheroids.

The final U-Net model has a size of 158 MB and the final HRNet model a size of 251 MB. For both models, it takes about 1.8 seconds to segment one image on the CPU (Intel(R) Core(TM) i7-4770). When the segmentation is performed serially on the GPU (NVIDIA GeForce RTX 3080) the U-Net needs only 0.03 seconds per image and the HRNet 0.08 seconds. As computation time for both models is comparable, the U-Net is chosen for the automatic segmentation due to its slightly higher accuracy in out setting. Note that in a recently published deep-learning approach, the comparison of HRNet and U-Net implied that HRNet achieved the highest accuracy [31].

Further independent validation

In addition to the standard check with the Hold-out test data set, we systematically validate the automatic segmentation with the optimized U-Net on larger, independent Hold-out test data sets of two cell types of head-and-neck cancer, see Fig. 4. This data set contains 6574 images of FaDu or SAS spheroids treated with different combinations and doses of X-ray irradiation and hyperthermia [16]. These images were manually segmented by another biological expert (human H2) independent from the original training/validation/test-data set used to develop the automatic segmentation (human H1). For the majority of images, we find excellent overlap between this manual and the automatic segmentation, quantified by a JCD around 0.1, see Fig. 4a. Larger deviations are mostly observed for images with smaller spheroids, i.e., below the size of standard spheroids for treatment (diameter 370 − 400 μm according to [15–17]). To make the evaluation intuitive for the biological experts, who are less familiar with the JCD defined in Eq. (1), we additionally introduce a measure of the average radial error Δr based on the automatically segmented (predicted) domain P and the manually segmented (target) domain T

$$\Delta r = \sqrt{\pi^{-1}\left(|P \cup T| - |P \cap T|\right) + \frac{d_T^2}{4}} - \frac{d_T}{2} \qquad (3)$$

(a)

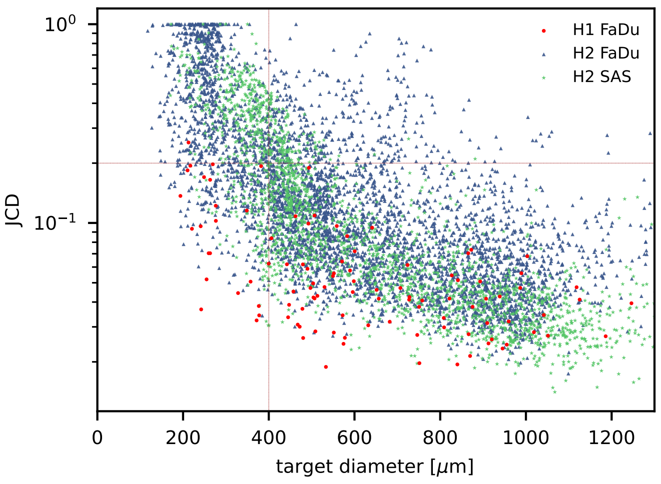

(b)

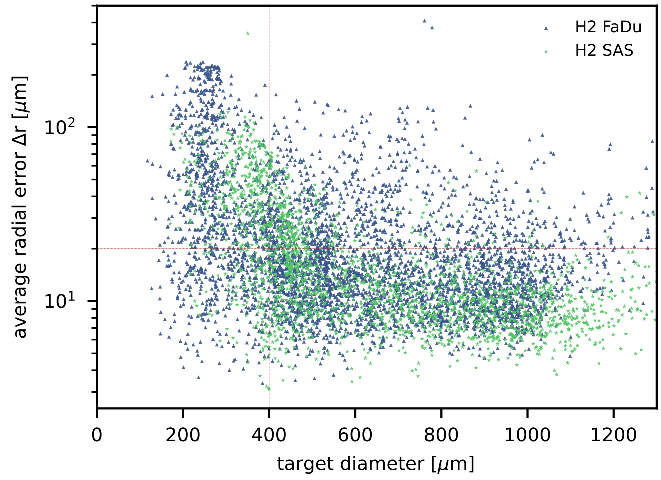

Figure 4. Validation of the automatic segmentation with the optimized U-Net on larger, independent data sets shows high accuracy for the majority of cases. (a) JCD and (b) average radial error Δr over diameter of the manually segmented (target) spheroid d_T for 6574 images of FaDu (blue triangles) and SAS (green stars) spheroids treated with different combinations and doses of X-ray irradiation and hyperthermia [16]. Manual segmentation is performed by a second biological expert (human H2, blue triangles and green stars) independently from the manual segmentation (human H1, red dots) of the training, validation and test data sets. (Results for 104 images of test data set is displayed as red dots for comparison.) Note that the segmentation is developed only based on images of FaDu spheroids. The majority of deviations are small (JCD < 0.2, $\Delta r < 20$ µm, red horizontal lines as guide to the eye), average (median) values are JCD $= 0.17(0.09) \pm 0.2$, $\Delta r = 25(15) \pm 32$ µm for the whole data set and JCD $= 0.1(0.07) \pm 0.09$, $\Delta r = 18(13) \pm 17$ µm for spheroids larger $d_T \geq 400$ µm (red vertical lines as guide to the eye) than the initial, standard size of spheroids. Larger imprecisions for smaller spheroids are due to biologically difficult, unclear, or ambiguous cases, see text.

with the average (target) diameter d_T of the manually segmented domain. The error Δr quantifies the thickness a circular layer with the size of the mismatched area $|P \cup T| - |P \cap T| = |P \setminus T| + |T \setminus P|$ around a circle with the target area $|T|$ would have, see Fig. SM.16 for an illustration. Since additional $P \setminus T$ and missing areas $T \setminus P$ do not compensate each other in Eq. (3), the error Δr is considerably larger than the radial error implied by the relative difference between target and predicted diameter RDD, see Fig. SM.17. The average radial error Δr reported in Fig. 4b assesses the segmentation analogous to the JCD with majority of errors $\Delta r < 20$ µm and larger deviations mostly for spheroids with diameter clearly smaller than before treatment ($d_T \leq 370 - 400$ µm). For reference, note that the resolution of the images is 2.04 µm/px, i.e., a deviation of 20 µm corresponds to 10px (< 1% deviation with respect to the original 1300×1030 image) or 5px in the half-resolution image for application of the U-Net.

Only a few images with larger spheroids exhibit JCD bigger than 0.2. Most of these are ambiguous cases, with two spheroids attached to each other, which are inconsistently recognized as either one or two spheroids even by the human, see examples with JCD = 0.28 – 0.34 and 0.42 in Fig. SM.4. While the training data set did not contain such double-spheroid cases, the optimized U-Net often segments them correctly, see examples with JCD = 0.08, 0.12 in Figs. SM.2 and SM.3, respectively. Apart from this particular case, most images with larger deviations between the segmentations refers to smaller spheroids with surrounding cell debris. Note that smaller spheroids without cell debris, i.e., untreated or shortly after treatment, do not exhibit such deviations but are accurately segmented. To thoroughly examine these larger deviations in more detail, three biological experts independently segmented 101 randomly selected images with spheroid diameter $d_T \leq 400$ µm. For these 101 images the JCDs between the humans and the U-Net and between different humans are compared in Fig. 5. We find that on average the discrepancies between humans and U-Net are comparable to the variations across segmentations from different humans. This implies that these images represent biologically difficult, unclear, or ambiguous cases. It suggests that the larger JCD observed for some smaller spheroids rather reflects this uncertainty and not a low performance of the U-Net. Accordingly, the evaluation in Fig. 4 refers to a worse-case scenario, as for each spheroid, the biological expert intentionally segmented all images over time, while in practice, segmentation is only required and performed for a fraction of these images, in particular for clearly distinguishable and larger spheroids.

Finally, we also test the automatic segmentation with the optimized U-Net on eight published test data sets from previous deep-learning approaches [22, 29, 31], see Tab. SM.2. Note that the total of 496 images originates from different conditions, including brightfield and fluorescence microscopy, RGB and 16-bit gray-scale, and different microscopes, magnifications, image resolutions, and cell models, but do not contain significant cell debris. While our automatic segmentation performs well on roughly half of the data sets, sometimes surpassing the original model corresponding to the data set, two types of images turn out problematic: (i) images with ambiguous ground truth and (ii) images on which the spheroids appears semi-transparent, with individual cells being visible throughout the spheroid. However, classical segmentation techniques work sufficiently well for both types of images (i) and (ii), making the use of deep-learning approaches in these cases unnecessary. In detail, images of type (i) contain spheroids with peculiar intensity profiles, i.e., with a compact sphere-like core surrounded by a flat patch. The corresponding published ground truth just defines the outer boundary of these patches as the boundary of the spheroid, while our automatic segmentation restricts the spheroid to the compact core. Either choice may be unreasonable depending on the goal of the analysis. The outer boundary of the patches allows computation of the projected area of the spheroid while ignoring the considerable variations in thickness encoded in the intensity across the spheroid. Accordingly, it is not suited to estimate the spheroid volume necessary to evaluate volume growth. Presumably, the compact core contains most of the three-dimensional organized cells and cellular volume. It is the main component exhibiting metabolic gradients affecting therapy response [4–8], but certainly, it underestimates the total cell volume. Segmentation of such incompletely formed spheroids is debatable, even biologically, as the formation of proliferative and metabolic gradients remains ambiguous and may depend on the question pursued with the experiment. For images of type (ii), the apparent visibility of individual cells throughout the spheroid may be due to its small size or the chosen microscopy

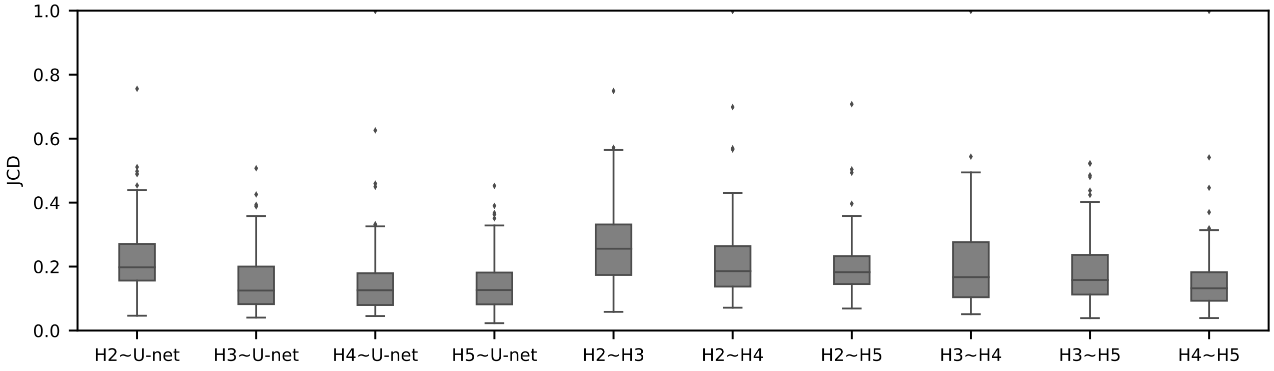

Figure 5. For treated spheroids smaller than the initial, standard size before treatment ($d \leq 400$ µm) deviations from the manual segmentation are not higher than variations across segmentations by different humans, suggesting that the segmentation of images with small spheroids surrounded by heavy debris is often difficult or ambiguous: Compared are segmentations from the optimized U-Net and 4 independent human experts (H2-H5) for the same 101 images, which are randomly selected from the pool of small spheroids of the extended Hold-out test data set, see Fig. 4. The Friedman test score of all JCDs 217.2 ($p \ll 0.001$) indicates significant differences among the pairwise segmentation deviations. The order according to the average JCD is (from low to high values) H5~U-Net, H3~U-Net, H3~H5, H2~H5 < H4~H5, H4~U-Net, H2~U-Net, H3~H4, H2~H4, H2~H3, where the < indicates a significant ($p < 0.005$) difference between the sets of JCDs according to a Dunn-Bonferroni pairwise post-hoc test.

method.

Discussion

We develop an automatic segmentation for images of 3D tumor spheroids both with and without (radio)therapy. We systematically validate the automatic segmentation on larger, independent Hold-out test data sets of two cell types of head-and-neck cancer spheroid types, including combinations with hyperthermia treatment. For most images, we find excellent overlap between manual and automatic segmentation. These include clearly discernable spheroids and the previously neglected cases of spheroids critically obscured by cell debris. For images showing poor overlap of the segmentations, we demonstrate that this error is comparable to the variations between segmentations from different biological experts (inter-observer variability). This suggests that considerable deviations between automatic and manual segmentations do not necessarily reflect a low performance of the former but rather a general uncertainty or ambiguity in spheroid identification.

While the accuracy of (spheroid) segmentations is usually only quantified in terms of the JCD or Jaccard index (IoU), we choose to additionally report the corresponding implications on the spheroid diameter derived from the segmentation by Δr and RDD. This makes the evaluation more intuitive for biological experts, as the spheroid diameter is the central metric for analysis. For instance, the spheroid volume required for growth curve documentation and growth delay is estimated from this diameter, which is connected to the projected area of the spheroid. We do not explicitly translate the evaluation to the corresponding spheroid area and volume. However, this may be estimated from basic scaling arguments, i.e., area and volume scale square and cubic with the diameter. The circularity of the spheroid, as quality control for its desired spherical shape, is taken into account by the RCD. All of these metrics will converge to their correct value, when the JCD goes to 0 (or Jaccard index to 1).

In fact, it should be pointed out that the imprecisions of the segmentation have a smaller effect on the quantification of therapy response than suggested by the reported JCD: As most informative parameters in long-term spheroid-based assays are the average diameters, volumes, and circularities of the spheroids. Thus, the relevant metric to estimate the error of the automatic analysis is not the JCD but the RDD and RCD, which are considerably smaller as even an imprecise segmentation can result in the correct size or shape of the spheroid. Furthermore, higher deviations are primarily observed for for treated 3D cultures with diameters below the initial diameter of standard spheroids $d_T = 370 - 400$ µm before treatment. These are usually images of spheroids in the final steps of detachment or before cell re-aggregation, growth recovery, and spheroid relapse. In experimental practice, such cases are typically not segmented as they are less relevant for the analytical endpoints. For instance, computation of growth delay requires accuracy of the spheroid segmentation immediately before treatment (for images without obscuring cell debris) and at large spheroid sizes $d_T > 600$ µm, while the images in between have no impact on the growth delay. Moreover, the validation in Fig. 4 is based on images from the time series of relapsed and controlled spheroids. However, in practical routine, segmentations of complete time series are only performed for growth curves and growth delay assessment in spheroid populations at 100 % growth recovery. The average JCD is substantially smaller if the validation is restricted to these cases.

It is important to note that the initial automatic segmentation is driven by images of only one HNSCC spheroid model (FaDu) with and without treatment. However, we show that it works equally well for spheroids from another cell type (SAS), although these SAS spheroids display a different peripheral shape during regrowth after treatment. Beyond the systematic validation highlighted herein, the automatic segmentation has been continuously tested and applied by several biological/biomedical experts and researchers for over a year during their ongoing experiments and to retrospectively reanalyze MCTS from earlier studies. So far, it has been reported that resegmentation is only necessary in a small fraction of cases, mainly due to optical artifacts, like out-of-focus images. Overall, the segmentation has in the meantime been successfully applied to numerous untreated multicellular spheroid types of different tumor entities and cell lines, respectively (FaDu, SAS - head and neck; Panc-02(mouse), Panc-1, PaTu 8902 - pancreas; HCT-116, HT29 - colon; A549, NCI-H23, NCI-H460 - lung; BT474 - breast; LNCap, DU145 - prostate; U87-MG, U138-MG, U251-MG - brain/GBM; Hek293 - kidney). These spheroids ranged between $200 - 1000$ µm in diameter and images were taken at different magnifications and resolutions (e.g., 1300×1030, 1388×1040 1920×1440, 1920×1216 and $1.6 - 2.6$ µm/pixel) as single or Z-stack images at diverse microscopic devices (Axiovert 200M, AxioObserver Z1 - both from Zeiss; BioTek Cytation 5 Cell Imaging Multimode Reader - Agilent). Many more spheroid types are in the pipeline for implementation. The developed automatic segmentation has also already been applied to contour the images of selected spheroid types, e.g., FaDu, SAS, Panc-02 or DU145, after various treatments such as radiotherapy (X-ray, proton), hyperthermia, chemotherapy and combinatorial treatment.

We also test the automatic segmentation on eight published test

data sets from previous deep-learning approaches [22, 29, 31]. We observe a high accuracy, except for special cases, which, however, turn out to be well segmentable by classical techniques. While the automatic segmentation has been continuously tested for over a year on different cell lines, microscopes, and treatments, it does not necessarily generalize to arbitrary experimental conditions and imaging, which will be the focus of future improvements. However, this problem of domain shift, which can always arise when a model is applied to data sets with a different data distribution than the training data, is also an ongoing challenge for deep-learning models focused on images with clear spheroids and clean background [49]. In contrast, the focus of this study is the inclusion of the case of treated spheroids surrounded by severe cell debris, which is frequent after treatment but often neglected in automatic segmentation.

Note that the automatic segmentation's accuracy and reliability may not be exclusive to the finally chosen network architecture and hyperparameters. Indeed, we find that two very different network architectures, U-Net and HRNet, achieve very similar performance, which is consistent with previously reported optimizations of deep-learning models [31, Tab. 2] [29, Tab. 3]. The segmentation's accuracy also seems relatively insensitive to the choice of several hyperparameters, e.g., loss function, optimizer function, and, to some extent, the resize factor. Instead, it is plausible, that applying challenging training data, i.e., images with extensive, severe debris, is crucial for the performance of the resulting automatic segmentation. The network trained primarily on such images also works on images with clearly visible spheroids. Hence, the data sets compiled and annotated for this work are also publicly provided to support future improvements of spheroid segmentations. This data set can facilitate the development of more individual, more customized models, e.g., using the recently introduced nnU-Net tool [62].

The automatic segmentation can be incorporated into existing or future tools for spheroid analysis or medical image analysis as basis for further feature extraction including perimeter, complexity, and multiparametric analysis [47]. The segmentation provides a basis for the development of an automatic classification of spheroid image time series into control and relapse and could support machine learning methods to forecast tumor spheroid fate early. To make the deep-learning model available, we provide a minimal tool with graphical user interface. The ONNX Runtime [63] is used to compile the model and to take it into production.

Availability of source code and requirements (optional, if code is present)

- Project name: Spheroidsegdedeb (Spheroid segmentation despite debris)
- Project home page: `https://igit.informatik.htw-dresden.de/aagef650/spheroidsegdedeb`
- Operating system(s): Platform independent
- Programming language: Python >3.8.10
- Other requirements: Additionally download the model from `https://wwwpub.zih.tu-dresden.de/~s6079592/SEGMODEL.onnx`. Requirements and usage are documented in the provided README and some example images for testing are included in the corresponding folder.
- License: GNU GPL

Data availability

Training, validation, and test data used for this work are available at `https://wwwpub.zih.tu-dresden.de/~s6079592/data.zip` and `https://wwwpub.zih.tu-dresden.de/~s6079592/extended_data.zip` as well as the code to train and evaluate the model (see above repo training_scripts/). All networks optimized for different hyperparameters are available in the .pth format at `https://wwwpub.zih.tu-dresden.de/~s6079592/models/`.

Declarations

List of abbreviations

Ambiguous Spheroid Fraction (ASF), fully convolutional network (FCN), Invalid Spheroid Fraction (ISF), Jaccard Distance (JCD), Multicellular tumor spheroids (MCTS), Relative Circularity Deviation (RCD), Relative Diameter Deviation (RDD), Supplemental material (SM)

Competing Interests

The authors declare that they have no competing interests.

Funding

The authors acknowledge that this research has been co-financed by the EU, the European Social Fund (ESF), and by tax funds on the basis of the budget passed by the Saxon state parliament (project SAB-Nr. 100382145) and the Bundesministerium für Bildung und Forschung (BMBF 16dkwn001a/b). The funders had no role in study design, data collection and analysis, decision to publish, or preparation of the manuscript.

Author's Contributions

Conceptualization, A.V.-B., L.A.K.-S., and S.L.; methodology, A.V.-B., S.L., S.M., L.A.K.-S., and M.S..; software, M.S., K.L., and S.L.; validation, W.C., K.L., S.M., and S.L.; formal analysis, M.S., A.V.-B., and S.L.; investigation, M.S., K.L., and S.L.; resources, L.A.K.-S., and A.V.-B.; data curation, M.S., W.C., K.L., and S.L.; writing – original draft preparation, M.S., A.V.-B. and S.L.; writing – review and editing, A.V.-B., W.C., S.L., S.M., and L.A.K.-S.; visualization, M.S., W.C., and S.L.; supervision, L.A.K.-S., A.V.-B. and S.L.; project administration, L.A.K.-S. and A.V.-B.; funding acquisition, L.A.K.-S. and A.V.-B. All authors have read and agreed to the published version of the manuscript.

Acknowledgements

We are grateful to M. Wondrak, S. Al-Jamei, N. El-Refai, R. Joseph, and S. L. Prieto for sharing their expertise on tumor spheroid contouring.

References

1. Franke F, Michlíková S, Aland S, Kunz-Schughart LA, Voss-Böhme A, Lange S. Efficient Radial-Shell Model for 3D Tumor Spheroid Dynamics with Radiotherapy. Cancers 2023;15(23):5645. `https://www.mdpi.com/2072-6694/15/23/5645`.
2. Brüningk SC, Ziegenhein P, Rivens I, Oelfke U, ter Haar G. A cellular automaton model for spheroid response to radiation and hyperthermia treatments. Sci Rep 2019;9(1):17674. `https://www.nature.com/articles/s41598-019-54117-x`.
3. Brüningk SC, Rivens I, Box C, Oelfke U, ter Haar G. 3D tumour spheroids for the prediction of the effects of radiation and hyperthermia treatments. Sci Rep 2020;10(1):1653. `https://www.nature.com/articles/s41598-020-58569-4`.

4. Riffle S, Hegde RS. Modeling tumor cell adaptations to hypoxia in multicellular tumor spheroids. Journal of Experimental & Clinical Cancer Research 2017;36(1):102. https://doi.org/10.1186/s13046-017-0570-9.
5. Leek R, Grimes DR, Harris AL, McIntyre A. Methods: Using Three-Dimensional Culture (Spheroids) as an In Vitro Model of Tumour Hypoxia. In: Koumenis C, Coussens LM, Giaccia A, Hammond E, editors. Tumor Microenvironment Advances in Experimental Medicine and Biology, Springer International Publishing; 2016. p. 167–196.
6. Hirschhaeuser F, Menne H, Dittfeld C, West J, Mueller-Klieser W, Kunz-Schughart LA. Multicellular tumor spheroids: An underestimated tool is catching up again. Journal of Biotechnology 2010;148(1):3–15. https://www.sciencedirect.com/science/article/pii/S0168165610000398.
7. Friedrich J, Ebner R, Kunz-Schughart LA. Experimental antitumor therapy in 3-D: Spheroids - old hat or new challenge? International Journal of Radiation Biology 2007;83(11):849–871. https://doi.org/10.1080/09553000701727531.
8. Kunz-Schughart LA, Freyer JP, Hofstaedter F, Ebner R. The Use of 3-D Cultures for High-Throughput Screening: The Multicellular Spheroid Model. J Biomol Screen 2004;9(4):273–285. https://doi.org/10.1177/1087057104265040.
9. Pérez-García VM, Calvo GF, Bosque JJ, León-Triana O, Jiménez J, Pérez-Beteta J, et al. Universal scaling laws rule explosive growth in human cancers. Nature Physics 2020;p. 1–6. https://www.nature.com/articles/s41567-020-0978-6.
10. Howard-Flanders P, Alper T. The Sensitivity of Microorganisms to Irradiation under Controlled Gas Conditions. Radiat Res 1957;7(5):518–540. https://www.jstor.org/stable/3570400.
11. Muz B, Puente Pdl, Azab F, Azab AK. The role of hypoxia in cancer progression, angiogenesis, metastasis, and resistance to therapy. Hypoxia med j 2015 Dec;3:83–92.
12. Rakotomalala A, Escande A, Furlan A, Meignan S, Lartigau E. Hypoxia in Solid Tumors: How Low Oxygenation Impacts the "Six Rs" of Radiotherapy. Front Endocrinol 2021;12. https://www.frontiersin.org/articles/10.3389/fendo.2021.742215.
13. Li C, Wiseman L, Okoh E, Lind M, Roy R, Beavis AW, et al. Exploring hypoxic biology to improve radiotherapy outcomes. Expert Rev Mol Med 2022 Jan;24:e21. https://www.cambridge.org/core/journals/expert-reviews-in-molecular-medicine/article/abs/exploring-hypoxic-biology-to-improve-radiotherapy-outcomes/A367ECCBE52B8E6D5D5B81FAC7C92B30.
14. Peirsman A, Blondeel E, Ahmed T, Anckaert J, Audenaert D, Boterberg T, et al. MISpheroID: a knowledgebase and transparency tool for minimum information in spheroid identity. Nat Methods 2021;18(11):1294–1303. https://www.nature.com/articles/s41592-021-01291-4.
15. Chen O, Manig F, Lehmann L, Sorour N, Löck S, Yu Z, et al. Dual role of ER stress in response to metabolic co-targeting and radiosensitivity in head and neck cancer cells. Cell Mol Life Sci 2021;78(6):3021–3044. https://doi.org/10.1007/s00018-020-03704-7.
16. Chen O, Michlíková S, Eckhardt L, Wondrak M, De Mendoza AM, Krause M, et al. Efficient Heat Shock Response Affects Hyperthermia-Induced Radiosensitization in a Tumor Spheroid Control Probability Assay. Cancers 2021;13(13):3168. https://www.mdpi.com/2072-6694/13/13/3168.
17. Hinrichs CN, Ingargiola M, Käubler T, Löck S, Temme A, Köhn-Luque A, et al. Arginine Deprivation Therapy: Putative Strategy to Eradicate Glioblastoma Cells by Radiosensitization. Mol Cancer Ther 2018;17(2):393–406. https://mct.aacrjournals.org/content/17/2/393.
18. Vynnytska-Myronovska B, Bobak Y, Garbe Y, Dittfeld C, Stasyk O, Kunz-Schughart LA. Single amino acid arginine starvation efficiently sensitizes cancer cells to canavanine treatment and irradiation. International Journal of Cancer 2012;130(9):2164–2175. https://onlinelibrary.wiley.com/doi/abs/10.1002/ijc.26221.
19. Kummer B, Löck S, Gurtner K, Hermann N, Yaromina A, Eicheler W, et al. Value of functional in-vivo endpoints in preclinical radiation research. Radiotherapy and Oncology 2021;158:155–161. https://www.sciencedirect.com/science/article/pii/S0167814021060874.
20. Baumann M, Petersen C, Krause M. TCP and NTCP in preclinical and clinical research in Europe. Rays 2005;30(2):121–126.
21. Hoque MT, Windus LCE, Lovitt CJ, Avery VM. PCaAnalyser: A 2D-Image Analysis Based Module for Effective Determination of Prostate Cancer Progression in 3D Culture. PLoS ONE 2013;8(11):e79865. https://journals.plos.org/plosone/article?id=10.1371/journal.pone.0079865.
22. Ivanov DP, Parker TL, Walker DA, Alexander C, Ashford MB, Gellert PR, et al. Multiplexing Spheroid Volume, Resazurin and Acid Phosphatase Viability Assays for High-Throughput Screening of Tumour Spheroids and Stem Cell Neurospheres. PLoS ONE 2014;9(8):e103817. https://journals.plos.org/plosone/article?id=10.1371/journal.pone.0103817.
23. Monjaret F, Fernandes M, Duchemin-Pelletier E, Argento A, Degot S, Young J. Fully Automated One-Step Production of Functional 3D Tumor Spheroids for High-Content Screening. SLAS Technology 2016;21(2):268–280. https://www.sciencedirect.com/science/article/pii/S247263032201384X.
24. Rueden CT, Schindelin J, Hiner MC, DeZonia BE, Walter AE, Arena ET, et al. ImageJ2: ImageJ for the next generation of scientific image data. BMC Bioinformatics 2017;18(1):529. https://doi.org/10.1186/s12859-017-1934-z.
25. Moriconi C, Palmieri V, Di Santo R, Tornillo G, Papi M, Pilkington G, et al. INSIDIA: A FIJI Macro Delivering High-Throughput and High-Content Spheroid Invasion Analysis. Biotechnology Journal 2017;12(10):1700140. https://onlinelibrary.wiley.com/doi/abs/10.1002/biot.201700140.
26. Boutin ME, Voss TC, Titus SA, Cruz-Gutierrez K, Michael S, Ferrer M. A high-throughput imaging and nuclear segmentation analysis protocol for cleared 3D culture models. Sci Rep 2018;8(1):11135. https://www.nature.com/articles/s41598-018-29169-0.
27. Chen W, Wong C, Vosburgh E, Levine AJ, Foran DJ, Xu EY. High-throughput Image Analysis of Tumor Spheroids: A User-friendly Software Application to Measure the Size of Spheroids Automatically and Accurately. J Vis Exp 2014;(89):51639. https://www.ncbi.nlm.nih.gov/pmc/articles/PMC4212916/.
28. Piccinini F. AnaSP: A software suite for automatic image analysis of multicellular spheroids. Computer Methods and Programs in Biomedicine 2015;119(1):43–52. https://www.sciencedirect.com/science/article/pii/S0169260715000309.
29. Piccinini F, Peirsman A, Stellato M, Pyun JC, Tumedei MM, Tazzari M, et al. Deep learning-based tool for morphotypic analysis of 3d multicellular spheroids. J Mech Med Biol 2023;23(6):2340034. https://www.worldscientific.com/doi/abs/10.1142/S0219519423400341.
30. Hou Y, Konen J, Brat DJ, Marcus AI, Cooper LAD. TASI: A software tool for spatial-temporal quantification of tumor spheroid dynamics. Sci Rep 2018;8(1):7248. https://www.nature.com/articles/s41598-018-25337-4.
31. Lacalle D, Castro-Abril HA, Randelovic T, Domínguez C, Heras J, Mata E, et al. SpheroidJ: An Open-Source Set of Tools for Spheroid Segmentation. Comput Methods Programs Biomed 2021;200:105837. https://www.sciencedirect.com/science/article/pii/S0169260720316709.
32. Perini G, Rosa E, Friggeri G, Di Pietro L, Barba M, Parolini O, et al. INSIDIA 2.0 High-Throughput Analysis of 3D Cancer Models: Multiparametric Quantification of Graphene Quantum Dots Photothermal Therapy for Glioblastoma and Pancreatic Cancer. International Journal of Molecular Sciences 2022;23(6):3217.

https://www.mdpi.com/1422-0067/23/6/3217.
33. Jadon S. A survey of loss functions for semantic segmentation. In: 2020 IEEE Conference on Computational Intelligence in Bioinformatics and Computational Biology (CIBCB); 2020. p. 1–7.
34. Yen JC, Chang FJ, Chang S. A new criterion for automatic multilevel thresholding. IEEE Transactions on Image Processing 1995;4(3):370–378. https://ieeexplore.ieee.org/document/366472.
35. Otsu N. A Threshold Selection Method from Gray-Level Histograms. IEEE Transactions on Systems Man and Cybernetics 1979;9(1):62–66. https://ieeexplore.ieee.org/document/4310076.
36. Roerdink JBTM, Meijster A. The Watershed Transform: Definitions, Algorithms and Parallelization Strategies. Fundamenta Informaticae 2000;41(1):187–228. https://content.iospress.com/articles/fundamenta-informaticae/fi41-1-2-07.
37. Duda RO, Hart PE. Use of the Hough transformation to detect lines and curves in pictures. Commun ACM 1972;15(1):11–15. https://dl.acm.org/doi/10.1145/361237.361242.
38. Caselles V, Kimmel R, Sapiro G. Geodesic Active Contours. International Journal of Computer Vision 1997;22(1):61–79. https://doi.org/10.1023/A:1007979827043.
39. Cisneros Castillo LR, Oancea AD, Stüllein C, Régnier-Vigouroux A. A Novel Computer-Assisted Approach to evaluate Multicellular Tumor Spheroid Invasion Assay. Sci Rep 2016;6(1):35099. https://www.nature.com/articles/srep35099.
40. Vinci M, Gowan S, Boxall F, Patterson L, Zimmermann M, Court W, et al. Advances in establishment and analysis of three-dimensional tumor spheroid-based functional assays for target validation and drug evaluation. BMC Biology 2012;10(1):29. https://doi.org/10.1186/1741-7007-10-29.
41. Cornelissen F, Cik M, Gustin E. Phaedra, a Protocol-Driven System for Analysis and Validation of High-Content Imaging and Flow Cytometry. SLAS Discovery 2012;17(4):496–508. https://www.sciencedirect.com/science/article/pii/S2472555222076341.
42. Sadanandan SK, Karlsson J, Wählby C. Spheroid Segmentation Using Multiscale Deep Adversarial Networks. In: 2017 IEEE International Conference on Computer Vision Workshops (ICCVW); 2017. p. 36–41. https://ieeexplore.ieee.org/document/8265222.
43. Celli JP, Rizvi I, Blanden AR, Massodi I, Glidden MD, Pogue BW, et al. An imaging-based platform for high-content, quantitative evaluation of therapeutic response in 3D tumour models. Sci Rep 2014;4(1):3751. https://www.nature.com/articles/srep03751.
44. Piccinini F, Tesei A, Zanoni M, Bevilacqua A. ReViMS: Software tool for estimating the volumes of 3-D multicellular spheroids imaged using a light sheet fluorescence microscope. BioTechniques 2017;63(5):227–229. https://www.future-science.com/doi/10.2144/000114609.
45. Ngo TKN, Yang SJ, Mao BH, Nguyen TKM, Ng QD, Kuo YL, et al. A deep learning-based pipeline for analyzing the influences of interfacial mechanochemical microenvironments on spheroid invasion using differential interference contrast microscopic images. Materials Today Bio 2023;23:100820. https://www.sciencedirect.com/science/article/pii/S2590006423002806.
46. Chen Z, Ma N, Sun X, Li Q, Zeng Y, Chen F, et al. Automated evaluation of tumor spheroid behavior in 3D culture using deep learning-based recognition. Biomaterials 2021;272:120770. https://www.sciencedirect.com/science/article/pii/S0142961221001265.
47. Zhuang M, Chen Z, Wang H, Tang H, He J, Qin B, et al. AnatomySketch: An Extensible Open-Source Software Platform for Medical Image Analysis Algorithm Development. J Digit Imaging 2022;35(6):1623–1633. https://doi.org/10.1007/s10278-022-00660-5.
48. Akshay A, Katoch M, Abedi M, Shekarchizadeh N, Besic M, Burkhard FC, et al. SpheroScan: a user-friendly deep learning tool for spheroid image analysis. GigaScience 2023;12:giad082. https://doi.org/10.1093/gigascience/giad082.
49. García-Domínguez M, Domínguez C, Heras J, Mata E, Pascual V. Deep style transfer to deal with the domain shift problem on spheroid segmentation. Neurocomputing 2024;569:127105. https://www.sciencedirect.com/science/article/pii/S0925231223012286.
50. Howard J, Gugger S. Fastai: A Layered API for Deep Learning. Information 2020 Feb;11(2):108. https://www.mdpi.com/2078-2489/11/2/108.
51. Castillo DL, SemTorch; 2020. https://pypi.org/project/SemTorch/#description.
52. Long J, Shelhamer E, Darrell T. Fully convolutional networks for semantic segmentation. IEEE Computer Society; 2015. p. 3431–3440. https://www.computer.org/csdl/proceedings-article/cvpr/2015/07298965/12OmNy49sME.
53. Ronneberger O, Fischer P, Brox T. U-Net: Convolutional Networks for Biomedical Image Segmentation. In: Navab N, Hornegger J, Wells WM, Frangi AF, editors. Medical Image Computing and Computer-Assisted Intervention - MICCAI 2015 Lecture Notes in Computer Science, Springer International Publishing; 2015. p. 234–241.
54. Wang J, Sun K, Cheng T, Jiang B, Deng C, Zhao Y, et al. Deep High-Resolution Representation Learning for Visual Recognition. IEEE Trans Pattern Anal Mach Intell 2021;43(10):3349–3364.
55. Smith LN, A disciplined approach to neural network hyper-parameters: Part 1 – learning rate, batch size, momentum, and weight decay. arXiv; 2018. http://arxiv.org/abs/1803.09820.
56. Suzuki S, be K. Topological structural analysis of digitized binary images by border following. Computer Vision, Graphics, and Image Processing 1985 Apr;30(1):32–46. https://www.sciencedirect.com/science/article/pii/0734189X85900167.
57. Deng J, Dong W, Socher R, Li LJ, Li K, Fei-Fei L. Imagenet: A large-scale hierarchical image database. In: 2009 IEEE conference on computer vision and pattern recognition Ieee; 2009. p. 248–255.
58. Kingma DP, Ba J, Adam: A Method for Stochastic Optimization. arXiv; 2017. http://arxiv.org/abs/1412.6980, arXiv:1412.6980 [cs].
59. Liu L, Jiang H, He P, Chen W, Liu X, Gao J, et al., On the Variance of the Adaptive Learning Rate and Beyond. arXiv; 2021. http://arxiv.org/abs/1908.03265, arXiv:1908.03265 [cs, stat].
60. Zhang M, Lucas J, Ba J, Hinton GE. Lookahead Optimizer: k steps forward, 1 step back. In: Advances in Neural Information Processing Systems, vol. 32 Curran Associates, Inc.; 2019. https://proceedings.neurips.cc/paper/2019/hash/90fd4f88f588ae64038134f1eeaa023f-Abstract.html.
61. Buslaev A, Iglovikov VI, Khvedchenya E, Parinov A, Druzhinin M, Kalinin AA. Albumentations: Fast and Flexible Image Augmentations. Information 2020;11(2):125. https://www.mdpi.com/2078-2489/11/2/125.
62. Isensee F, Jaeger PF, Kohl SAA, Petersen J, Maier-Hein KH. nnU-Net: a self-configuring method for deep learning-based biomedical image segmentation. Nature Methods 2021;18(2):203–211. https://www.nature.com/articles/s41592-020-01008-z.
63. developers OR, ONNX Runtime; 2021. Version: 1.11.1. https://onnxruntime.ai/.

A Supplemental figures and tables (SM)

(a) Untreated spheroid w/o cell debris **(b)** Treated spheroid with cell debris

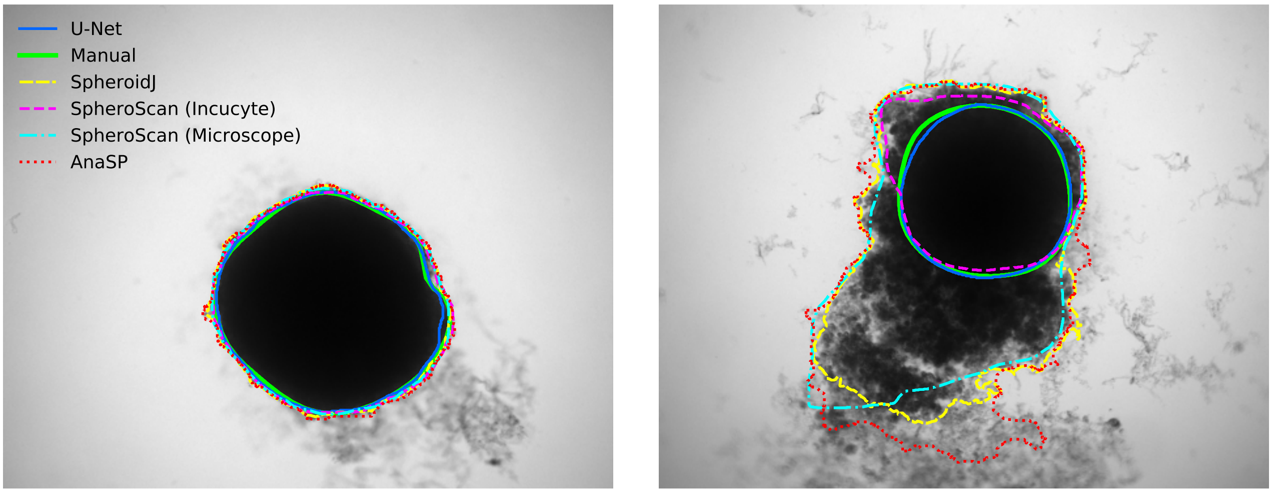

Figure SM.1. Example images illustrating the current challenge of segmenting tumor spheroids: (a) While previously developed deep-learning models generalize excellently to new image data with well-distinguished, unobscured spheroids, typical for untreated cultures, (b) these models fail for cases of detached/relapsing spheroids after radiotherapy (one of the most common cancer treatments) due to debris and dead cells obscuring the spheroid. Shown are representative images of FaDu spheroids (a) without treatment and (b) several days after radiotherapy. Different segmentations are indicated by their outer contours. In particular, the manually set ground truth (green solid line) is compared to the four most recent deep-learning models provided by SpheroidJ [31] (HRNet-Seg with HRNet W30 backbone, yellow close-dashed line), SpheroScan [48] (Region-based Convolutional Neural Network trained on images from IncuCyte Live-Cell Analysis System (purple dashed line) and ordinary microscope (torquoise dashed line) with recommended threshold 0.8), and AnaSP [29] (ResNet18, red dotted line). Only the U-Net presented in this manuscript (blue solid line) performs sufficiently in both scenarios. Size of each image corresponds to 2650 μm $\times$ 2100 μm with a resolution of 1300 $\times$ 1030 pixel.

Table SM.1. Statistical results reflecting the current challenge of segmenting tumor spheroids, corroborating Fig. SM.1: While previously developed deep-learning models generalize excellently to new image data with well-visible, unobscured spheroids (top line), these models fail for typical cases of detached/relapsing spheroids with debris of dead cells (bottom lines). Intersection over Union (IoU or Jaccard index) is reported (average (median) $\pm$ standard deviation) for each of the four most recent deep-learning models (provided by SpheroidJ [31], SpheroScan [48] (with recommended threshold 0.8), and AnaSP [29]) and each of our data sets. Only the U-Net presented in this manuscript performs sufficiently in both scenarios. Bold values highlight the optimum for each dataset.

Data set	#Images	Intersection over Union (IoU = 1-JCD)				
		U-Net	SpheroidJ [31]	SpheroScan (Incu) [48]	SpheroScan (Micro) [48]	AnaSP [29]
Spheroids without cell debris	200	**0.97(0.97)** ± **0.01**	0.89(0.90) ± 0.05	0.90(0.94) ± 0.16	0.72(0.92) ± 0.38	0.81(0.87) ± 0.22
Training	883	**0.94(0.95)** ± **0.05**	0.51(0.53) ± 0.27	0.68(0.86) ± 0.34	0.48(0.59) ± 0.37	0.46(0.44) ± 0.28
Validation	108	**0.95(0.96)** ± **0.04**	0.31(0.29) ± 0.19	0.59(0.80) ± 0.39	0.36(0.26) ± 0.32	0.28(0.25) ± 0.19
Test	104	**0.94(0.96)** ± **0.06**	0.41(0.38) ± 0.27	0.66(0.88) ± 0.36	0.48(0.50) ± 0.38	0.35(0.33) ± 0.24

Table SM.2. Further validation of the trained U-Net on wide variety of published test data sets from previous deep-learning models. While the trained U-Net performs well on on roughly half of the data sets or 38% of the images (with average IoU above 0.8), sometimes surpassing the original model corresponding to the data set, two types of images turn out problematic: (i) images with ambiguous ground truth (20% of images) for which the U-Net may actually segment reasonably, see main text for detailed discussion, and (ii) images on which the spheroids appears semi-transparent (42% of images), with individual cells being visible throughout the spheroid, potentially due to its small size or the chosen microscopy method. However, classical segmentation techniques work sufficiently well for both types of images (i) and (ii), making the use of deep-learning approaches in these cases unnecessary. For demonstration, the last two columns report the performance of the classical approach from the original publication and simple Otsu thresholding (sometimes after some image erosion for (ii)) performed by us for images of types (i) and (ii). Note that the notation for data sets from Refs. [22, 31] stands for brightfield/fluorescence microscopy (B/F), Nikon Eclipse/Leica DMi8/Olympus microscope (N,L,O), 2x/5x/10x magnification (2/5/10), and suspension/collagen culture (S/C). Bold values highlight the optimum for each dataset.

Data set	#Images	Comments	Intersection over Union (IoU = 1-JCD)			
			deep-learning model		classical segmentation	
			Original	U-Net	Original	Otsu thresholding
BO10S [22]	66		0.92 ± 0.03 [31]	**0.97(0.98)** ± **0.05**	0.94 ± 0.03 [31]	-
BN10S [31]	21		**0.97** ± **0.01**	0.89(0.89) ± 0.01	0.95 ± 0.01	0.84(0.85) ± 0.13
	84	ambiguous ground truth		0.61(0.62) ± 0.15		
BN2S [31]	154	semi-transparent	**0.96** ± **0.01**	0.30(0.27) ± 0.22	0.94 ± 0.02	0.87(0.95) ± 0.27
BL5S [31]	50	semi-transparent	0.75 ± 0.25	0.37(0.13) ± 0.39	0.64 ± 0.30	**0.85(0.93)** ± **0.20**
FN2S [31]	30		0.78 ± 0.20	0.91(0.94) ± 0.08	**0.82** ± **0.17**	-
	4	semi-transparent		0		0.83(0.83) ± 0.02
FL5C[31]	4		0.71 ± 0.30	0.92(0.91) ± 0.01	0.67 ± 0.17	-
	15	ambiguous ground truth		0.45(0.45) ± 0.16		**0.80(0.85)** ± **0.11**
FL5S [31]	50		0.70 ± 0.26	**0.91(0.91)** ± **0.05**	0.89 ± 0.07	-
AnaSP [29]	16		~ 0.92	0.97(0.98) ± 0.02		-
	2	semi-transparent		0.01(0.01) ± 0.01		0.97(0.97) ± 0.001

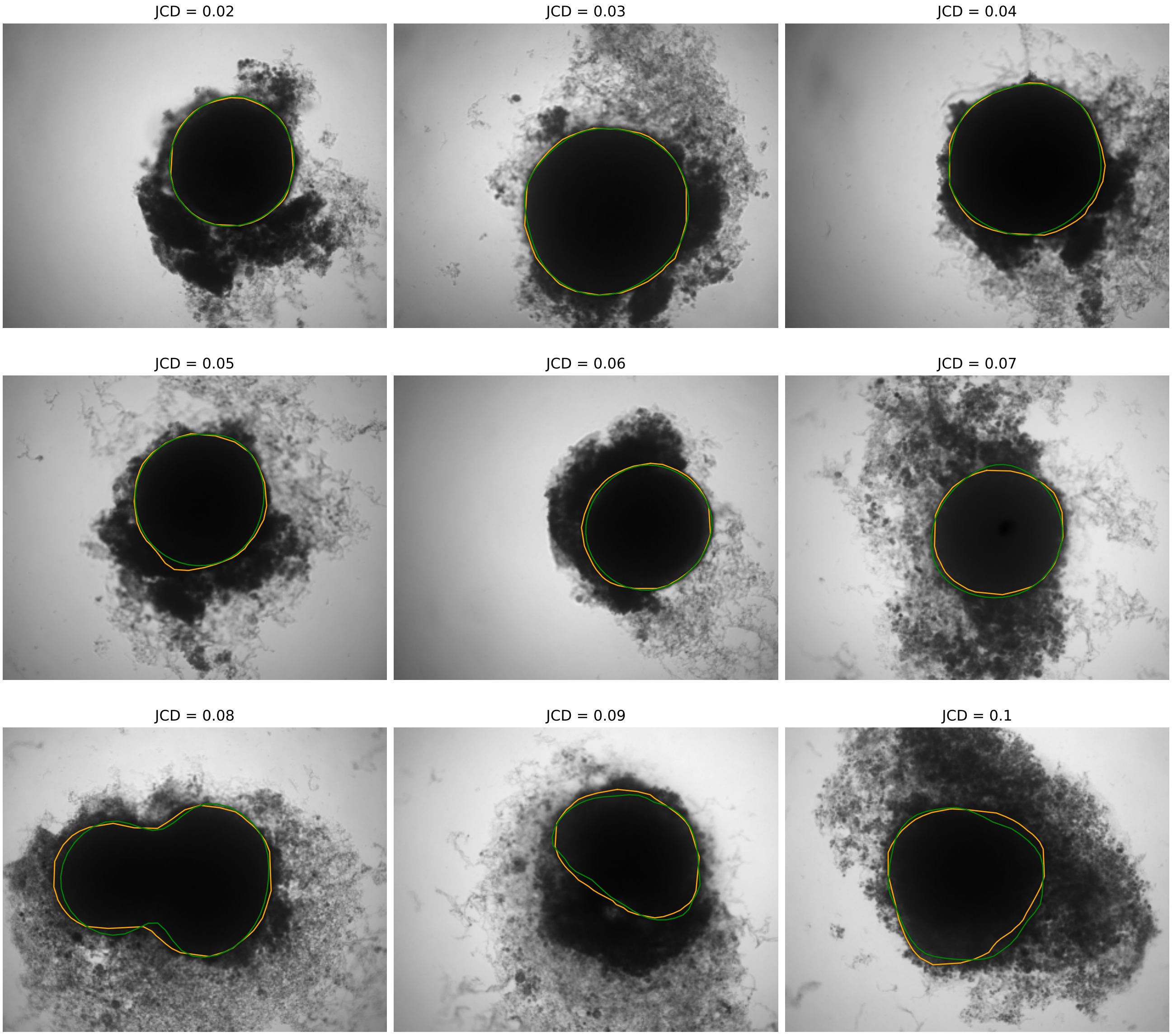

Figure SM.2. Selection of representative images as optical reference for JCD$\leq$ 0.1 with automatic segmentation from the optical U-Net (orange) and manual segmentation from biological expert H2 (green). From the tested images, 52% fall into this range of JCD$\leq$ 0.1 and 67% when only spheroids beyond the standard size d_T > 400 µm are considered. Size of each image corresponds to 2650 µm × 2100 µm. Note that the U-Net is trained on an independent manual segmentations from another biological expert H1. Images are selected from the extended validation data set (Fig. 4), in particular, from the subset of larger, better discriminated spheroids at intermediate levels of debris for illustration. Note that in the example for JCD = 0.08, the two attached spheroids are correctly segmented, although the training data set does not contain such cases.

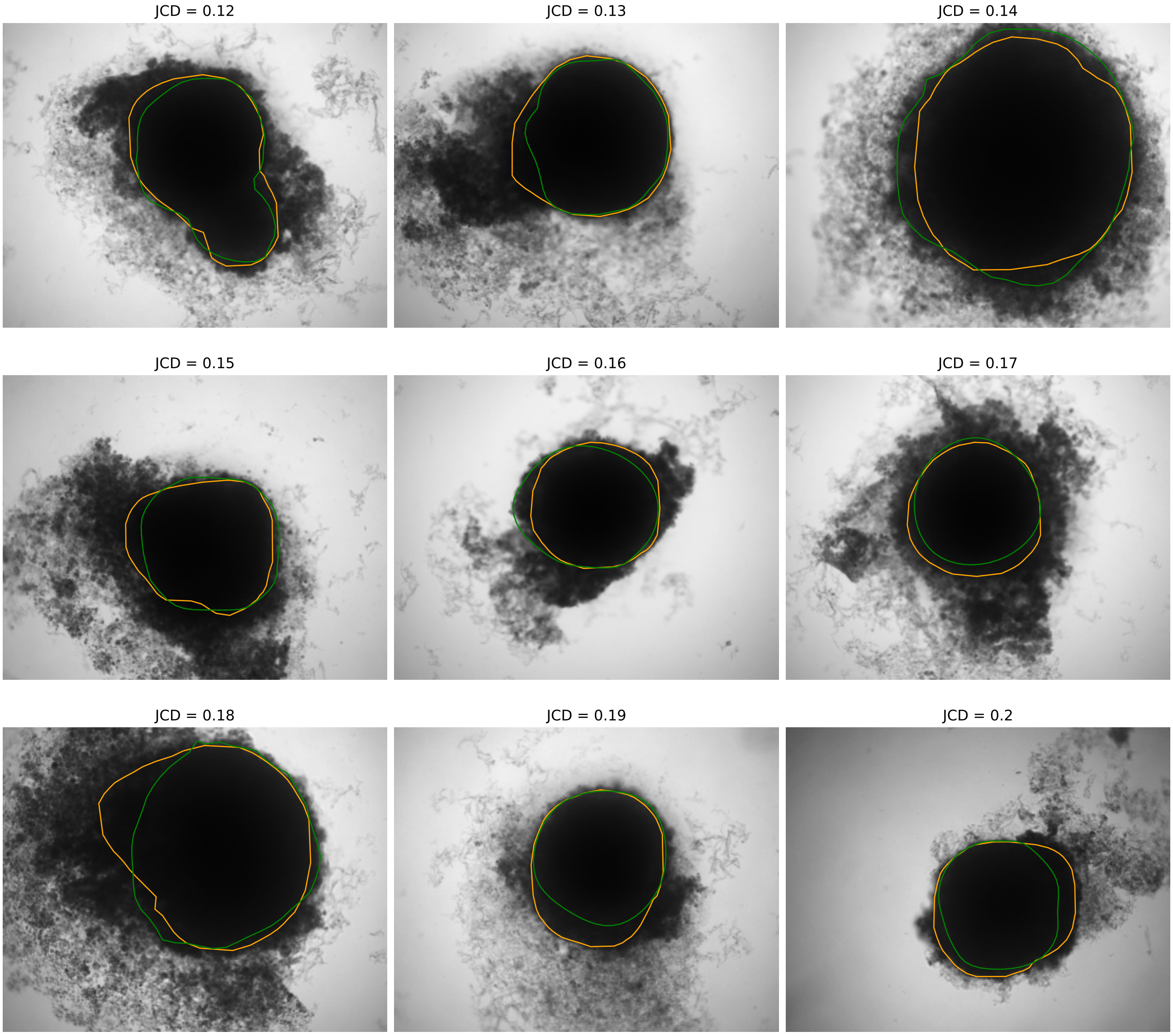

Figure SM.3. Selection of representative images as optical reference for 0.1 < JCD ≤ 0.2 with automatic segmentation from the optical U-Net (orange) and manual segmentation from biological expert H2 (green), analogous to Fig. SM.2. From the tested images, 22% fall into the range 0.1 < JCD ≤ 0.2 and 23% when only spheroids beyond the standard size $d_T > 400$ µm are considered. Note that in the example of JCD = 0.12, the two attached spheroids are correctly segmented, although the training data set does not contain such cases.

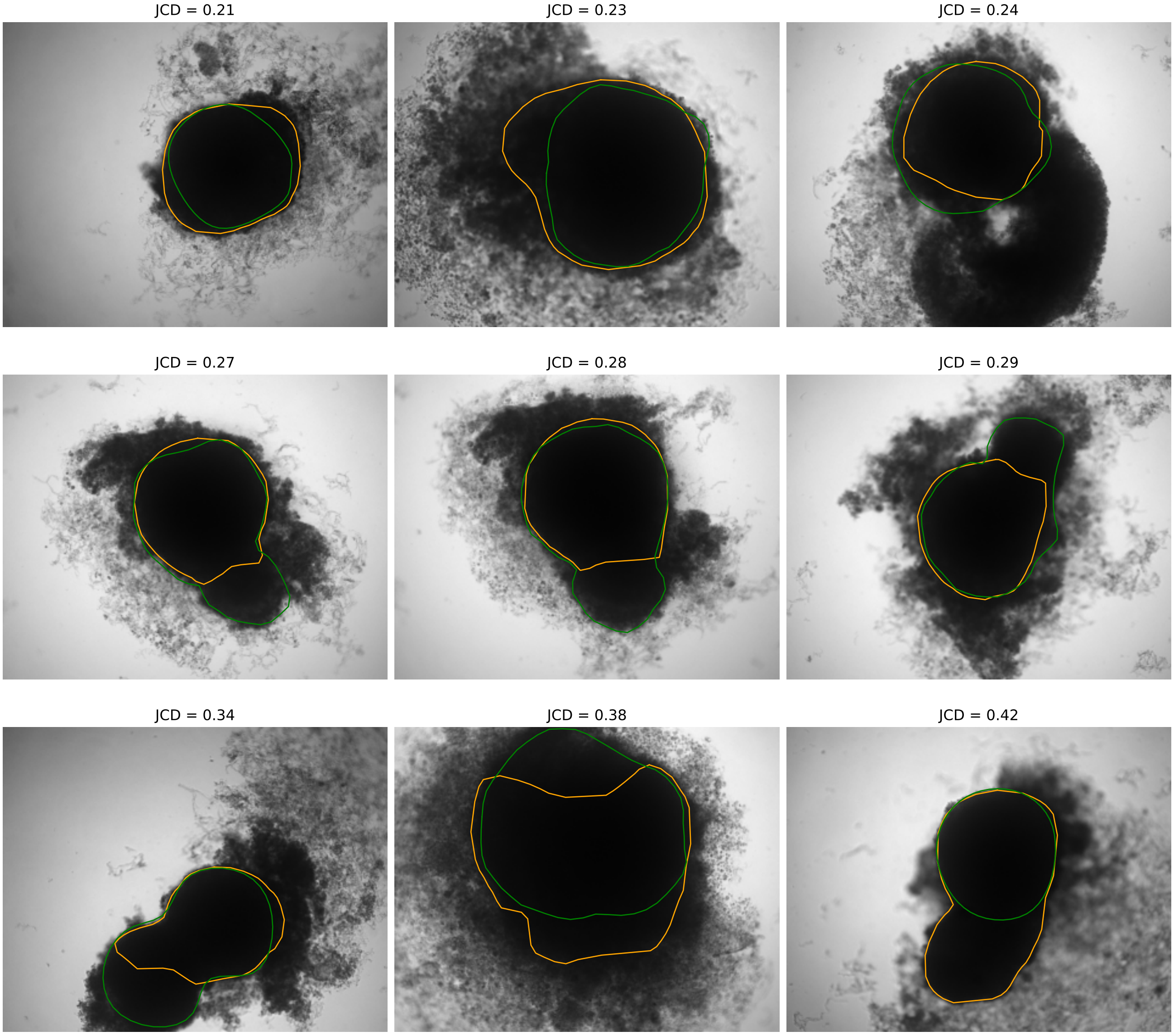

Figure SM.4. Selection of representative images as optical reference for larger deviations JCD $>$ 0.2 of the automatic segmentation from the optical U-Net (orange) and manual segmentation from biological expert H2 (green), analogous to Fig. SM.2. From the tested images, 26% fall into the range JCD $>$ 0.2, but merely 10% when only spheroids beyond the standard size $d_T > 400$ µm are considered. Note that deviations at larger spheroids are often due to cases of double-spheroids, e.g., JCD = 0.27, 0.28, 0.29, 0.34, 0.42, which are inconsistently recognized as either one or two spheroids even by the human.

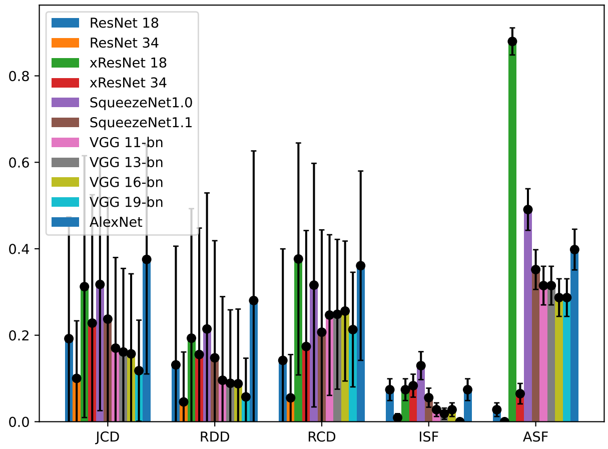

Backbone	JCD	RDD	RCD	ISF	ASF
ResNet 18	0.192	0.131	0.142	0.074	0.028
±	0.282	0.275	0.258	0.025	0.016
ResNet 34	**0.100**	**0.046**	**0.055**	0.009	**0.000**
±	0.133	0.116	0.100	0.009	0.000
xResNet 18	0.312	0.193	0.376	0.074	0.880
±	0.303	0.300	0.268	0.025	0.031
xResNet 34	0.228	0.155	0.174	0.083	0.065
±	0.297	0.292	0.268	0.027	0.024
SqueezeNet1.0	0.317	0.215	0.316	0.130	0.491
±	0.292	0.315	0.282	0.032	0.048
SqueezeNet1.1	0.237	0.148	0.207	0.056	0.352
±	0.281	0.271	0.237	0.022	0.046
VGG 11-bn	0.170	0.096	0.247	0.028	0.315
±	0.210	0.193	0.186	0.016	0.045
VGG 13-bn	0.161	0.089	0.248	0.019	0.315
±	0.193	0.170	0.173	0.013	0.045
VGG 16-bn	0.157	0.088	0.256	0.028	0.287
±	0.185	0.173	0.162	0.016	0.044
VGG 19-bn	0.118	0.057	0.213	**0.000**	0.287
±	0.117	0.090	0.133	0.000	0.044
AlexNet	0.375	0.280	0.361	0.074	0.398
±	0.265	0.346	0.219	0.025	0.047

Figure SM.5. Values of evaluation metrics for different backbones of the U-Net. Bold values highlight the optimum in each column. The other hyperparameters are fixed at Optimizer: Adam, Loss: Cross-Entropy, Resize factor: 1/2, Transfer learning: Yes, Data augmentation: No. Overall the ResNet 34 achieved the best results and is picked as the backbone for the U-Net.

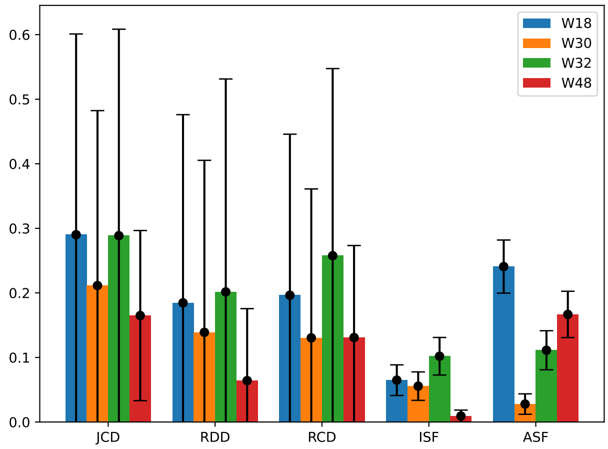

Backbone	JCD	RDD	RCD	ISF	ASF
W18	0.290	0.185	0.196	0.065	0.241
±	0.311	0.291	0.249	0.024	0.041
W30	0.211	0.139	**0.130**	0.056	**0.028**
±	0.271	0.266	0.231	0.022	0.016
W32	0.289	0.201	0.258	0.102	0.111
±	0.320	0.330	0.290	0.029	0.030
W48	**0.165**	**0.064**	0.131	**0.009**	0.167
±	0.132	0.111	0.143	0.009	0.036

Figure SM.6. Values of evaluation metrics for different backbones of the HRNet. Bold values highlight the optimum in each column. The other hyperparameters are fixed at Optimizer: Adam, Loss: Cross-Entropy, Resize factor: 1/2, Transfer learning: Yes, Data augmentation: No. Overall the W48 achieved the best results and is picked as the backbone for the HRNet.

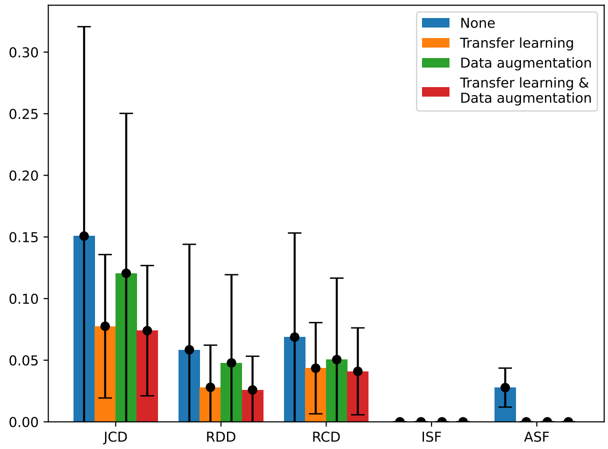

Extension	JCD	RDD	RCD	ISF	ASF
None	0.151	0.058	0.069	**0.000**	0.028
±	0.170	0.086	0.084	0.000	0.016
Transfer learning	0.078	0.028	0.043	**0.000**	**0.000**
±	0.058	0.034	0.037	0.000	0.000
Data augmentation	0.121	0.048	0.050	**0.000**	**0.000**
±	0.130	0.072	0.066	0.000	0.000
Transfer learning &	**0.074**	**0.026**	**0.041**	**0.000**	**0.000**
Data augmentation ±	0.053	0.027	0.035	0.000	0.000

Figure SM.7. Values of evaluation metrics for different extensions of the training data set, which is used by the U-Net. Bold values highlight the optimum in each column. The other hyperparameters are fixed at Backbone: ResNet 34, Optimizer: Adam, Loss: Cross-Entropy, Resize factor: 1/2. The accuracy of the U-Net is highest when transfer learning and data augmentation is used.

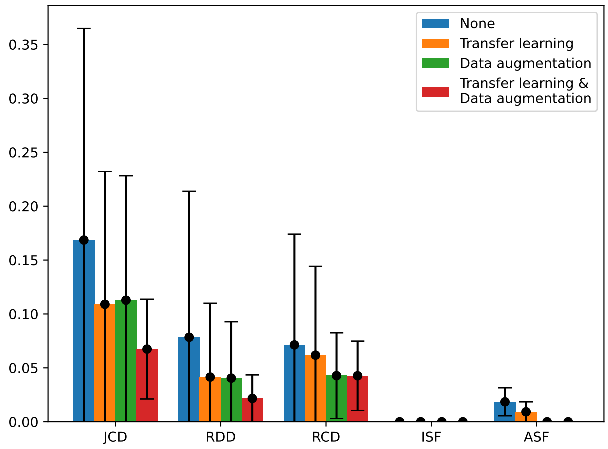

Extension	JCD	RDD	RCD	ISF	ASF
None	0.169	0.078	0.071	**0.000**	0.019
±	0.196	0.135	0.103	0.000	0.013
Transfer learning	0.109	0.041	0.062	**0.000**	0.009
±	0.123	0.069	0.082	0.000	0.009
Data augmentation	0.113	0.040	**0.043**	**0.000**	**0.000**
±	0.115	0.052	0.040	0.000	0.000
Transfer learning &	**0.067**	**0.022**	**0.043**	**0.000**	**0.000**
Data augmentation ±	0.046	0.022	0.032	0.000	0.000

Figure SM.8. Values of evaluation metrics for different extensions of the training data set, which is used by the HRNet. Bold values highlight the optimum in each column. The other hyperparameters are fixed at Backbone: W48, Optimizer: Adam, Loss: Cross-Entropy, Resize factor: 1/2. The accuracy of the HRNet is highest when transfer learning and data augmentation is used.

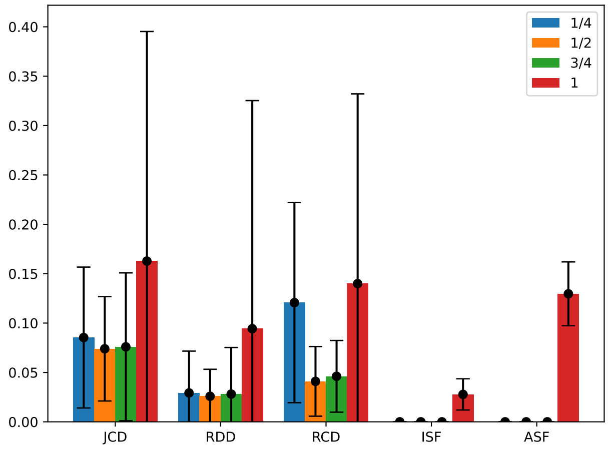

Resize factor	JCD	RDD	RCD	ISF	ASF
1/4	0.085	0.029	0.121	**0.000**	**0.000**
±	0.071	0.042	0.101	0.000	0.000
1/2	**0.074**	**0.026**	**0.041**	**0.000**	**0.000**
±	0.053	0.027	0.035	0.000	0.000
3/4	0.076	0.028	0.046	**0.000**	**0.000**
±	0.075	0.047	0.036	0.000	0.000
1	0.163	0.094	0.140	0.028	0.130
±	0.232	0.231	0.192	0.016	0.032

Figure SM.9. Values of evaluation metrics for different image sizes. Bold values highlight the optimum in each column. The other hyperparameters are fixed at Backbone: ResNet 34, Optimizer: Adam, Loss: Cross-Entropy, Transfer learning: Yes, Data augmentation: Yes. The U-Net can achieve the highest accuracy when the original image size is reduced by half.

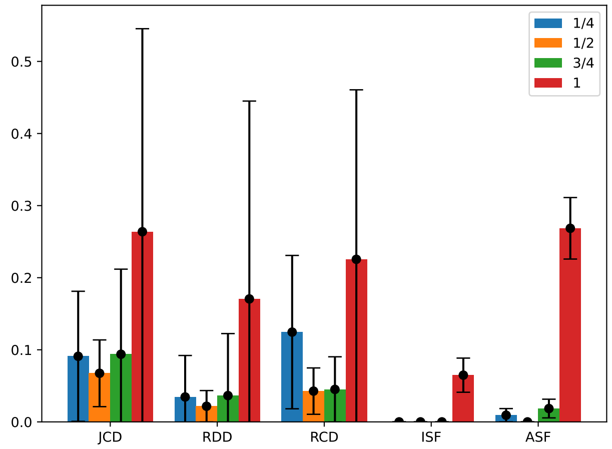

Resize factor	JCD	RDD	RCD	ISF	ASF
1/4	0.091	0.035	0.125	**0.000**	0.009
±	0.090	0.058	0.106	0.000	0.009
1/2	**0.067**	**0.022**	**0.043**	**0.000**	**0.000**
±	0.046	0.022	0.032	0.000	0.000
3/4	0.094	0.037	0.045	**0.000**	0.019
±	0.118	0.086	0.045	0.000	0.013
1	0.264	0.171	0.226	0.065	0.269
±	0.282	0.274	0.235	0.024	0.043

Figure SM.10. Values of evaluation metrics for different image sizes. Bold values highlight the optimum in each column. The other hyperparameters are fixed at Backbone: W48, Optimizer: Adam, Loss: Cross-Entropy, Transfer learning: Yes, Data augmentation: Yes. The HRNet can achieve the highest accuracy when the original image size is reduced by half.

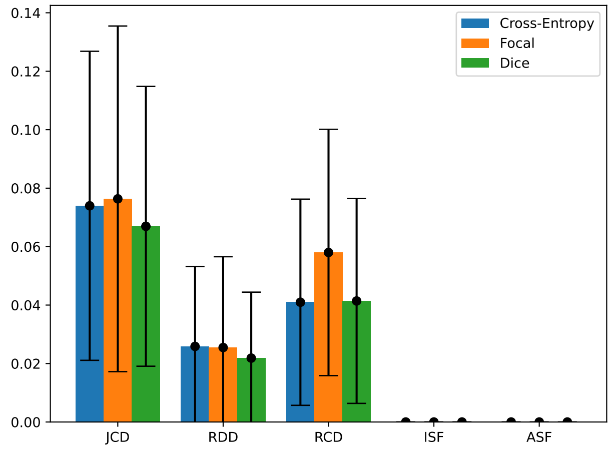

Loss function	JCD	RDD	RCD	ISF	ASF
Cross-Entropy	0.074	0.026	0.041	**0.000**	**0.000**
±	0.053	0.027	0.035	0.000	0.000
Focal	0.076	0.025	0.058	**0.000**	**0.000**
±	0.059	0.031	0.042	0.000	0.000
Dice	**0.067**	**0.022**	**0.041**	**0.000**	**0.000**
±	0.048	0.023	0.035	0.000	0.000

Figure SM.11. Values of evaluation metrics for different loss functions, used by the U-Net. Bold values highlight the optimum in each column. The other hyperparameters are fixed at Backbone: ResNet 34, Optimizer: Adam, Resize factor: 1/2, Transfer learning: Yes, Data augmentation: Yes. The U-Net can achieve the highest accuracy when the Dice loss is used.

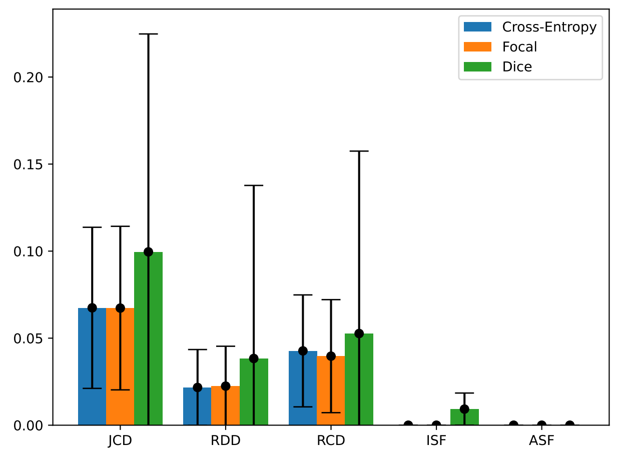

Loss function	JCD	RDD	RCD	ISF	ASF
Cross-Entropy	**0.067**	**0.022**	0.043	**0.000**	**0.000**
±	0.046	0.022	0.032	0.000	0.000
Focal	**0.067**	**0.022**	**0.040**	**0.000**	**0.000**
±	0.047	0.023	0.032	0.000	0.000
Dice	0.100	0.038	0.053	0.009	**0.000**
±	0.125	0.099	0.105	0.009	0.000

Figure SM.12. Values of evaluation metrics for different loss functions, used by the HRNet. Bold values highlight the optimum in each column. The other hyperparameters are fixed at Backbone: W48, Optimizer: Adam, Resize factor: 1/2, Transfer learning: Yes, Data augmentation: Yes. The HRNet can achieve the highest accuracy, if the Cross-Entropy or the Focal loss is used.

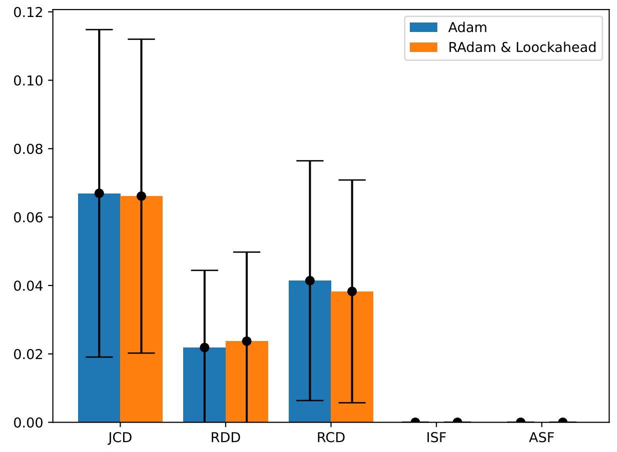

Optimizer function	JCD	RDD	RCD	ISF	ASF
Adam	0.067	**0.022**	0.041	**0.000**	**0.000**
±	0.048	0.023	0.035	0.000	0.000
RAdam & Lookahead	**0.066**	0.024	**0.038**	**0.000**	**0.000**
±	0.046	0.026	0.033	0.000	0.000

Figure SM.13. Values of evaluation metrics for different optimizers, used by the U-Net. Bold values highlight the optimum in each column. The other hyperparameters are fixed at Backbone: ResNet 34, Loss: Dice, Resize factor: 1/2, Transfer learning: Yes, Data augmentation: Yes. The U-Net can achieve the highest accuracy when the optimization is done by RAdam combined with Lookahead.

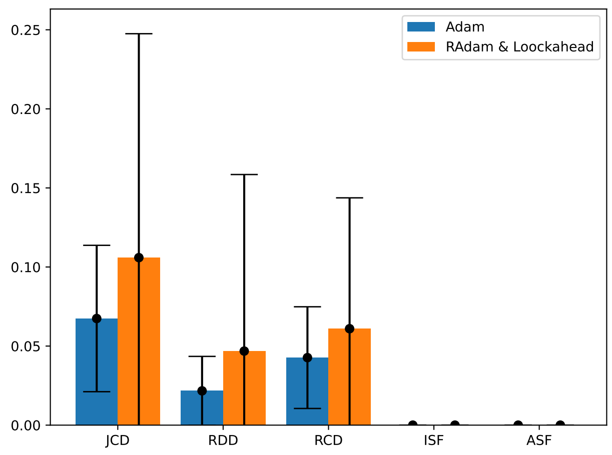

Optimizer function	JCD	RDD	RCD	ISF	ASF
Adam	**0.067**	**0.022**	**0.043**	**0.000**	**0.000**
±	0.046	0.022	0.032	0.000	0.000
RAdam & Lookahead	0.106	0.047	0.061	**0.000**	**0.000**
±	0.142	0.112	0.083	0.000	0.000

Figure SM.14. Values of evaluation metrics for different optimizers, used by the HRNet. Bold values highlight the optimum in each column. The other hyperparameters are fixed at Backbone: W48, Loss: Cross-Entropy, Resize factor: 1/2, Transfer learning: Yes, Data augmentation: Yes. The HRNet can achieve the highest accuracy when the optimization is done by Adam.

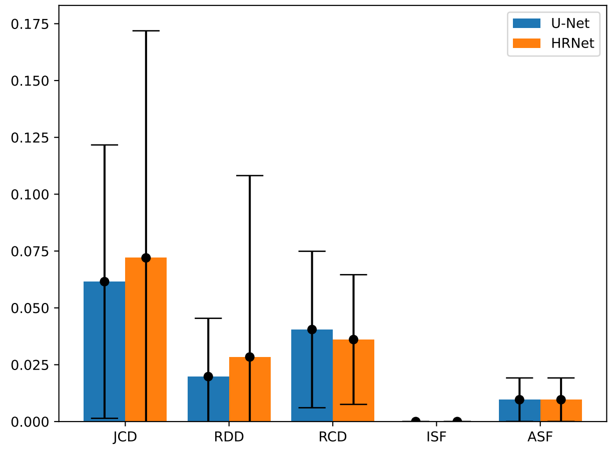

Model	JCD	RDD	RCD	ISF	ASF
U-Net	**0.062**	**0.020**	0.040	**0.000**	**0.010**
±	0.060	0.026	0.034	0.000	0.010
HRNet	0.072	0.028	**0.036**	**0.000**	**0.010**
±	0.100	0.080	0.029	0.000	0.010

Figure SM.15. Evaluation of the segmentation with the optimized U-Net and HRNet models on the test data set shows higher accuracy of the U-Net. The optimal hyperparameter configuration for the U-Net is Backbone: ResNet 34, Optimizer: RAdam & Lookahead, Loss: Dice, Resize factor: 1/2, Transfer learning: Yes, Data augmentation: Yes. The final hyperparameter configuration for the HRNet is Backbone: W48, Optimizer: Adam, Loss: Cross-Entropy, Resize factor: 1/2, Transfer learning: Yes, Data augmentation: Yes.

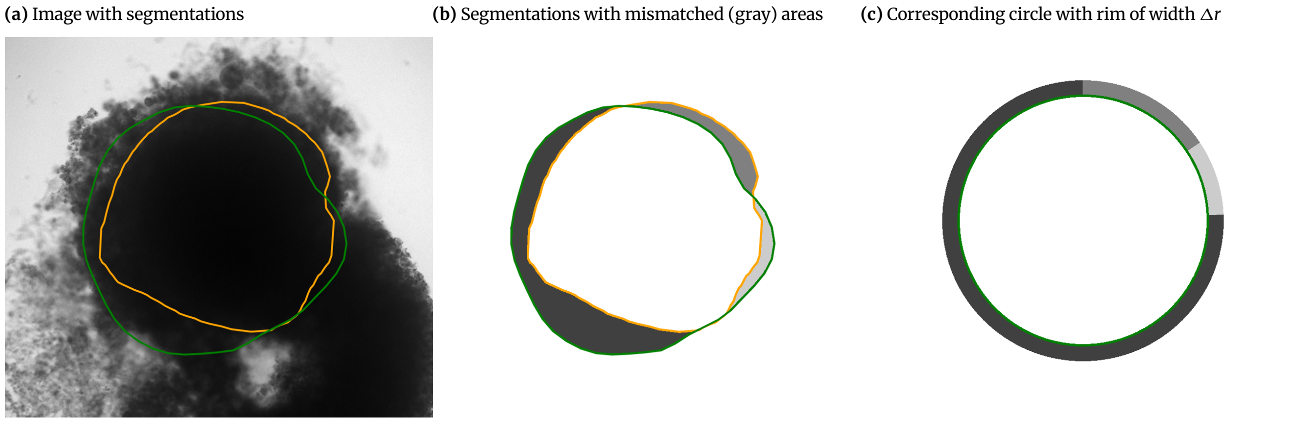

Figure SM.16. Illustration of the average radial error Δr defined in Eq. (3) (example with large JCD > 0.2 for visibility). (a) Zoom of image from Fig. SM.4 with JCD $= 0.24$ with corresponding automatic (orange line containing predicted area P) and manual segmentation (green line containing target area T). (b) Segmentations from (a) with mismatched areas shown in gray (missing area $T \setminus P$ in bright and dark gray, additional area $P \setminus T$ in intermediate gray). (c) Circle (green line) and added rim (gray areas) with areas corresponding to (b): Area within green circle is equal to area within manual segmentation in (b) and gray areas within the adjacent rim are equal to corresponding mismatched areas in (b). Then the radial thickness of the rim is equal to the average radial error Δr defined in Eq. (3).

(a) RDD over spheroid diameter

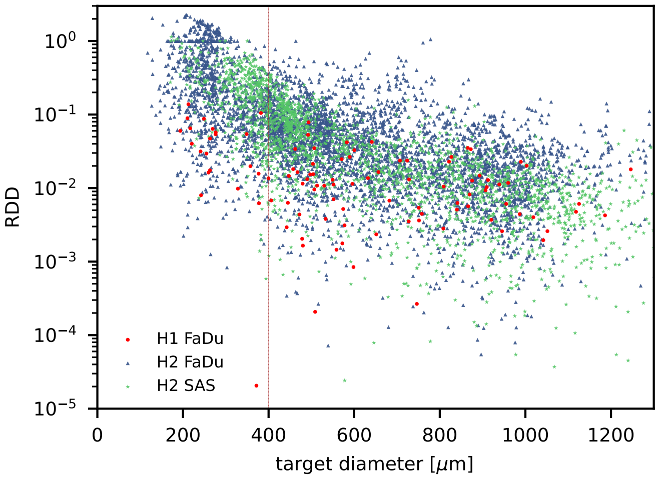

(b) Comparison diameters from manual and automatic segmentation

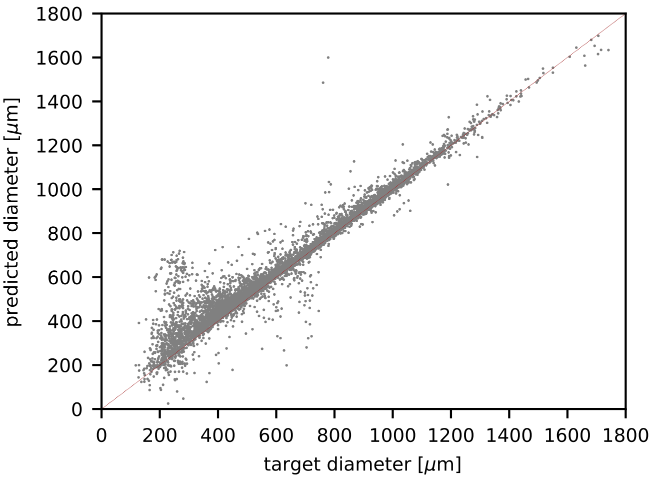

Figure SM.17. Validation based on diameter of automatic segmentation with the optimized U-Net on larger, independent data sets, analogous to Fig. 4. (a) RDD shows that majority of deviations are below 10% and higher deviations occur mostly below d_T < 400 μm. (b) Direct comparison of diameters (gray points) resulting from automatic segmentation (predicted diameter) and manual segmentation (target diameter) shows high accuracy (red line represents perfect match) with larger deviations mostly at d_T < 400 μm.

Manuscript with changes highlighted Click here to access/download;Manuscript;diff.pdf

GigaScience, 2024, 1–22

doi: xx.xxxx/xxxx
Manuscript in Preparation
Tech Note

TECH NOTE

Image segmentation of treated and untreated tumor spheroids by Fully Convolutional Networks

Matthias Streller[1,2], Soňa Michlíková[3,4], Willy Ciecior[1,2], Katharina Lönnecke[1,2], Leoni A. Kunz-Schughart[4,5], Steffen Lange[4,1,†,*] and Anja Voss-Böhme[1,2,*]

[1]DataMedAssist Group, HTW Dresden-University of Applied Sciences, 01069 Dresden, Germany
[2]Faculty of Informatics/Mathematics, HTW Dresden-University of Applied Sciences, 01069 Dresden, Germany
[3]Helmholtz-Zentrum Dresden-Rossendorf, Institute of Radiooncology - OncoRay, Dresden, Germany
[4]OncoRay - National Center for Radiation Research in Oncology, Faculty of Medicine and University Hospital Carl Gustav Carus, TUD Dresden University of Technology, Helmholtz-Zentrum Dresden-Rossendorf, Dresden, Germany
[5]National Center for Tumor Diseases Dresden (NCT/UCC), Germany: German Cancer Research Center (DKFZ), Heidelberg, Germany; Faculty of Medicine and University Hospital Carl Gustav Carus, TUD Dresden University of Technology, Dresden, Germany; Helmholtz-Zentrum Dresden-Rossendorf (HZDR), Dresden, Germany

†steffen.lange@tu-dresden.de
*These authors contributed equally to this work and share last authorship.

Abstract

Background Multicellular tumor spheroids (MCTS) are advanced cell culture systems for assessing the impact of combinatorial radio(chemo)therapy as they exhibit therapeutically relevant in-vivo-like characteristics from 3D cell-cell and cell-matrix interactions to radial pathophysiological gradients. State-of-the-art assays quantify long-term curative endpoints based on collected brightfield image time series from large treated spheroid populations per irradiation dose and treatment arm. This analyses require laborious spheroid segmentation of up to 100.000 images per treatment arm to extract relevant structural information from the images, e.g., diameter, area, volume and circularity. While several image analysis algorithms are available for spheroid segmentation, they all focus on compact MCTS with clearly distinguishable outer rim throughout growth. However, they often fail for the common case of treated MCTS, which may partly be detached and destroyed and are usually obscured by dead cell debris.
Results To address these issues, we successfully train two Fully Convolutional Networks, UNet and HRNet, and optimize their hyperparameters to develop an automatic segmentation for both untreated and treated MCTS. We extensively test the automatic segmentation on larger, independent data sets and observe high accuracy for most images with Jaccard indices around 90%. For cases with lower accuracy, we demonstrate that the deviation is comparable to the inter-observer variability. We also test against previously published datasets and spheroid segmentations.
Conclusions The developed automatic segmentation cannot only be used directly but also integrated into existing spheroid analysis pipelines and tools. This facilitates the analysis of 3D spheroid assay experiments and contributes to the reproducibility and standardization of this preclinical *in-vitro* model.

Key words: Spheroids, Organoids, Brightfield Microscopy, Segmentation, Deep Learning, Fully Convolutional Networks, High-content Screening, 3D Cancer Models, Cancer Therapy, Inter-observer Variability

Introduction

One of the most challenging problems in oncology is the development of therapeutic approaches for tumor growth suppression and eradication, as well as designing and optimizing treatment protocols. In this context, three-dimensional (3D) multicellular tumor spheroids (MCTS) are an advocated pre-clinical, in-vitro model to systematically study possible means of tumor suppression, assess the curative effect of combinatorial radio(chemo)therapy, and predict the response of *in-vivo* tumors [1–8]. In contrast to two-dimensional (2D) clonogenic survival assays, which are known to reflect the therapeutic response of cancer cells in the tissue comparatively poorly [9], multicellular spheroids are reproducible 3D avascular clusters of several thousand tumor cells without or in advanced settings with stromal cell compartments mimicking the pathophysiological milieu of tumor microareas or micrometastases. Due to their more or less radial 3D histomorphology and structure, they exhibit many characteristic features affecting tumor growth dynamics, including 3D reciprocal cell-cell and cell-

Compiled on: February 7, 2025.
Draft manuscript prepared by the author.

Key Points

- 3D tumor spheroids as advocated pre-clinical model lack reliable image segmentation.
- An automatic spheroid segmentation even for treated spheroids obscured by dead cell debris is developed.
- Segmentation is tested against independent datasets and compared to previous deep learning models.
- High-quality training dataset with challenging cases of treated spheroids is provided.
- Complementary software tool is released for high throughput image analysis of spheroids.

extracellular-matrix interactions as well as metabolic gradients of oxygen, nutrients and waste products, which strongly impair the cells' proliferative activity and therapy response [4–8], in particular oxygen deficiency (hypoxia), which is associated with substantial radioresistance [10–13]. After a long phase in which spheroids were used only in specialized laboratories, methodological advances in serial culturing and live imaging have led to an exponential spread of this in-vitro model system over the past two decades [7, 8, 14].

While 3D tumor spheroids provide a physiologically more realistic *in-vitro* framework to study tumor growth dynamics and therapeutic outcomes, the analysis of the experiments is much more complex than for traditional 2D cultures. 3D tumor spheroids' dynamics with and without treatment are most frequently monitored via microscopy imaging. State-of-the-art long-term spheroid-based assays assess therapy response from these image time series by classifying each spheroid within a population as either controlled or relapsed based on their growth kinetics. By averaging the therapeutic response over ensembles of spheroids for each treatment dose, the spheroid control probabilities and spheroid control doses are computed as analytical endpoints [15–18], analogous to the tumor control dose employed in *in-vivo* radiotherapy experiments with tumor-bearing mice [19, 20]. Moreover, time points of relapse are identified to quantify growth recovery in terms of Kaplan-Meier curves. These metrics depend on the spheroid type (cell line), the size of the spheroids at the onset of treatment, and the applied treatment and often require the extraction of growth curves, i.e., spheroid volume over time, from the image time series of all individual spheroids.

The associated data analysis poses a significant, interdisciplinary challenge: A single, typical experimental series of a long-term spheroid-based assay, i.e., 2-3 cell models at ten different doses with 3-5 agents and 30 spheroids per treatment arm monitored up to 60 days after treatment, generates up to 10^5 images. In the past, several tools were developed for spheroid image analysis [21–26], including SpheroidSizer [27], AnaSP [28, 29], TASI [30], SpheroidJ [31], and INSIDIA [25, 32]. The most crucial part of spheroid image analysis is the identification of the set of pixels in an image that corresponds to the spheroid, as this forms the basis for the extraction of spheroid characteristics like diameter, volume, and circularity as well as other morphologic features. This identification or classification of pixels in a given image is denoted semantic image segmentation [33] and represents the greatest challenge in spheroid image analysis. Previous spheroid segmentations were based on classical segmentation techniques, including thresholding methods (Yen [34], Otsu [28, 35]), watershed algorithm [36], shape-based detections (Hough transform algorithms [37], active contour models/snake [38]), or edge detection (Canny, Sobel [31]), and are also available as ImageJ plugins [22, 25], Matlab packages [27, 28, 30], or dedicated segmentation programs [39–41]. Since the characteristics of spheroid images, including the size, shape, and texture of the actual spheroid, vary with cell line, treatment, and microscopy method (e.g., brightfield [25, 29, 31, 32, 42], fluorescence [25, 26, 31, 32, 43, 44], differential, interference contrast [45]), most of these approaches and tools are specialized for specific experimental conditions or even selected microscopes [43, 44] and often fail to generalize to arbitrary conditions. In recent years, data-based methods, especially deep-learning models, have been increasingly employed for spheroid segmentation to tackle this issue of generalization [29, 31, 42, 45–49]. Deep-learning segmentation also allows more complex analysis, e.g., identification of time-lapse migratory patterns [45] or distinction of the spheroids' core and invasive edge [32].

However, these segmentation and analysis tools have primarily been developed for experimental conditions where the resulting images are relatively clean with well-visible, unobscured MCTS. In contrast, radiotherapy, one of the most common cancer treatments, regularly causes spheroids to shed dead cells or even to detach completely. Consequently, dead cell debris often obscures the remaining shrunk MCTS and the 3D cultures putatively regrowing from surviving viable cells after detachment, see Fig. 1 for two examples. This debris can cover a much larger domain than the actual spheroid and can be locally even thicker and thus darker, which makes segmentation difficult even for human experts. Both classical segmentation techniques and previous deep-learning models typically fail in these cases and require manual adjustments. We illustrate this challenge of spheroid segmentation by applying the four most recently proposed deep-learning models [29, 31, 48] to images with and without severe cell debris, see Fig. SM.1 for representative examples and Tab. SM.1 for statistical results.

We train deep-learning models to segment spheroid images after radiotherapy based on annotated images from a previous experiment assessing the radioresponse of human head-and-neck squamous cell carcinoma (HNSCC). After systematic optimization of hyperparameters and pre-processing for two selected network architectures, U-Net and HRNet, the automatic segmentation exhibits high accuracy for images of both treated and untreated spheroids. We further validate the automatic segmentation with the optimized U-Net on larger, independent data sets of two cell types of head-and-neck cancer exposed to both radiotherapy and hyperthermia. For the majority of images, we find excellent overlap between manual and automatic segmentation, even in the case of small, heavily obscured spheroids, see Fig. 1. In particular, we compare segmentations of different biological experts and find that imprecisions of the automatic segmentation are comparable to variations across different humans. The optimized automatic segmentation is provided in a minimal tool with a graphical user interface.

Materials

The original data is compiled from several data sets of time images of FaDu cell line spheroids, one of the two head-and-neck cancer cell types from the previously published study on combinatorial radioresponse in human head-and-neck squamous cell carcinoma spheroid models [16]: Spheroid popula-

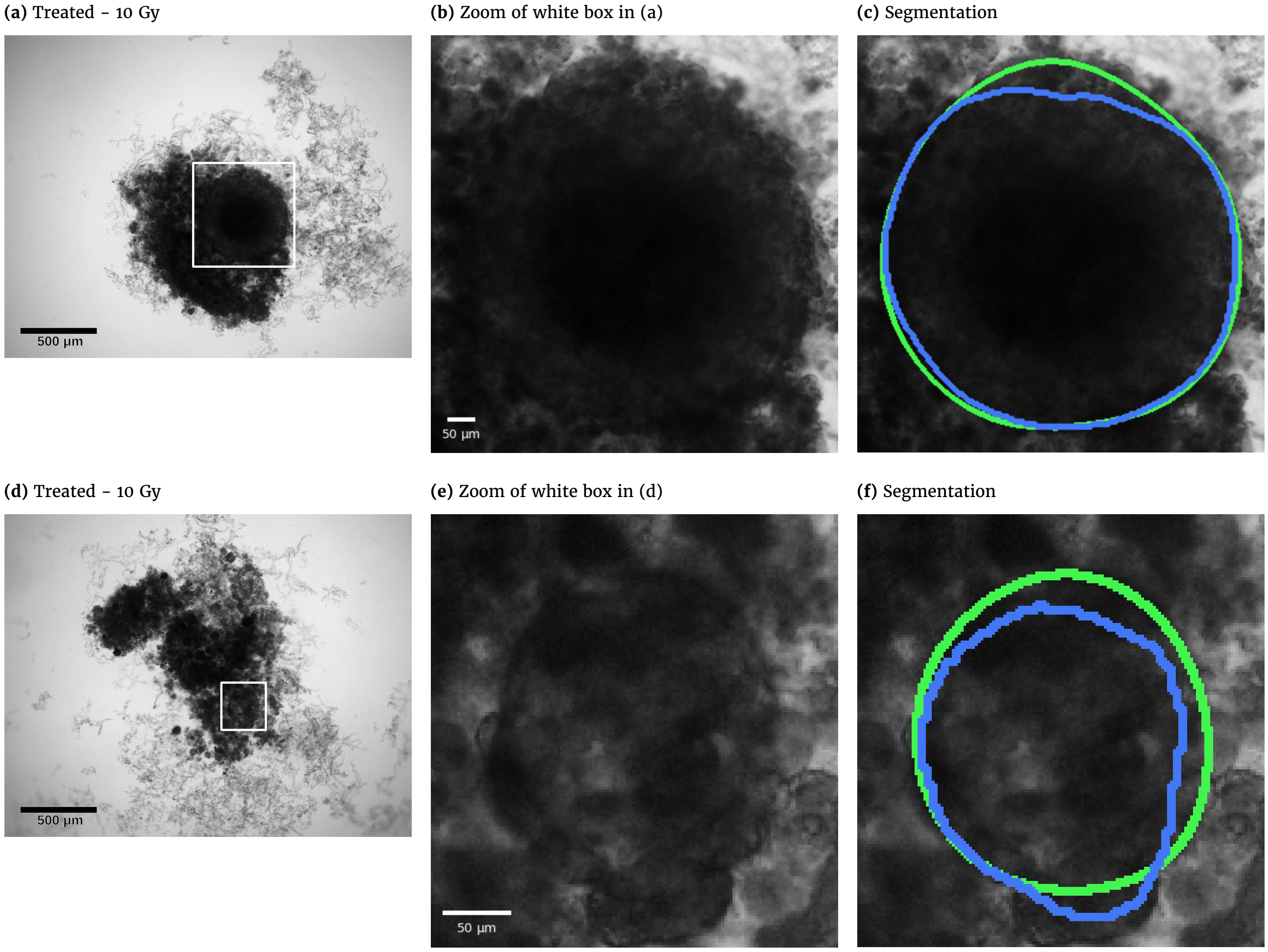

Figure 1. Representative examples of images for automatic segmentation with the optimized U-Net (blue) compared to the manual segmentation (green) for 3D tumor spheroids after treatment. The overlap with the manual segmentation is excellent for standard size and larger spheroids obscured by cell debris (top row) and sufficient for small, heavily obscured spheroids (bottom row). Displayed are (a,d) the original images, which are also the input for the U-Net, (b,d) magnified image details around the spheroids as indicated by the white box in (a,d) for visibility, and (c,e) corresponding contours from the segmentations. The metrics for evaluation of the automatic segmentation are: the top row - Jaccard Distance (JCD) = 7.6%, Relative Diameter Deviation (RDD) = 3.7% and Relative Circularity Deviation (RCD) = 1.6%; bottom row - JCD = 22.7%, RDD = 7.5% and RCD = 14.7%, see text for details.

tions were irradiated with X-rays at single doses of either 2.5 Gy, 5.0 Gy, 7.5 Gy or 10.0 Gy, with each dose being represented approximately equally in the data set. Spheroids were imaged for up to 60 days after treatment with brightfield microscopy, i.e., at different times after treatment and with potentially different lighting conditions. 1095 spheroids were manually segmented by a biological expert (human H1) to train and test the fully convolutional networks (FCNs). We use the train-validation-test-split technique for validation during training, which is appropriate for the large datasets and commonly used to train spheroid segmentation models [29, 31, 48]. We split the annotated data into 3 separate data sets: 883 images for training, 108 for validation, and 104 for testing (Hold-out test set). Images from a single spheroid are exclusively assigned to one of the groups to rule out unwanted correlations and data leakage between training, validation, and testing data sets. For further testing, another set of 6574 images of head-and-neck cancer (FaDu and SAS) spheroids from the same study [16], treated with different combinations and doses of X-ray irradiation and hyperthermia, is manually segmented by several independent biological experts (humans H2-H5). Note that while the automatic segmentation is performed on individual images, biological experts always take precedent and subsequent images of a spheroid into account for manual segmentation.

The original images were taken with a Zeiss Axiovert 200M at a resolution of 1300 × 1030 pixels, representing 2.04 µm/pixel with 16-bit gray-scale per pixel [16]. Images are converted to 8-bit images to ensure compatibility with the employed libraries FastAi [50] and SemTorch [51]. While this conversion, in principle, reduces the contrast level, the effect is negligible as only a small fraction of the 16-bit range is utilized during imaging, e.g., the mean gray-scale value in the images is 1300 ± 130, and values are rescaled such that smallest gray-scale value of the original image becomes 0 and the biggest value becomes 255.

As deep-learning models, we use FCNs, a particular group of convolutional neural networks intended for semantic image segmentation [52], in particular the U-Net [53] and the HR-Net [54]. The U-Net is one of the most established FCN frameworks. The HRNet has a special architecture compared to other typical FCN models and has already been successfully employed in the case of clean images with clearly visible spheroids [31]. The pipeline to train the models employs FastAi [50] for the U-Net and its backbones and SemTorch [51] for the HRNet. Before each training, a suitable learning rate is picked using LR Range [55]. The training follows the 1-cycle policy: the learning rate is cycled once between two bounds, allowing fast convergence [55]. During training we use validation-based early

stopping, where the JCD is the underlying metric. The models are trained on a GPU NVIDIA GeForce RTX 3080 with a mini-batch size of 2. For training on images with higher resolution or to compare some specific backbones, online learning is used instead of mini-batches to avoid memory overflow, i.e., the batch size, the amount of data used in each sub-epoch weight change, is reduced to one.

Methods

The segmentation is evaluated via several metrics (Section Evaluation metrics: Jaccard Distance (JCD), Relative Diameter Deviation (RDD), Relative Circularity Deviation (RCD), Ambiguous Spheroid Fraction (ASF), Invalid Spheroid Fraction (ISF)) additional to the standard accuracy quantified by the Jaccard coefficient of the whole data set, which is used during training. These metrics are computed for individual images to report not only their mean values but also their deviations, and thus reliability, across the test data set. These metrics are used to optimize the hyperparameters, Section Hyperparameter optimization , and data augmentation, Section Data Augmentation, of the selected FCN models U-Net and HRNet for maximal accuracy.

Evaluation metrics

Several metrics are employed to asses the accuracy of the segmentation. The most important one is the Jaccard Distance (JCD), which measures the relative difference between two sets of pixel P and T,

$$\mathrm{JCD} = 1 - \mathrm{IoU} = 1 - \frac{|P \cap T|}{|P \cup T|}. \quad (1)$$

with the automatically segmented (predicted) pixel set P from the FCN and the (target) pixel set T of the manually segmented spheroid. Note that in the literature often the opposite metric Intersection over Union IoU = 1 − JCD also called Jaccard index, or Jaccard similarity coefficient, is used. The JCD takes values between 0 (automatic segmentation perfectly overlaps with the manual one) and 1 (no intersection between the two segmentations). The JCD can be understood as the relative area error of the segmentation, i.e., in the example of Fig. 2a JCD = 0.042 is obtained, which means 4.2% error in the segmented area. A selection of sample images with different JCDs is displayed in Figs. SM.2-SM.4 to give an optical reference for this metric. From these sample images and in accordance with the biological experts, it may be concluded that a JCD below 0.2 is justifiable and such a deviation is observed between segmentations of different humans, see also Section Further independent validation .

It is possible that the spheroid is correctly segmented, but additionally another non-existent spheroid is detected, see Fig. 2b, where additionally to the manually segmented spheroid in the top right another contour in the bottom left is suggested by the automatic segmentation. In this case, the JCD would be below 1 but differ from 0. The fraction of these cases among all images are additionally denoted Ambiguous Spheroid Fraction (ASF). Furthermore, a JCD of 1 can mean that the spheroid is found in the wrong place or the spheroid is not found at all, see Fig. 2c. The fraction of these cases is denoted Invalid Spheroid Fraction (ISF).

In practice, only the average diameter $d_T = 2\sqrt{|T|/\pi}$ of the segmented spheroid is extracted to estimate the three-dimensional volume $\pi d_T^3/6$ of the spheroid under the assumption of a spherical shape. Additionally, the circularity $4\pi|T|/L^2$ of the spheroid is computed from its area $|T|$ and perimeter L to assess the validity of this assumption. Note that this is just a common assumption in the field, although even a circular projection does not imply a spherical shape. Thus, we also assess the automatic segmentation by the resulting error of these two values and measure the Relative Diameter Deviation (RDD) and Relative Circularity Deviation (RCD) between the segmentations

$$\mathrm{RDD}, \mathrm{RCD} = \frac{|c_P - c_T|}{c_T} \quad (2)$$

where c_P is either mean diameter or circularity based on the automatic segmentation and c_T is the corresponding characteristic calculated based on the manual segmentation. In the rare case that several spheroids are detected in an image, the largest one is chosen for computation of the JCD. This strategy is in accordance with experimental standard practice for single spheroid-based assays with curative analytical endpoints, as the largest regrowing spheroid is sufficient to detect relapse after treatment. Note that if there were several spheroids present and of interest, also all of them could be segmented. In the case of JCD = 1, which corresponds to an invalid spheroid, RDD and RCD are also set to 1. The combination of the JCD, ASF, ISF, RDD, and RCD quantify the accuracy of the segmentation

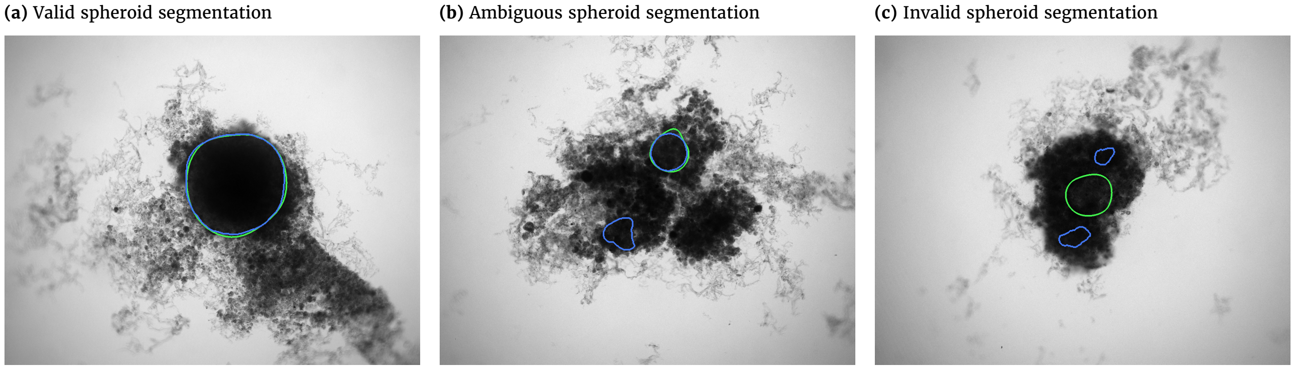

Figure 2. Exemplary evaluation of the automatic segmentation (blue) with respect to the manual one (green) for (a) standard case and (b),(c) rare artifacts, see text for details: (a) Correctly segmented spheroid: no contribution to Invalid Spheroid Fraction (ISF) or Ambiguous Spheroid Fraction (ASF); JCD, RDD and RCD are well defined. (b) Excessive spheroids are detected beyond the actual spheroid: no contribution to ISF, one count added to ASF; JCD, RDD and RCD are well defined and computed for the larger, upper spheroid. (c) No overlap between automatic and manual segmentation: one count added to ISF, no contribution to ASF; JCD, RDD and RCD are set to one.

with the FCN, where always a value closer to zero means higher agreement between the segmentations.

The spheroid's pixel set and perimeter are required to calculate the metrics. Thus, probability heatmaps, created by the Softmax function in the last layer of the FCN, are transformed into binary images and polygonal chains as illustrated in Fig. 3. First, the binary image is created. For this purpose, every pixel with a probability value higher or equal to 50% will be counted as a spheroid pixel and set to 1. Values of the other pixels are set to 0. The coordinates from the outer border of the emerged shape in the binary image can be extracted by the algorithm proposed in [56], thereby creating the final polygonal chain.

Hyperparameter optimization

Several hyperparameters and pre-processing methods can be adjusted to improve the accuracy of the selected FCN models. We successively optimize the most relevant parameters, starting from the ones with the most significant expected impact on accuracy to the one with the lowest impact, i.e., *backbone*, *transfer learning*, *data augmentation*, *input resolution*, *loss function*, and *optimizer*: The *backbone* defines the exact architecture of the FCN model and is accordingly the most important hyperparameter. For *transfer learning*, the ImageNet [57] data set is utilized. The *data augmentation* techniques are described in Section Data Augmentation. Furthermore, the impact of changing the *input resolution* is investigated. Afterward, the *loss function* for quantifying the FCN's error is optimized, particularly by comparing distribution-based loss and a region-based loss. We pick the Dice loss for region-based loss and the Cross-Entropy loss as a distribution-based loss function. We also test the Focal loss, which is a variation of Cross-Entropy and suitable for imbalanced class scenarios [33]. Finally, two different optimizers for minimizing the loss function are evaluated. One is Adam [58], a very common optimizer, and the other one is a combination of some more modern optimizers, RAdam [59] and Lookahead [60]. For the backbone, the optimization is initiated with a default setting, i.e., transfer learning, no data augmentation, input resolution half of original image, Cross-Entropy loss function, and Adam optimizer. These initial parameters are then individually optimized in consecutive order.

Data Augmentation

Data augmentation is a method to generate additional training data via transformations of the original data set. Since this increases the total amount of training data, data augmentation can improve performance and avoid overfitting [61]. In our case, every original training image is transformed once, doubling the size of the original training data set. For each image in the training data set, one of three transformations is picked randomly using Albumentations [61], i.e., vertical flip, horizontal flip, or rotation by 180°. These transformations are valid, since the spheroids do not have a specific orientation within an image. Note that each of these transformation conserves the original rectangular resolution of the image. This avoids extrapolating pixels on the border of the image, which would be necessary for arbitrary rotation angles and which can lead to additional image artifacts the FCN model would have to be trained for.

Results

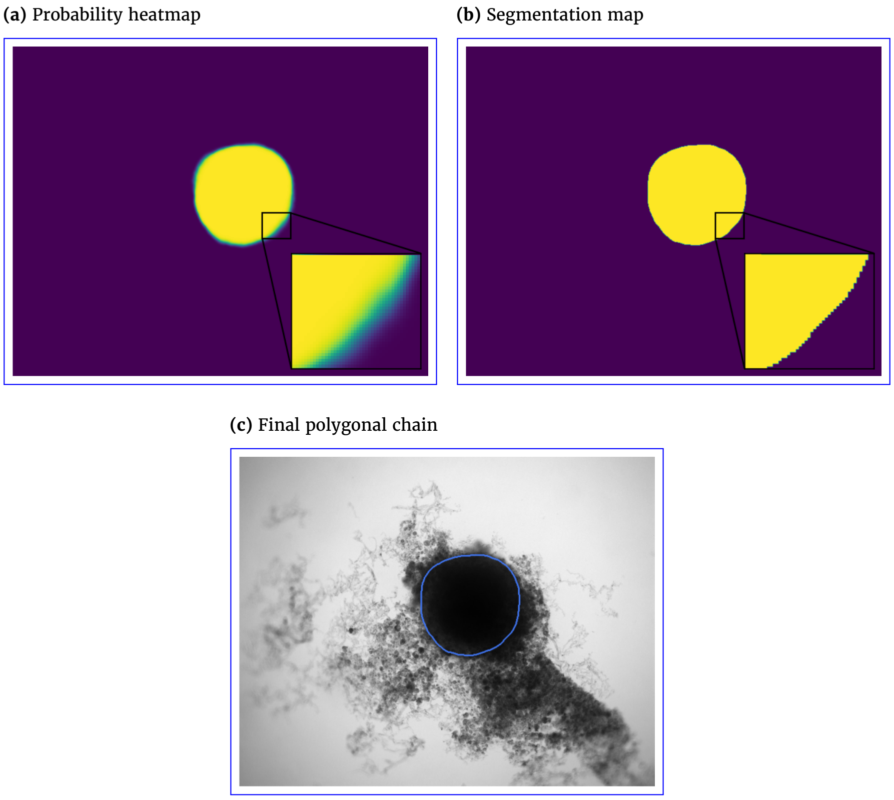

Figure 3. Post-processing steps for the output of the FCN model to transform probability heatmap to spheroid contour: (a) Probability heatmap as output of the FCN model. Each pixel takes probability values between 0 (black) and 1 (white) predicting the target (spheroid). Note that due to the steep gradient the gradual change from black to white is hard to see. (b) By using a threshold of 0.5, the pixels are classified into outside spheroid (black) and inside spheroid (white). (c) Contour of the spheroid border extracted as a polygonal chain (blue line displayed on original image).

Hyperparameter optimization

For each hyperparameter set and pre-processing method a model is trained and the performance of these trained models is compared for the validation data set based on the proposed evaluation metrics from Section Evaluation metrics. For JCD, RDD, and RCD not only the average value but also the standard deviation over the whole validation data set is reported to estimate reliability of the segmentation. For the ISF and the ASF the standard error of the mean of the corresponding binomial distribution is reported. All results of the optimization are listed in the supplemental material (Figs. SM.5-SM.14) and briefly discussed in the following.

Backbones

For the U-Net, the ResNet 34 and the VGG 19-bn architecture perform best, see Fig. SM.5, with a JCD three times smaller than the worst performing backbones, like AlexNet or SqueezeNet1.0. The two ResNet backbones stand out with a very low ASF. Note that the accuracy of the VGG architectures is improving with increasing number of used layers. The ResNet 34 is the optimal backbone as it not only performs best in virtually every metric, but also requires less computational time than the VGG 19-bn. Note that a previous study on deep-learning models for spheroid segmentation observed a higher performance for ResNet 18 compared to VGG 19 and ResNet 50 [29]. For the HRNet, the backbones differ in the number of kernels used and the accuracy increases with the number of kernels, see Fig. SM.6. The W48 backbone is preferred, as for the majority of metrics it performs best and has the lowest standard deviations.

Data augmentation and transfer learning

The combination of data augmentation and transfer learning leads to the best performance for both U-Net and HRNet, see Fig. SM.7 and Fig. SM.8. While this is in principle expected, the improvement is substantial as the JCD is more than halved when both transfer learning and data augmentation is introduced. Note that for the U-Net, transfer learning improves accuracy more than data augmentation, while the opposite is true for the HRNet.

Resolution

Using input images with half the resolution 650×515 of the original images leads to the best performance, see Fig. SM.9 and Fig. SM.10. This input resolution is already used to optimize the previous parameters. Higher resolution (3/4, 1) reduces accuracy, presumably because the increase in details makes generalization of the appearance of spheroids more difficult, and the receptive fields of the kernels are getting smaller relative to the whole image. Lower resolution (1/4) also reduces accuracy, presumably due to the loss of relevant information. Half resolution displays the best accuracy and lowest standard deviations across the validation data set.

Loss

We find the Dice loss to be the optimal loss function for the U-Net, see Fig. SM.11. Note that Dice is considered the default loss parameter when using the U-Net for cell segmentation, i.e., binary classification concerned with the accuracy of the edge. However, the advantage is much smaller than for previous hyperparameters. It does not suggest a general advantage of region-based loss for the segmentation of spheroids by the U-Net. For the HRNet, there is a more significant difference between the region-based loss and the distribution-based loss, see Fig. SM.12. The Focal loss and the Cross-Entropy loss perform better than the Dice loss - especially, the standard deviations differ by a factor of up to three. One reason for this behavior may be that the Dice loss provides less detailed feedback during training than the distribution-based losses. Cross-Entropy loss and Focal loss achieve nearly the same results, and we choose the default Cross-Entropy loss.

Optimizer

In case of the U-Net the difference between the tested optimizers is marginal, see Fig. SM.13. We choose the slighly better performing RAdam combined with Lookahead over the default optimizer Adam. In contrast, the accuracy of the HRNet is much worse for the combined optimizer, see Fig. SM.14. The Adam optimizer is optimal with evaluation metrics 1.5 times better than for the RAdam combined with Lookahead.

Table 1. (a) Evaluation of the segmentation with the optimized U-Net and HRNet models on the test data set shows higher accuracy of the U-Net. Note that all metrics can range between zero and one, where lower values mean higher accuracy and zero means perfect agreement with the manual segmentation. (b) Evaluation of the optimized segmentations on images of untreated (not irradiated) spheroids shows high accuracy also for the case of clean images without cell debris. The optimized U-Net and HRNet achieve almost equal accuracy. (c) The optimal hyperparameter configurations.

(a) Segmentation test data set

Model	JCD	RDD	RCD	ISF	ASF
U-Net	**0.062**	**0.020**	0.040	**0.000**	**0.010**
±	0.060	0.026	0.034	0.000	0.010
HRNet	0.072	0.028	**0.036**	**0.000**	**0.010**
±	0.100	0.080	0.029	0.000	0.010

(b) Segmentation images without cell debris

Model	JCD	RDD	RCD	ISF	ASF
U-Net	**0.028**	**0.008**	0.034	**0.000**	**0.000**
±	0.010	0.008	0.032	0.000	
HRNet	0.029	**0.008**	**0.033**	**0.000**	**0.000**
±	0.011	0.007	0.026	0.000	

(c) Optimized parameters

<table>
<tr><th>Parameter</th><th>U-Net</th><th>HRNet</th></tr>
<tr><td>Backbone</td><td>ResNet 34</td><td>W48</td></tr>
<tr><td>Transfer learning</td><td colspan="2">Yes</td></tr>
<tr><td>Data augmentation</td><td colspan="2">Yes</td></tr>
<tr><td>Resize factor</td><td colspan="2">1/2</td></tr>
<tr><td>Loss function</td><td>Dice</td><td>Cross-Entropy</td></tr>
<tr><td>Optimizer</td><td>RAdam & Lookahead</td><td>Adam</td></tr>
</table>

U-Net vs HRNet

The automatic segmentations with the final optimized U-Net and HRNet model are applied to the test data set. While this test data set is not taken into account during training of the models, the segmentations display virtually the same high accuracy on these new images as for the validation data set, see see Tab. 1a and Fig. SM.15 cf. Fig. SM.13 and Fig. SM.14. Mostly, the standard deviations of the metrics are higher in the test data set, though the JCD remains even with this deviation well below 20%. As for the validation data set, the U-Net performs slightly better than the HRNet on the test data. The JCD and RDD for the HRNet are 0.01 higher, and their standard deviation is 0.04 higher. Two representative examples of the automatic segmentation by the U-Net are shown in Fig. 1: The overlap with the manual segmentation is excellent for standard size and larger spheroids obscured by cell debris, see Fig. 1 top row, and sufficient for small, heavily obscured spheroids, see bottom row. In the latter case, the model does not segment the spheroid boundary accurately but detects the spheroid at the correct po-

sition JCD = 23% with a comparable size RDD = 7.5% within the much larger and often darker cloud of surrounding cell debris. In such difficult cases, similar variations are observed upon manual segmentation by different biological experts.

While the models are mainly trained on images with treated spheroids which are obscured by cell debris, they also accurately segment untreated spheroids with clear boundary and clean background, see Tab. 1b: On a test set of 100 images of untreated spheroids, the accuracy of both models is nearly the same with a JCD below 3%. In comparison, a recent deep-learning approach for spheroids with clear boundary and clean background with focus on generalizability to different experimental conditions and microscopes, achieved a mean JCD of 8% with a standard deviation of 12% for bright-field microscopic images [31]. This suggests that the standard case of clean images without cell debris is included in our training of images with heavily obscured spheroids.

The final U-Net model has a size of 158 MB and the final HRNet model a size of 251 MB. For both models, it takes about 1.8 seconds to segment one image on the CPU (Intel(R) Core(TM) i7-4770). When the segmentation is performed serially on the GPU (NVIDIA GeForce RTX 3080) the U-Net needs only 0.03 seconds per image and the HRNet 0.08 seconds. As computation time for both models is comparable, the U-Net is chosen for the automatic segmentation due to its slightly higher accuracy in out setting. Note that in a recently published deep-learning approach, the comparison of HRNet and U-Net implied that HRNet achieved the highest accuracy [31].

Further independent validation

In addition to the standard check with the Hold-out test data set, we systematically validate the automatic segmentation with the optimized U-Net on larger, independent Hold-out test data sets of two cell types of head-and-neck cancer, see Fig. 4. This data set contains 6574 images of FaDu or SAS spheroids treated with different combinations and doses of X-ray irradiation and hyperthermia [16]. These images were manually segmented by another biological expert (human H2) independent from the original training/validation/test-data set used to develop the automatic segmentation (human H1). For the majority of images, we find excellent overlap between this manual and the automatic segmentation, quantified by a JCD around 0.1, see Fig. 4a. Larger deviations are mostly observed for images with smaller spheroids, i.e., below the size of standard spheroids for treatment (diameter 370 – 400 μm according to [15–17]). To make the evaluation intuitive for the biological experts, who are less familiar with the JCD defined in Eq. (1), we additionally introduce a measure of the average radial error Δr based on the automatically segmented (predicted) domain P and the manually segmented (target) domain T

$$\Delta r = \sqrt{\pi^{-1}\left(|P \cup T| - |P \cap T|\right) + \frac{d_T^2}{4}} - \frac{d_T}{2} \tag{3}$$

with the average (target) diameter d_T of the manually segmented domain. The error Δr quantifies the thickness a circular layer with the size of the mismatched area $|P \cup T| - |P \cap T| = |P \setminus T| + |T \setminus P|$ around a circle with the target area $|T|$ would have, see Fig. SM.16 for an illustration. Since additional $P \setminus T$ and missing areas $T \setminus P$ do not compensate each other in Eq. (3), the error Δr is considerably larger than the radial error implied by the relative difference between target and predicted diameter RDD, see Fig. SM.17. The average radial error Δr reported in Fig. 4b assesses the segmentation analogous to the JCD with majority of errors $\Delta r < 20$ μm and larger deviations mostly for spheroids with diameter clearly smaller than before treatment

(a)

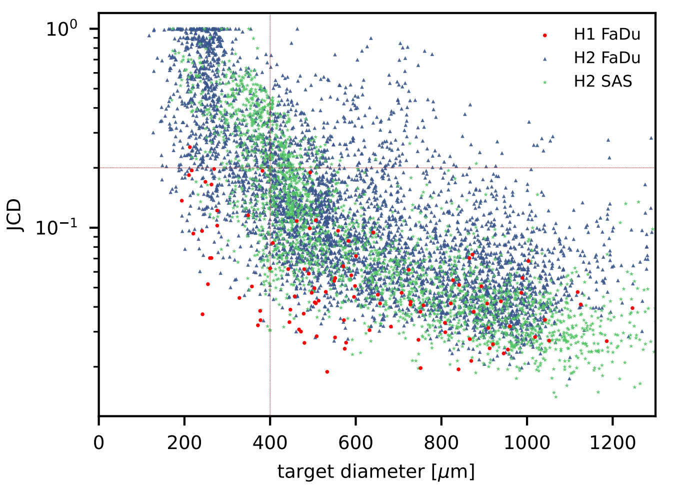

(b)

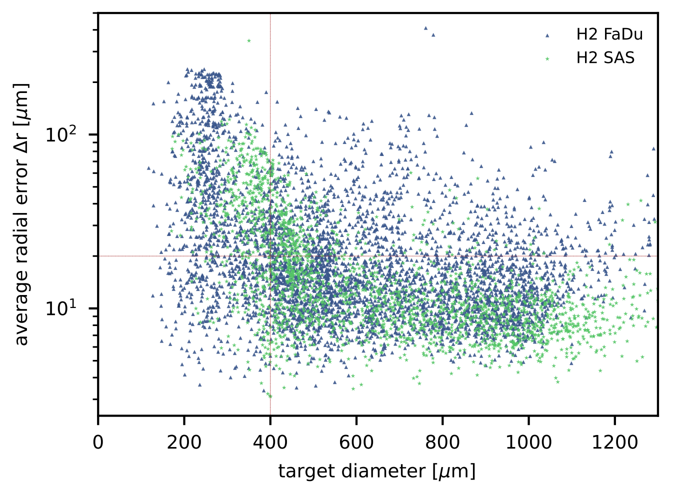

Figure 4. Validation of the automatic segmentation with the optimized U-Net on larger, independent data sets shows high accuracy for the majority of cases. (a) JCD and (b) average radial error Δr over diameter of the manually segmented (target) spheroid d_T for 6574 images of FaDu (blue triangles) and SAS (green stars) spheroids treated with different combinations and doses of X-ray irradiation and hyperthermia [16]. Manual segmentation is performed by a second biological expert (human H2, blue triangles and green stars) independently from the manual segmentation (human H1, red dots) of the training, validation and test data sets. (Results for 104 images of test data set is displayed as red dots for comparison.) Note that the segmentation is developed only based on images of FaDu spheroids. The majority of deviations are small (JCD < 0.2, Δr < 20 μm, red horizontal lines as guide to the eye), average (median) values are JCD = 0.17(0.09) ± 0.2, Δr = 25(15) ± 32 μm for the whole data set and JCD = 0.1(0.07) ± 0.09, Δr = 18(13) ± 17 μm for spheroids larger $d_T \geq 400$ μm (red vertical lines as guide to the eye) than the initial, standard size of spheroids. Larger imprecisions for smaller spheroids are due to biologically difficult, unclear, or ambiguous cases, see text.

($d_T \leq 370 - 400$ μm). For reference, note that the resolution of the images is 2.04 μm/px, i.e., a deviation of 20 μm corresponds to 10px (< 1% deviation with respect to the original 1300 × 1030 image) or 5px in the half-resolution image for application of the U-Net.

Only a few images with larger spheroids exhibit JCD bigger than 0.2. Most of these are ambiguous cases, with two spheroids attached to each other, which are inconsistently recognized as either one or two spheroids even by the human, see

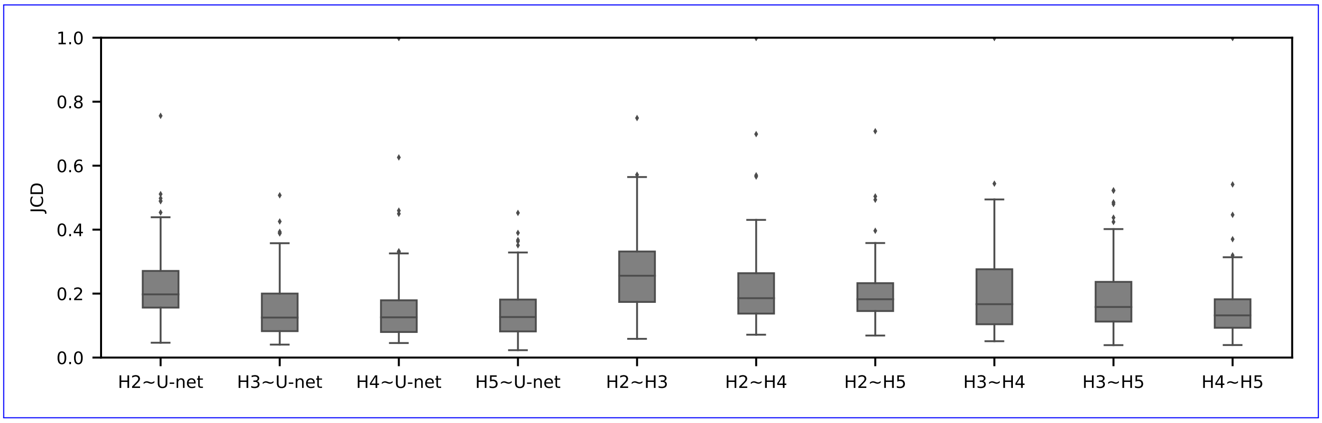

Figure 5. For treated spheroids smaller than the initial, standard size before treatment ($d \leq 400$ µm) deviations from the manual segmentation are not higher than variations across segmentations by different humans, suggesting that the segmentation of images with small spheroids surrounded by heavy debris is often difficult or ambiguous: Compared are segmentations from the optimized U-Net and 4 independent human experts (H2–H5) for the same 101 images, which are randomly selected from the pool of small spheroids of the extended Hold-out test data set, see Fig. 4. The Friedman test score of all JCDs 217.2 ($p \ll 0.001$) indicates significant differences among the pairwise segmentation deviations. The order according to the average JCD is (from low to high values) H5~U-Net, H3~U-Net, H3~H5, H2~H5 < H4~H5, H4~U-Net, H2~U-Net, H3~H4, H2~H4, H2~H3, where the < indicates a significant ($p < 0.005$) difference between the sets of JCDs according to a Dunn-Bonferroni pairwise post-hoc test.

examples with JCD = 0.28 – 0.34 and 0.42 in Fig. SM.4. While the training data set did not contain such double-spheroid cases, the optimized U-Net often segments them correctly, see examples with JCD = $0.08, 0.12$ in Figs. SM.2 and SM.3, respectively. Apart from this particular case, most images with larger deviations between the segmentations refers to smaller spheroids with surrounding cell debris. Note that smaller spheroids without cell debris, i.e., untreated or shortly after treatment, do not exhibit such deviations but are accurately segmented. To thoroughly examine these larger deviations in more detail, three biological experts independently segmented 101 randomly selected images with spheroid diameter $d_T \leq 400$ µm. For these 101 images the JCDs between the humans and the U-Net and between different humans are compared in Fig. 5. We find that on average the discrepancies between humans and U-Net are comparable to the variations across segmentations from different humans. This implies that these images represent biologically difficult, unclear, or ambiguous cases. It suggests that the larger JCD observed for some smaller spheroids rather reflects this uncertainty and not a low performance of the U-Net. Accordingly, the evaluation in Fig. 4 refers to a worse-case scenario, as for each spheroid, the biological expert intentionally segmented all images over time, while in practice, segmentation is only required and performed for a fraction of these images, in particular for clearly distinguishable and larger spheroids.

Finally, we also test the automatic segmentation with the optimized U-Net on eight published test data sets from previous deep-learning approaches [22, 29, 31], see Tab. SM.2. Note that the total of 496 images originates from different conditions, including brightfield and fluorescence microscopy, RGB and 16-bit gray-scale, and different microscopes, magnifications, image resolutions, and cell models, but do not contain significant cell debris. While our automatic segmentation performs well on roughly half of the data sets, sometimes surpassing the original model corresponding to the data set, two types of images turn out problematic: (i) images with ambiguous ground truth and (ii) images on which the spheroids appears semi-transparent, with individual cells being visible throughout the spheroid. However, classical segmentation techniques work sufficiently well for both types of images (i) and (ii), making the use of deep-learning approaches in these cases unnecessary. In detail, images of type (i) contain spheroids with peculiar intensity profiles, i.e., with a compact sphere-like core surrounded by a flat patch. The corresponding published ground truth just defines the outer boundary of these patches as the boundary of the spheroid, while our automatic segmentation restricts the spheroid to the compact core. Either choice may be unreasonable depending on the goal of the analysis. The outer boundary of the patches allows computation of the projected area of the spheroid while ignoring the considerable variations in thickness encoded in the intensity across the spheroid. Accordingly, it is not suited to estimate the spheroid volume necessary to evaluate volume growth. Presumably, the compact core contains most of the three-dimensional organized cells and cellular volume. It is the main component exhibiting metabolic gradients affecting therapy response [4–8], but certainly, it underestimates the total cell volume. Segmentation of such incompletely formed spheroids is debatable, even biologically, as the formation of proliferative and metabolic gradients remains ambiguous and may depend on the question pursued with the experiment. For images of type (ii), the apparent visibility of individual cells throughout the spheroid may be due to its small size or the chosen microscopy method.

Discussion

We develop an automatic segmentation for images of 3D tumor spheroids both with and without (radio)therapy. We systematically validate the automatic segmentation on larger, independent Hold-out test data sets of two cell types of head-and-neck cancer spheroid types, including combinations with hyperthermia treatment. For most images, we find excellent overlap between manual and automatic segmentation. These include clearly discernable spheroids and the previously neglected cases of spheroids critically obscured by cell debris. For images showing poor overlap of the segmentations, we demonstrate that this error is comparable to the variations between segmentations from different biological experts (inter-observer variability). This suggests that considerable deviations between automatic and manual segmentations do not necessarily reflect a low performance of the former but rather a general uncertainty or ambiguity in spheroid identification.

While the accuracy of (spheroid) segmentations is usually only quantified in terms of the JCD or Jaccard index (IoU), we choose to additionally report the corresponding implications on the spheroid diameter derived from the segmentation by Δr

and RDD. This makes the evaluation more intuitive for biological experts, as the spheroid diameter is the central metric for analysis. For instance, the spheroid volume required for growth curve documentation and growth delay is estimated from this diameter, which is connected to the projected area of the spheroid. We do not explicitly translate the evaluation to the corresponding spheroid area and volume. However, this may be estimated from basic scaling arguments, i.e., area and volume scale square and cubic with the diameter. The circularity of the spheroid, as quality control for its desired spherical shape, is taken into account by the RCD. All of these metrics will converge to their correct value, when the JCD goes to 0 (or Jaccard index to 1).

In fact, it should be pointed out that the imprecisions of the segmentation have a smaller effect on the quantification of therapy response than suggested by the reported JCD: As most informative parameters in long-term spheroid-based assays are the average diameters, volumes, and circularities of the spheroids. Thus, the relevant metric to estimate the error of the automatic analysis is not the JCD but the RDD and RCD, which are considerably smaller as even an imprecise segmentation can result in the correct size or shape of the spheroid. Furthermore, higher deviations are primarily observed for for treated 3D cultures with diameters below the initial diameter of standard spheroids $d_T = 370 - 400$ μm before treatment. These are usually images of spheroids in the final steps of detachment or before cell re-aggregation, growth recovery, and spheroid relapse. In experimental practice, such cases are typically not segmented as they are less relevant for the analytical endpoints. For instance, computation of growth delay requires accuracy of the spheroid segmentation immediately before treatment (for images without obscuring cell debris) and at large spheroid sizes $d_T > 600$ μm, while the images in between have no impact on the growth delay. Moreover, the validation in Fig. 4 is based on images from the time series of relapsed and controlled spheroids. However, in practical routine, segmentations of complete time series are only performed for growth curves and growth delay assessment in spheroid populations at 100 % growth recovery. The average JCD is substantially smaller if the validation is restricted to these cases.

It is important to note that the initial automatic segmentation is driven by images of only one HNSCC spheroid model (FaDu) with and without treatment. However, we show that it works equally well for spheroids from another cell type (SAS), although these SAS spheroids display a different peripheral shape during regrowth after treatment. Beyond the systematic validation highlighted herein, the automatic segmentation has been continuously tested and applied by several biological/biomedical experts and researchers for over a year during their ongoing experiments and to retrospectively reanalyze MCTS from earlier studies. So far, it has been reported that resegmentation is only necessary in a small fraction of cases, mainly due to optical artifacts, like out-of-focus images. Overall, the segmentation has in the meantime been successfully applied to numerous untreated multicellular spheroid types of different tumor entities and cell lines, respectively (FaDu, SAS - head and neck; Panc-02(mouse), Panc-1, PaTu 8902 - pancreas; HCT-116, HT29 - colon; A549, NCI-H23, NCI-H460 - lung; BT474 - breast; LNCap, DU145 - prostate; U87-MG, U138-MG, U251-MG - brain/GBM; Hek293 - kidney). These spheroids ranged between 200 – 1000 μm in diameter and images were taken at different magnifications and resolutions (e.g., 1300 × 1030, 1388 × 1040 1920 × 1440, 1920 × 1216 and 1.6 – 2.6 μm/pixel) as single or Z-stack images at diverse microscopic devices (Axiovert 200M, AxioObserver Z1 - both from Zeiss; BioTek Cytation 5 Cell Imaging Multimode Reader - Agilent). Many more spheroid types are in the pipeline for implementation. The developed automatic segmentation has also already been applied to contour the images of selected spheroid types, e.g., FaDu, SAS, Panc-02 or DU145, after various treatments such as radiotherapy (X-ray, proton), hyperthermia, chemotherapy and combinatorial treatment.

We also test the automatic segmentation on eight published test data sets from previous deep-learning approaches [22, 29, 31]. We observe a high accuracy, except for special cases, which, however, turn out to be well segmentable by classical techniques. While the automatic segmentation has been continuously tested for over a year on different cell lines, microscopes, and treatments, it does not necessarily generalize to arbitrary experimental conditions and imaging, which will be the focus of future improvements. However, this problem of domain shift, which can always arise when a model is applied to data sets with a different data distribution than the training data, is also an ongoing challenge for deep-learning models focused on images with clear spheroids and clean background [49]. In contrast, the focus of this study is the inclusion of the case of treated spheroids surrounded by severe cell debris, which is frequent after treatment but often neglected in automatic segmentation.

Note that the automatic segmentation's accuracy and reliability may not be exclusive to the finally chosen network architecture and hyperparameters. Indeed, we find that two very different network architectures, U-Net and HRNet, achieve very similar performance, which is consistent with previously reported optimizations of deep-learning models [31, Tab. 2] [29, Tab. 3]. The segmentation's accuracy also seems relatively insensitive to the choice of several hyperparameters, e.g., loss function, optimizer function, and, to some extent, the resize factor. Instead, it is plausible, that applying challenging training data, i.e., images with extensive, severe debris, is crucial for the performance of the resulting automatic segmentation. The network trained primarily on such images also works on images with clearly visible spheroids. Hence, the data sets compiled and annotated for this work are also publicly provided to support future improvements of spheroid segmentations. This data set can facilitate the development of more individual, more customized models, e.g., using the recently introduced nnU-Net tool [62].

The automatic segmentation can be incorporated into existing or future tools for spheroid analysis or medical image analysis as basis for further feature extraction including perimeter, complexity, and multiparametric analysis [47]. The segmentation provides a basis for the development of an automatic classification of spheroid image time series into control and relapse and could support machine learning methods to forecast tumor spheroid fate early. To make the deep-learning model available, we provide a minimal tool with graphical user interface. The ONNX Runtime [63] is used to compile the model and to take it into production.

Availability of source code and requirements (optional, if code is present)

- Project name: Spheroidsegdedeb (Spheroid segmentation despite debris)
- Project home page: `https://igit.informatik.htw-dresden.de/aagef650/spheroidsegdedeb`
- Operating system(s): Platform independent
- Programming language: Python >3.8.10
- Other requirements: Additionally download the model from `https://wwwpub.zih.tu-dresden.de/~s6079592/SEGMODEL.onnx`. Requirements and usage are documented in the provided README and some example images for testing are included in the corresponding folder.
- License: GNU GPL

Data availability

Training, validation, and test data used for this work are available ~~(before publication of the manuscript exclusively for reviewer:~~ at https://wwwpub.zih.tu-dresden.de/~s6079592/data.zip ~~)~~ and https://wwwpub.zih.tu-dresden.de/~s6079592/extended_data.zip as well as the code to train and evaluate the model (see above repo training_scripts/). All networks optimized for different hyperparameters are available in the .pth format at https://wwwpub.zih.tu-dresden.de/~s6079592/models/.

Declarations

List of abbreviations

Ambiguous Spheroid Fraction (ASF), fully convolutional network (FCN), Invalid Spheroid Fraction (ISF), Jaccard Distance (JCD), Multicellular tumor spheroids (MCTS), Relative Circularity Deviation (RCD), Relative Diameter Deviation (RDD), Supplemental material (SM)

Competing Interests

The authors declare that they have no competing interests.

Funding

The authors acknowledge that this research has been co-financed by the EU, the European Social Fund (ESF), and by tax funds on the basis of the budget passed by the Saxon state parliament (project SAB-Nr. 100382145) and the Bundesministerium für Bildung und Forschung (BMBF 16dkwn001a/b). The funders had no role in study design, data collection and analysis, decision to publish, or preparation of the manuscript.

Author's Contributions

Conceptualization, A.V.-B., L.A.K.-S., and S.L.; methodology, A.V.-B., S.L., S.M., L.A.K.-S., and M.S..; software, M.S., K.L., and S.L.; validation, W.C., K.L., S.M., and S.L.; formal analysis, M.S., A.V.-B., and S.L.; investigation, M.S., K.L., and S.L.; resources, L.A.K.-S., and A.V.-B.; data curation, M.S., W.C., K.L., and S.L.; writing—original draft preparation, M.S., A.V.-B. and S.L.; writing—review and editing, A.V.-B., W.C., S.L., S.M., and L.A.K.-S.; visualization, M.S., W.C., and S.L.; supervision, L.A.K.-S., A.V.-B. and S.L.; project administration, L.A.K.-S. and A.V.-B.; funding acquisition, L.A.K.-S. and A.V.-B. All authors have read and agreed to the published version of the manuscript.

Acknowledgements

We are grateful to M. Wondrak, S. Al-Jamei, N. El-Refai, R. Joseph, and S. L. Prieto for sharing their expertise on tumor spheroid contouring.

References

1. Franke F, Michlíková S, Aland S, Kunz-Schughart LA, Voss-Böhme A, Lange S. Efficient Radial-Shell Model for 3D Tumor Spheroid Dynamics with Radiotherapy. Cancers 2023;15(23):5645. https://www.mdpi.com/2072-6694/15/23/5645.
2. Brüningk SC, Ziegenhein P, Rivens I, Oelfke U, ter Haar G. A cellular automaton model for spheroid response to radiation and hyperthermia treatments. Sci Rep 2019;9(1):17674. https://www.nature.com/articles/s41598-019-54117-x.
3. Brüningk SC, Rivens I, Box C, Oelfke U, ter Haar G. 3D tumour spheroids for the prediction of the effects of radiation and hyperthermia treatments. Sci Rep 2020;10(1):1653. https://www.nature.com/articles/s41598-020-58569-4.
4. Riffle S, Hegde RS. Modeling tumor cell adaptations to hypoxia in multicellular tumor spheroids. Journal of Experimental & Clinical Cancer Research 2017;36(1):102. https://doi.org/10.1186/s13046-017-0570-9.
5. Leek R, Grimes DR, Harris AL, McIntyre A. Methods: Using Three-Dimensional Culture (Spheroids) as an In Vitro Model of Tumour Hypoxia. In: Koumenis C, Coussens LM, Giaccia A, Hammond E, editors. Tumor Microenvironment Advances in Experimental Medicine and Biology, Springer International Publishing; 2016. p. 167–196.
6. Hirschhaeuser F, Menne H, Dittfeld C, West J, Mueller-Klieser W, Kunz-Schughart LA. Multicellular tumor spheroids: An underestimated tool is catching up again. Journal of Biotechnology 2010;148(1):3–15. https://www.sciencedirect.com/science/article/pii/S0168165610000398.
7. Friedrich J, Ebner R, Kunz-Schughart LA. Experimental anti-tumor therapy in 3-D: Spheroids – old hat or new challenge? International Journal of Radiation Biology 2007;83(11):849–871. https://doi.org/10.1080/09553000701727531.
8. Kunz-Schughart LA, Freyer JP, Hofstaedter F, Ebner R. The Use of 3-D Cultures for High-Throughput Screening: The Multicellular Spheroid Model. J Biomol Screen 2004;9(4):273–285. https://doi.org/10.1177/1087057104265040.
9. Pérez-García VM, Calvo GF, Bosque JJ, León-Triana O, Jiménez J, Pérez-Beteta J, et al. Universal scaling laws rule explosive growth in human cancers. Nature Physics 2020;p. 1–6. https://www.nature.com/articles/s41567-020-0978-6.
10. Howard-Flanders P, Alper T. The Sensitivity of Microorganisms to Irradiation under Controlled Gas Conditions. Radiat Res 1957;7(5):518–540. https://www.jstor.org/stable/3570400.
11. Muz B, Puente Pdl, Azab F, Azab AK. The role of hypoxia in cancer progression, angiogenesis, metastasis, and resistance to therapy. Hypoxia med j 2015 Dec;3:83–92.
12. Rakotomalala A, Escande A, Furlan A, Meignan S, Lartigau E. Hypoxia in Solid Tumors: How Low Oxygenation Impacts the "Six Rs" of Radiotherapy. Front Endocrinol 2021;12. https://www.frontiersin.org/articles/10.3389/fendo.2021.742215.
13. Li C, Wiseman L, Okoh E, Lind M, Roy R, Beavis AW, et al. Exploring hypoxic biology to improve radiotherapy outcomes. Expert Rev Mol Med 2022 Jan;24:e21. https://www.cambridge.org/core/journals/expert-reviews-in-molecular-medicine/article/abs/exploring-hypoxic-biology-to-improve-radiotherapy-outcomes/A367ECCBE52B8E6D5D5B81FAC7C92B30.
14. Peirsman A, Blondeel E, Ahmed T, Anckaert J, Audenaert D, Boterberg T, et al. MISpheroID: a knowledgebase and transparency tool for minimum information in spheroid identity. Nat Methods 2021;18(11):1294–1303. https://www.nature.com/articles/s41592-021-01291-4.

15. Chen O, Manig F, Lehmann L, Sorour N, Löck S, Yu Z, et al. Dual role of ER stress in response to metabolic co-targeting and radiosensitivity in head and neck cancer cells. Cell Mol Life Sci 2021;78(6):3021–3044. https://doi.org/10.1007/s00018-020-03704-7.
16. Chen O, Michlíková S, Eckhardt L, Wondrak M, De Mendoza AM, Krause M, et al. Efficient Heat Shock Response Affects Hyperthermia-Induced Radiosensitization in a Tumor Spheroid Control Probability Assay. Cancers 2021;13(13):3168. https://www.mdpi.com/2072-6694/13/13/3168.
17. Hinrichs CN, Ingargiola M, Käubler T, Löck S, Temme A, Köhn-Luque A, et al. Arginine Deprivation Therapy: Putative Strategy to Eradicate Glioblastoma Cells by Radiosensitization. Mol Cancer Ther 2018;17(2):393–406. https://mct.aacrjournals.org/content/17/2/393.
18. Vynnytska-Myronovska B, Bobak Y, Garbe Y, Dittfeld C, Stasyk O, Kunz-Schughart LA. Single amino acid arginine starvation efficiently sensitizes cancer cells to canavanine treatment and irradiation. International Journal of Cancer 2012;130(9):2164–2175. https://onlinelibrary.wiley.com/doi/abs/10.1002/ijc.26221.
19. Kummer B, Löck S, Gurtner K, Hermann N, Yaromina A, Eicheler W, et al. Value of functional in-vivo endpoints in preclinical radiation research. Radiotherapy and Oncology 2021;158:155–161. https://www.sciencedirect.com/science/article/pii/S0167814021060874.
20. Baumann M, Petersen C, Krause M. TCP and NTCP in preclinical and clinical research in Europe. Rays 2005;30(2):121–126.
21. Hoque MT, Windus LCE, Lovitt CJ, Avery VM. PCaAnalyser: A 2D-Image Analysis Based Module for Effective Determination of Prostate Cancer Progression in 3D Culture. PLoS ONE 2013;8(11):e79865. https://journals.plos.org/plosone/article?id=10.1371/journal.pone.0079865.
22. Ivanov DP, Parker TL, Walker DA, Alexander C, Ashford MB, Gellert PR, et al. Multiplexing Spheroid Volume, Resazurin and Acid Phosphatase Viability Assays for High-Throughput Screening of Tumour Spheroids and Stem Cell Neurospheres. PLoS ONE 2014;9(8):e103817. https://journals.plos.org/plosone/article?id=10.1371/journal.pone.0103817.
23. Monjaret F, Fernandes M, Duchemin-Pelletier E, Argento A, Degot S, Young J. Fully Automated One-Step Production of Functional 3D Tumor Spheroids for High-Content Screening. SLAS Technology 2016;21(2):268–280. https://www.sciencedirect.com/science/article/pii/S247263032201384X.
24. Rueden CT, Schindelin J, Hiner MC, DeZonia BE, Walter AE, Arena ET, et al. ImageJ2: ImageJ for the next generation of scientific image data. BMC Bioinformatics 2017;18(1):529. https://doi.org/10.1186/s12859-017-1934-z.
25. Moriconi C, Palmieri V, Di Santo R, Tornillo G, Papi M, Pilkington G, et al. INSIDIA: A FIJI Macro Delivering High-Throughput and High-Content Spheroid Invasion Analysis. Biotechnology Journal 2017;12(10):1700140. https://onlinelibrary.wiley.com/doi/abs/10.1002/biot.201700140.
26. Boutin ME, Voss TC, Titus SA, Cruz-Gutierrez K, Michael S, Ferrer M. A high-throughput imaging and nuclear segmentation analysis protocol for cleared 3D culture models. Sci Rep 2018;8(1):11135. https://www.nature.com/articles/s41598-018-29169-0.
27. Chen W, Wong C, Vosburgh E, Levine AJ, Foran DJ, Xu EY. High-throughput Image Analysis of Tumor Spheroids: A User-friendly Software Application to Measure the Size of Spheroids Automatically and Accurately. J Vis Exp 2014;(89):51639. https://www.ncbi.nlm.nih.gov/pmc/articles/PMC4212916/.
28. Piccinini F. AnaSP: A software suite for automatic image analysis of multicellular spheroids. Computer Methods and Programs in Biomedicine 2015;119(1):43–52. https://www.sciencedirect.com/science/article/pii/S0169260715000309.
29. Piccinini F, Peirsman A, Stellato M, Pyun JC, Tumedei MM, Tazzari M, et al. Deep learning-based tool for morphotypic analysis of 3d multicellular spheroids. J Mech Med Biol 2023;23(6):2340034. https://www.worldscientific.com/doi/abs/10.1142/S0219519423400341.
30. Hou Y, Konen J, Brat DJ, Marcus AI, Cooper LAD. TASI: A software tool for spatial-temporal quantification of tumor spheroid dynamics. Sci Rep 2018;8(1):7248. https://www.nature.com/articles/s41598-018-25337-4.
31. Lacalle D, Castro-Abril HA, Randelovic T, Domínguez C, Heras J, Mata E, et al. SpheroidJ: An Open-Source Set of Tools for Spheroid Segmentation. Comput Methods Programs Biomed 2021;200:105837. https://www.sciencedirect.com/science/article/pii/S0169260720316709.
32. Perini G, Rosa E, Friggeri G, Di Pietro L, Barba M, Parolini O, et al. INSIDIA 2.0 High-Throughput Analysis of 3D Cancer Models: Multiparametric Quantification of Graphene Quantum Dots Photothermal Therapy for Glioblastoma and Pancreatic Cancer. International Journal of Molecular Sciences 2022;23(6):3217. https://www.mdpi.com/1422-0067/23/6/3217.
33. Jadon S. A survey of loss functions for semantic segmentation. In: 2020 IEEE Conference on Computational Intelligence in Bioinformatics and Computational Biology (CIBCB); 2020. p. 1–7.
34. Yen JC, Chang FJ, Chang S. A new criterion for automatic multilevel thresholding. IEEE Transactions on Image Processing 1995;4(3):370–378. https://ieeexplore.ieee.org/document/366472.
35. Otsu N. A Threshold Selection Method from Gray-Level Histograms. IEEE Transactions on Systems Man and Cybernetics 1979;9(1):62–66. https://ieeexplore.ieee.org/document/4310076.
36. Roerdink JBTM, Meijster A. The Watershed Transform: Definitions, Algorithms and Parallelization Strategies. Fundamenta Informaticae 2000;41(1):187–228. https://content.iospress.com/articles/fundamenta-informaticae/fi41-1-2-07.
37. Duda RO, Hart PE. Use of the Hough transformation to detect lines and curves in pictures. Commun ACM 1972;15(1):11–15. https://dl.acm.org/doi/10.1145/361237.361242.
38. Caselles V, Kimmel R, Sapiro G. Geodesic Active Contours. International Journal of Computer Vision 1997;22(1):61–79. https://doi.org/10.1023/A:1007979827043.
39. Cisneros Castillo LR, Oancea AD, Stüllein C, Régnier-Vigouroux A. A Novel Computer-Assisted Approach to evaluate Multicellular Tumor Spheroid Invasion Assay. Sci Rep 2016;6(1):35099. https://www.nature.com/articles/srep35099.
40. Vinci M, Gowan S, Boxall F, Patterson L, Zimmermann M, Court W, et al. Advances in establishment and analysis of three-dimensional tumor spheroid-based functional assays for target validation and drug evaluation. BMC Biology 2012;10(1):29. https://doi.org/10.1186/1741-7007-10-29.
41. Cornelissen F, Cik M, Gustin E. Phaedra, a Protocol-Driven System for Analysis and Validation of High-Content Imaging and Flow Cytometry. SLAS Discovery 2012;17(4):496–508. https://www.sciencedirect.com/science/article/pii/S2472555222076341.

42. Sadanandan SK, Karlsson J, Wählby C. Spheroid Segmentation Using Multiscale Deep Adversarial Networks. In: 2017 IEEE International Conference on Computer Vision Workshops (ICCVW); 2017. p. 36–41. https://ieeexplore.ieee.org/document/8265222.
43. Celli JP, Rizvi I, Blanden AR, Massodi I, Glidden MD, Pogue BW, et al. An imaging-based platform for high-content, quantitative evaluation of therapeutic response in 3D tumour models. Sci Rep 2014;4(1):3751. https://www.nature.com/articles/srep03751.
44. Piccinini F, Tesei A, Zanoni M, Bevilacqua A. ReViMS: Software tool for estimating the volumes of 3-D multicellular spheroids imaged using a light sheet fluorescence microscope. BioTechniques 2017;63(5):227–229. https://www.future-science.com/doi/10.2144/000114609.
45. Ngo TKN, Yang SJ, Mao BH, Nguyen TKM, Ng QD, Kuo YL, et al. A deep learning-based pipeline for analyzing the influences of interfacial mechanochemical microenvironments on spheroid invasion using differential interference contrast microscopic images. Materials Today Bio 2023;23:100820. https://www.sciencedirect.com/science/article/pii/S2590006423002806.
46. Chen Z, Ma N, Sun X, Li Q, Zeng Y, Chen F, et al. Automated evaluation of tumor spheroid behavior in 3D culture using deep learning-based recognition. Biomaterials 2021;272:120770. https://www.sciencedirect.com/science/article/pii/S0142961221001265.
47. Zhuang M, Chen Z, Wang H, Tang H, He J, Qin B, et al. AnatomySketch: An Extensible Open-Source Software Platform for Medical Image Analysis Algorithm Development. J Digit Imaging 2022;35(6):1623–1633. https://doi.org/10.1007/s10278-022-00660-5.
48. Akshay A, Katoch M, Abedi M, Shekarchizadeh N, Besic M, Burkhard FC, et al. SpheroScan: a user-friendly deep learning tool for spheroid image analysis. GigaScience 2023;12:giad082. https://doi.org/10.1093/gigascience/giad082.
49. García-Domínguez M, Domínguez C, Heras J, Mata E, Pascual V. Deep style transfer to deal with the domain shift problem on spheroid segmentation. Neurocomputing 2024;569:127105. https://www.sciencedirect.com/science/article/pii/S0925231223012286.
50. Howard J, Gugger S. Fastai: A Layered API for Deep Learning. Information 2020 Feb;11(2):108. https://www.mdpi.com/2078-2489/11/2/108.
51. Castillo DL, SemTorch; 2020. https://pypi.org/project/SemTorch/#description.
52. Long J, Shelhamer E, Darrell T. Fully convolutional networks for semantic segmentation. IEEE Computer Society; 2015. p. 3431–3440. https://www.computer.org/csdl/proceedings-article/cvpr/2015/07298965/12OmNy49sME.
53. Ronneberger O, Fischer P, Brox T. U-Net: Convolutional Networks for Biomedical Image Segmentation. In: Navab N, Hornegger J, Wells WM, Frangi AF, editors. Medical Image Computing and Computer-Assisted Intervention – MICCAI 2015 Lecture Notes in Computer Science, Springer International Publishing; 2015. p. 234–241.
54. Wang J, Sun K, Cheng T, Jiang B, Deng C, Zhao Y, et al. Deep High-Resolution Representation Learning for Visual Recognition. IEEE Trans Pattern Anal Mach Intell 2021;43(10):3349–3364.
55. Smith LN, A disciplined approach to neural network hyper-parameters: Part 1 – learning rate, batch size, momentum, and weight decay. arXiv; 2018. http://arxiv.org/abs/1803.09820.
56. Suzuki S, be K. Topological structural analysis of digitized binary images by border following. Computer Vision, Graphics, and Image Processing 1985 Apr;30(1):32–46. https://www.sciencedirect.com/science/article/pii/0734189X85900167.
57. Deng J, Dong W, Socher R, Li LJ, Li K, Fei-Fei L. Imagenet: A large-scale hierarchical image database. In: 2009 IEEE conference on computer vision and pattern recognition Ieee; 2009. p. 248–255.
58. Kingma DP, Ba J, Adam: A Method for Stochastic Optimization. arXiv; 2017. http://arxiv.org/abs/1412.6980, arXiv:1412.6980 [cs].
59. Liu L, Jiang H, He P, Chen W, Liu X, Gao J, et al., On the Variance of the Adaptive Learning Rate and Beyond. arXiv; 2021. http://arxiv.org/abs/1908.03265, arXiv:1908.03265 [cs, stat].
60. Zhang M, Lucas J, Ba J, Hinton GE. Lookahead Optimizer: k steps forward, 1 step back. In: Advances in Neural Information Processing Systems, vol. 32 Curran Associates, Inc.; 2019. https://proceedings.neurips.cc/paper/2019/hash/90fd4f88f588ae64038134f1eeaa023f-Abstract.html.
61. Buslaev A, Iglovikov VI, Khvedchenya E, Parinov A, Druzhinin M, Kalinin AA. Albumentations: Fast and Flexible Image Augmentations. Information 2020;11(2):125. https://www.mdpi.com/2078-2489/11/2/125.
62. Isensee F, Jaeger PF, Kohl SAA, Petersen J, Maier-Hein KH. nnU-Net: a self-configuring method for deep learning-based biomedical image segmentation. Nature Methods 2021;18(2):203–211. https://www.nature.com/articles/s41592-020-01008-z.
63. developers OR, ONNX Runtime; 2021. Version: 1.11.1. https://onnxruntime.ai/.

A Supplemental figures and tables (SM)

(a) Untreated spheroid w/o cell debris **(b)** Treated spheroid with cell debris

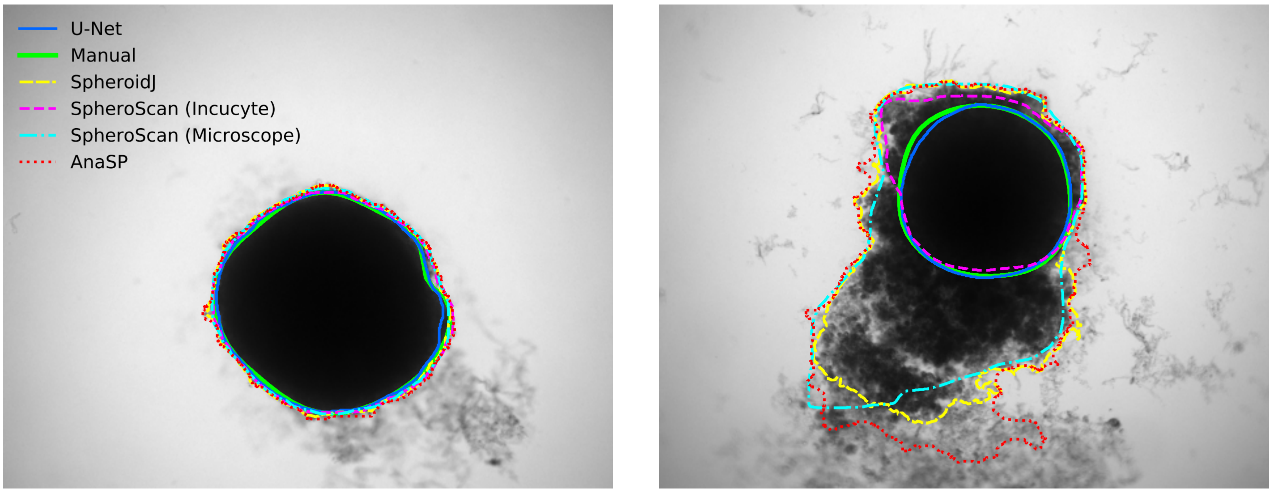

Figure SM.1. Example images illustrating the current challenge of segmenting tumor spheroids: (a) While previously developed deep-learning models generalize excellently to new image data with well-distinguished, unobscured spheroids, typical for untreated cultures, (b) these models fail for cases of detached/relapsing spheroids after radiotherapy (one of the most common cancer treatments) due to debris and dead cells obscuring the spheroid. Shown are representative images of FaDu spheroids (a) without treatment and (b) several days after radiotherapy. Different segmentations are indicated by their outer contours. In particular, the manually set ground truth (green solid line) is compared to the four most recent deep-learning models provided by SpheroidJ [31] (HRNet-Seg with HRNet W30 backbone, yellow close-dashed line), SpheroScan [48] (Region-based Convolutional Neural Network trained on images from IncuCyte Live-Cell Analysis System (purple dashed line) and ordinary microscope (torquoise dashed line) with recommended threshold 0.8), and AnaSP [29] (ResNet18, red dotted line). Only the U-Net presented in this manuscript (blue solid line) performs sufficiently in both scenarios. Size of each image corresponds to 2650 μm $\times$ 2100 μm with a resolution of 1300 $\times$ 1030 pixel.

Table SM.1. Statistical results reflecting the current challenge of segmenting tumor spheroids, corroborating Fig. SM.1: While previously developed deep-learning models generalize excellently to new image data with well-visible, unobscured spheroids (top line), these models fail for typical cases of detached/relapsing spheroids with debris of dead cells (bottom lines). Intersection over Union (IoU or Jaccard index) is reported (average (median) ± standard deviation) for each of the four most recent deep-learning models (provided by SpheroidJ [31], SpheroScan [48] (with recommended threshold 0.8), and AnaSP [29]) and each of our data sets. Only the U-Net presented in this manuscript performs sufficiently in both scenarios. Bold values highlight the optimum for each dataset.

Data set	#Images	Intersection over Union (IoU = 1-JCD)				
		U-Net	SpheroidJ [31]	SpheroScan (Incu) [48]	SpheroScan (Micro) [48]	AnaSP [29]
Spheroids without cell debris	200	**0.97(0.97) ± 0.01**	0.89(0.90) ± 0.05	0.90(0.94) ± 0.16	0.72(0.92) ± 0.38	0.81(0.87) ± 0.22
Training	883	**0.94(0.95) ± 0.05**	0.51(0.53) ± 0.27	0.68(0.86) ± 0.34	0.48(0.59) ± 0.37	0.46(0.44) ± 0.28
Validation	108	**0.95(0.96) ± 0.04**	0.31(0.29) ± 0.19	0.59(0.80) ± 0.39	0.36(0.26) ± 0.32	0.28(0.25) ± 0.19
Test	104	**0.94(0.96) ± 0.06**	0.41(0.38) ± 0.27	0.66(0.88) ± 0.36	0.48(0.50) ± 0.38	0.35(0.33) ± 0.24

Table SM.2. Further validation of the trained U-Net on wide variety of published test data sets from previous deep-learning models. While the trained U-Net performs well on on roughly half of the data sets or 38% of the images (with average IoU above 0.8), sometimes surpassing the original model corresponding to the data set, two types of images turn out problematic: (i) images with ambiguous ground truth (20% of images) for which the U-Net may actually segment reasonably, see main text for detailed discussion, and (ii) images on which the spheroids appears semi-transparent (42% of images), with individual cells being visible throughout the spheroid, potentially due to its small size or the chosen microscopy method. However, classical segmentation techniques work sufficiently well for both types of images (i) and (ii), making the use of deep-learning approaches in these cases unnecessary. ~~To demonstrate this~~For demonstration, the last two columns report the performance of the classical approach from the original publication and simple Otsu thresholding (sometimes after some image erosion for (ii)) performed by us for images of types (i) and (ii). Note that the notation for data sets from Refs. [22, 31] stands for brightfield/fluorescence microscopy (B/F), Nikon Eclipse/Leica DMi8/Olympus microscope (N,L,O), 2x/5x/10x magnification (2/5/10), and suspension/collagen culture (S/C). Bold values highlight the optimum for each dataset.

Data set	#Images	Comments	Intersection over Union (IoU = 1-JCD)			
			deep-learning model		classical segmentation	
			Original	U-Net	Original	Otsu thresholding
BO10S [22]	66		0.92 ± 0.03 [31]	**0.97(0.98) ± 0.05**	0.94 ± 0.03 [31]	-
BN10S [31]	21		**0.97 ± 0.01**	0.89(0.89) ± 0.01	0.95 ± 0.01	0.84(0.85) ± 0.13
	84	ambiguous ground truth		0.61(0.62) ± 0.15		
BN2S [31]	154	semi-transparent	**0.96 ± 0.01**	0.30(0.27) ± 0.22	0.94 ± 0.02	0.87(0.95) ± 0.27
BL5S [31]	50	semi-transparent	0.75 ± 0.25	0.37(0.13) ± 0.39	0.64 ± 0.30	**0.85(0.93) ± 0.20**
FN2S [31]	30		0.78 ± 0.20	0.91(0.94) ± 0.08	**0.82 ± 0.17**	-
	4	semi-transparent		0		0.83(0.83) ± 0.02
FL5C[31]	4		0.71 ± 0.30	0.92(0.91) ± 0.01	0.67 ± 0.17	-
	15	ambiguous ground truth		0.45(0.45) ± 0.16		**0.80(0.85) ± 0.11**
FL5S [31]	50		0.70 ± 0.26	**0.91(0.91) ± 0.05**	0.89 ± 0.07	-
AnaSP [29]	16		~ 0.92	0.97(0.98) ± 0.02		-
	2	semi-transparent		0.01(0.01) ± 0.01		~~0.97(0.97) ± 0.00~~ 0.97(0.97) ± 0.001

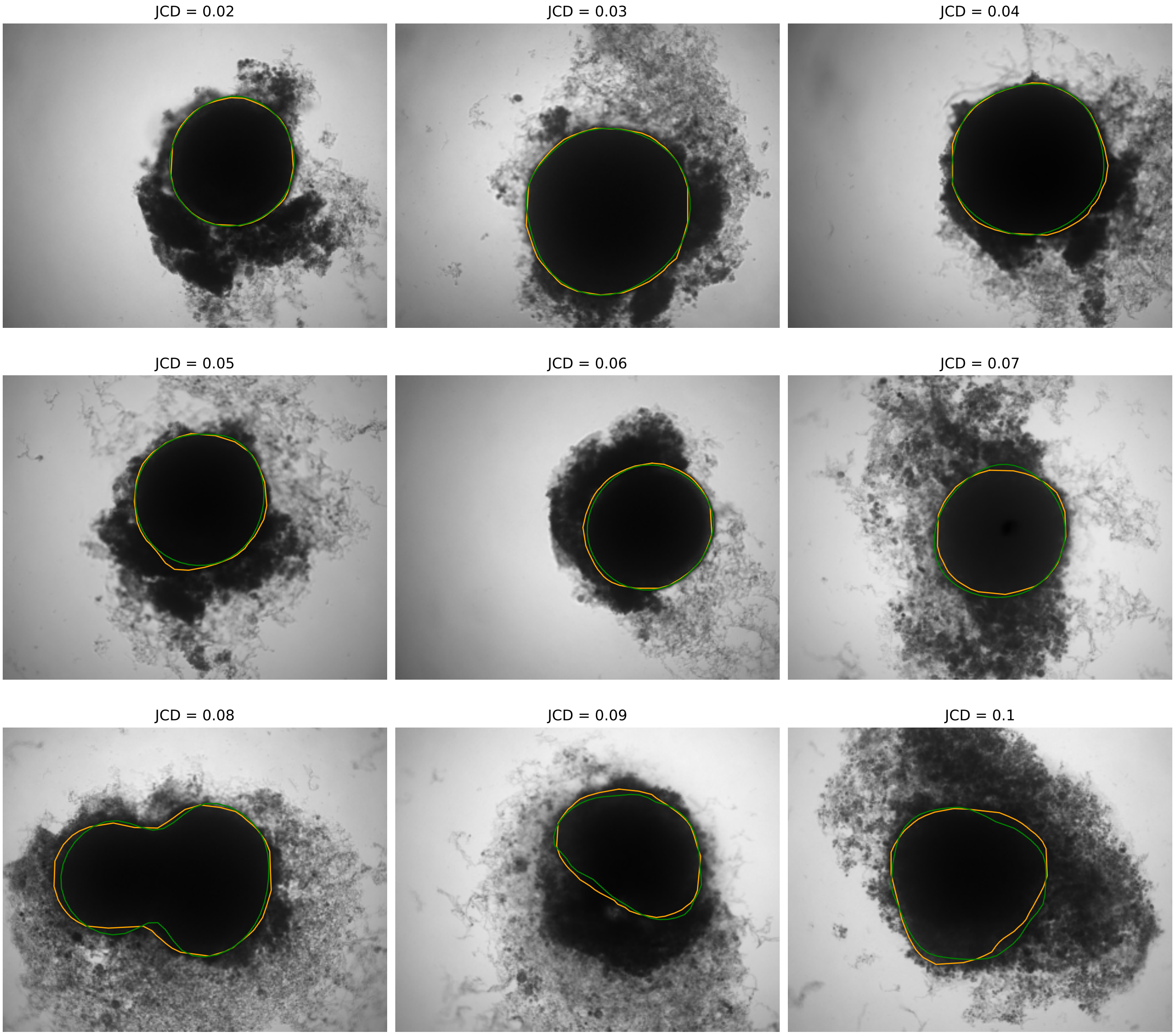

Figure SM.2. Selection of representative images as optical reference for JCD$\leq$ 0.1 with automatic segmentation from the optical U-Net (orange) and manual segmentation from biological expert H2 (green). From the tested images, 52% fall into this range of JCD$\leq$ 0.1 and 67% when only spheroids beyond the standard size d_T > 400 µm are considered. Size of each image corresponds to 2650 µm × 2100 µm. Note that the U-Net is trained on an independent manual segmentations from another biological expert H1. Images are selected from the extended validation data set (Fig. 4), in particular, from the subset of larger, better discriminated spheroids at intermediate levels of debris for illustration. Note that in the example for JCD = 0.08, the two attached spheroids are correctly segmented, although the training data set does not contain such cases.

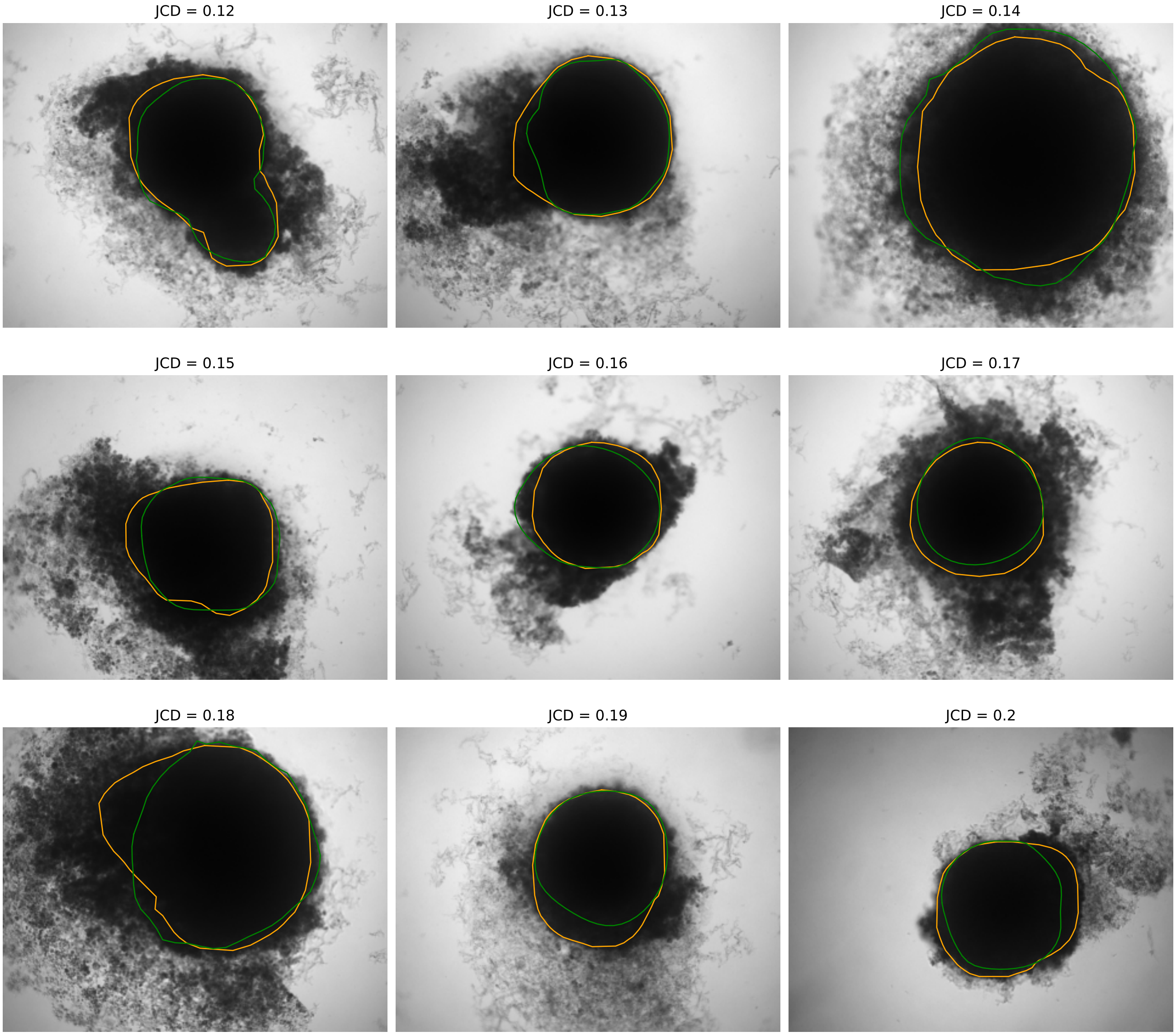

Figure SM.3. Selection of representative images as optical reference for 0.1 < JCD $\leq$ 0.2 with automatic segmentation from the optical U-Net (orange) and manual segmentation from biological expert H2 (green), analogous to Fig. SM.2. From the tested images, 22% fall into the range 0.1 < JCD $\leq$ 0.2 and 23% when only spheroids beyond the standard size d_T > 400 μm are considered. Note that in the example of JCD = 0.12, the two attached spheroids are correctly segmented, although the training data set does not contain such cases.

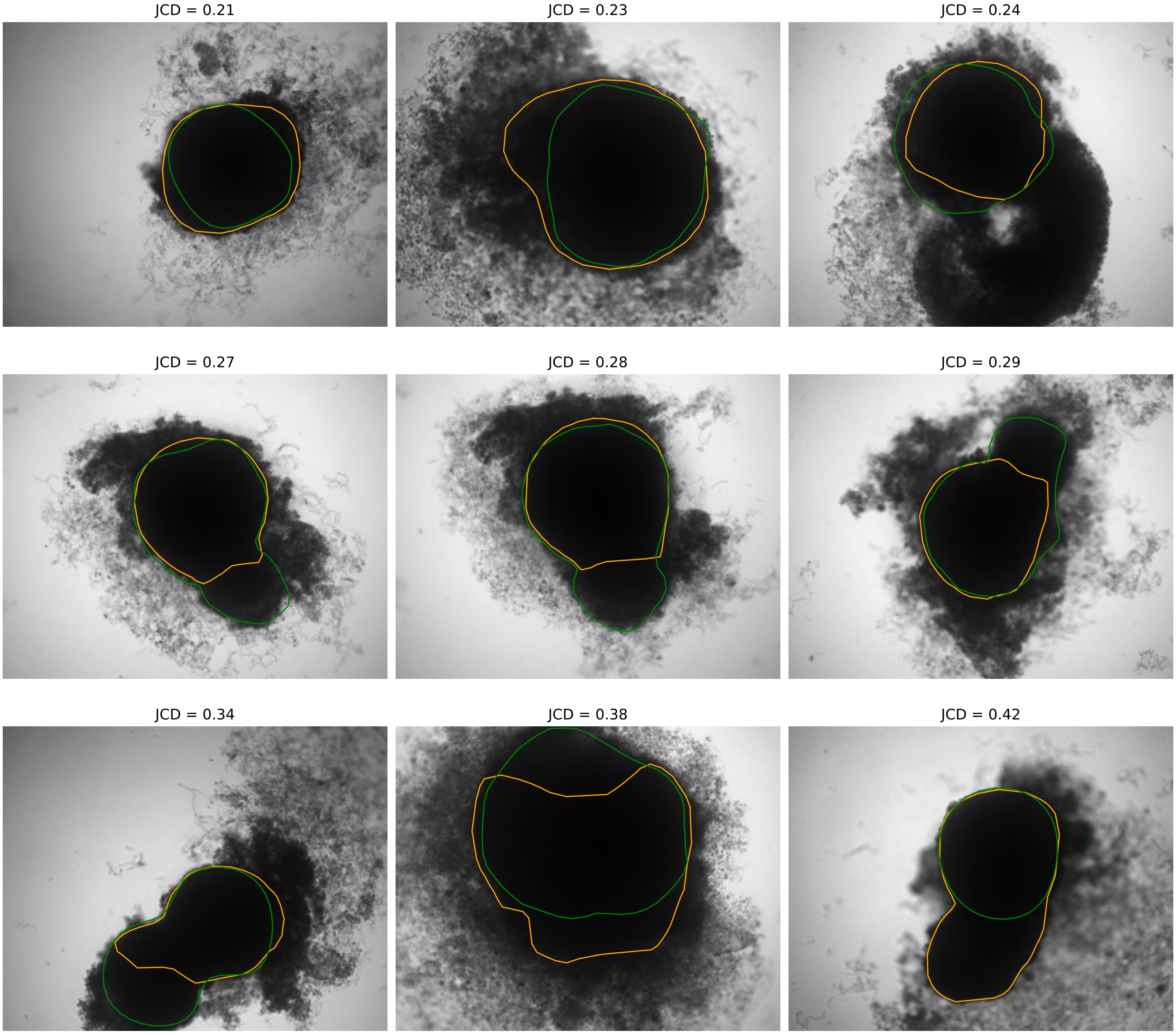

Figure SM.4. Selection of representative images as optical reference for larger deviations JCD > 0.2 of the automatic segmentation from the optical U-Net (orange) and manual segmentation from biological expert H2 (green), analogous to Fig. SM.2. From the tested images, 26% fall into the range JCD > 0.2, but merely 10% when only spheroids beyond the standard size $d_T > 400$ µm are considered. Note that deviations at larger spheroids are often due to cases of double-spheroids, e.g., JCD = 0.27, 0.28, 0.29, 0.34, 0.42, which are inconsistently recognized as either one or two spheroids even by the human.

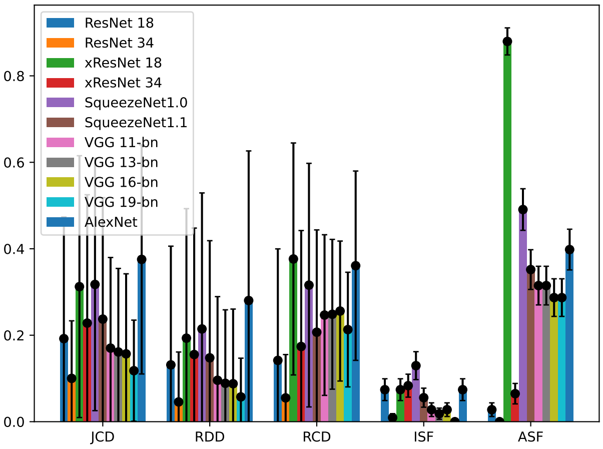

Backbone	JCD	RDD	RCD	ISF	ASF
ResNet 18	0.192	0.131	0.142	0.074	0.028
±	0.282	0.275	0.258	0.025	0.016
ResNet 34	**0.100**	**0.046**	**0.055**	0.009	**0.000**
±	0.133	0.116	0.100	0.009	0.000
xResNet 18	0.312	0.193	0.376	0.074	0.880
±	0.303	0.300	0.268	0.025	0.031
xResNet 34	0.228	0.155	0.174	0.083	0.065
±	0.297	0.292	0.268	0.027	0.024
SqueezeNet1.0	0.317	0.215	0.316	0.130	0.491
±	0.292	0.315	0.282	0.032	0.048
SqueezeNet1.1	0.237	0.148	0.207	0.056	0.352
±	0.281	0.271	0.237	0.022	0.046
VGG 11-bn	0.170	0.096	0.247	0.028	0.315
±	0.210	0.193	0.186	0.016	0.045
VGG 13-bn	0.161	0.089	0.248	0.019	0.315
±	0.193	0.170	0.173	0.013	0.045
VGG 16-bn	0.157	0.088	0.256	0.028	0.287
±	0.185	0.173	0.162	0.016	0.044
VGG 19-bn	0.118	0.057	0.213	**0.000**	0.287
±	0.117	0.090	0.133	0.000	0.044
AlexNet	0.375	0.280	0.361	0.074	0.398
±	0.265	0.346	0.219	0.025	0.047

Figure SM.5. Values of evaluation metrics for different backbones of the U-Net. Bold values highlight the optimum in each column. The other hyperparameters are fixed at Optimizer: Adam, Loss: Cross-Entropy, Resize factor: 1/2, Transfer learning: Yes, Data augmentation: No. Overall the ResNet 34 achieved the best results and is picked as the backbone for the U-Net.

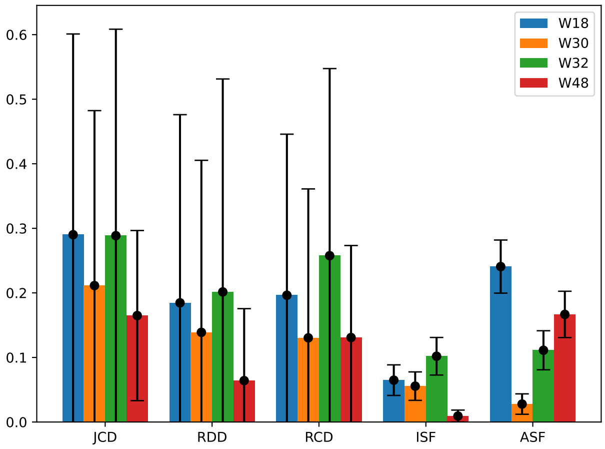

Backbone	JCD	RDD	RCD	ISF	ASF
W18	0.290	0.185	0.196	0.065	0.241
±	0.311	0.291	0.249	0.024	0.041
W30	0.211	0.139	**0.130**	0.056	**0.028**
±	0.271	0.266	0.231	0.022	0.016
W32	0.289	0.201	0.258	0.102	0.111
±	0.320	0.330	0.290	0.029	0.030
W48	**0.165**	**0.064**	0.131	**0.009**	0.167
±	0.132	0.111	0.143	0.009	0.036

Figure SM.6. Values of evaluation metrics for different backbones of the HRNet. Bold values highlight the optimum in each column. The other hyperparameters are fixed at Optimizer: Adam, Loss: Cross-Entropy, Resize factor: 1/2, Transfer learning: Yes, Data augmentation: No. Overall the W48 achieved the best results and is picked as the backbone for the HRNet.

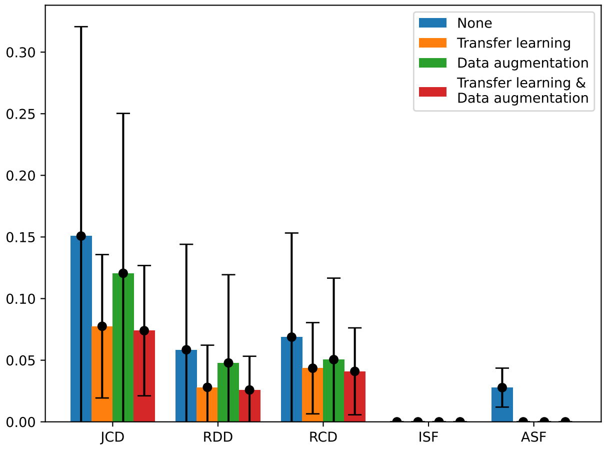

Extension	JCD	RDD	RCD	ISF	ASF
None	0.151	0.058	0.069	**0.000**	0.028
±	0.170	0.086	0.084	0.000	0.016
Transfer learning	0.078	0.028	0.043	**0.000**	**0.000**
±	0.058	0.034	0.037	0.000	0.000
Data augmentation	0.121	0.048	0.050	**0.000**	**0.000**
±	0.130	0.072	0.066	0.000	0.000
Transfer learning &	**0.074**	**0.026**	**0.041**	**0.000**	**0.000**
Data augmentation ±	0.053	0.027	0.035	0.000	0.000

Figure SM.7. Values of evaluation metrics for different extensions of the training data set, which is used by the U-Net. Bold values highlight the optimum in each column. The other hyperparameters are fixed at Backbone: ResNet 34, Optimizer: Adam, Loss: Cross-Entropy, Resize factor: 1/2. The accuracy of the U-Net is highest when transfer learning and data augmentation is used.

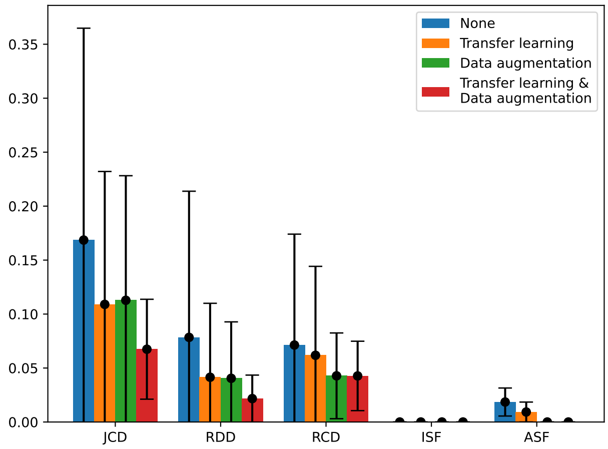

Extension	JCD	RDD	RCD	ISF	ASF
None	0.169	0.078	0.071	**0.000**	0.019
±	0.196	0.135	0.103	0.000	0.013
Transfer learning	0.109	0.041	0.062	**0.000**	0.009
±	0.123	0.069	0.082	0.000	0.009
Data augmentation	0.113	0.040	**0.043**	**0.000**	**0.000**
±	0.115	0.052	0.040	0.000	0.000
Transfer learning &	**0.067**	**0.022**	**0.043**	**0.000**	**0.000**
Data augmentation ±	0.046	0.022	0.032	0.000	0.000

Figure SM.8. Values of evaluation metrics for different extensions of the training data set, which is used by the HRNet. Bold values highlight the optimum in each column. The other hyperparameters are fixed at Backbone: W48, Optimizer: Adam, Loss: Cross-Entropy, Resize factor: 1/2. The accuracy of the HRNet is highest when transfer learning and data augmentation is used.

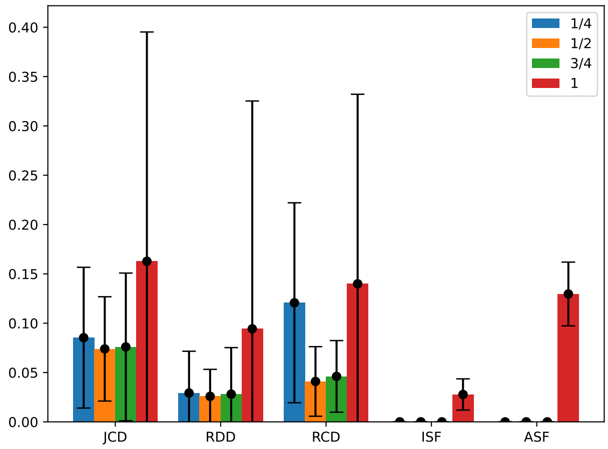

Resize factor	JCD	RDD	RCD	ISF	ASF
1/4	0.085	0.029	0.121	**0.000**	**0.000**
±	0.071	0.042	0.101	0.000	0.000
1/2	**0.074**	**0.026**	**0.041**	**0.000**	**0.000**
±	0.053	0.027	0.035	0.000	0.000
3/4	0.076	0.028	0.046	**0.000**	**0.000**
±	0.075	0.047	0.036	0.000	0.000
1	0.163	0.094	0.140	0.028	0.130
±	0.232	0.231	0.192	0.016	0.032

Figure SM.9. Values of evaluation metrics for different image sizes. Bold values highlight the optimum in each column. The other hyperparameters are fixed at Backbone: ResNet 34, Optimizer: Adam, Loss: Cross-Entropy, Transfer learning: Yes, Data augmentation: Yes. The U-Net can achieve the highest accuracy when the original image size is reduced by half.

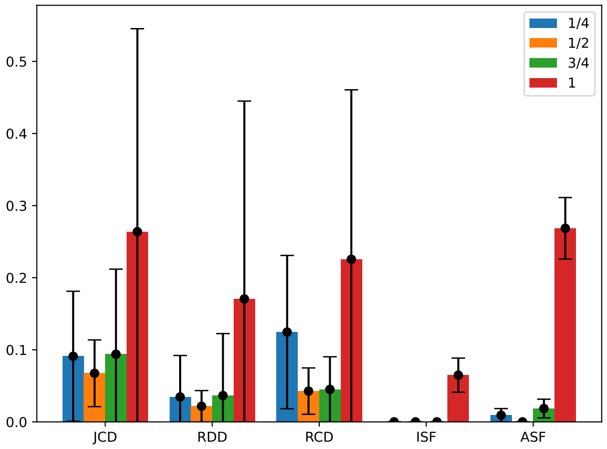

Resize factor	JCD	RDD	RCD	ISF	ASF
1/4	0.091	0.035	0.125	**0.000**	0.009
±	0.090	0.058	0.106	0.000	0.009
1/2	**0.067**	**0.022**	**0.043**	**0.000**	**0.000**
±	0.046	0.022	0.032	0.000	0.000
3/4	0.094	0.037	0.045	**0.000**	0.019
±	0.118	0.086	0.045	0.000	0.013
1	0.264	0.171	0.226	0.065	0.269
±	0.282	0.274	0.235	0.024	0.043

Figure SM.10. Values of evaluation metrics for different image sizes. Bold values highlight the optimum in each column. The other hyperparameters are fixed at Backbone: W48, Optimizer: Adam, Loss: Cross-Entropy, Transfer learning: Yes, Data augmentation: Yes. The HRNet can achieve the highest accuracy when the original image size is reduced by half.

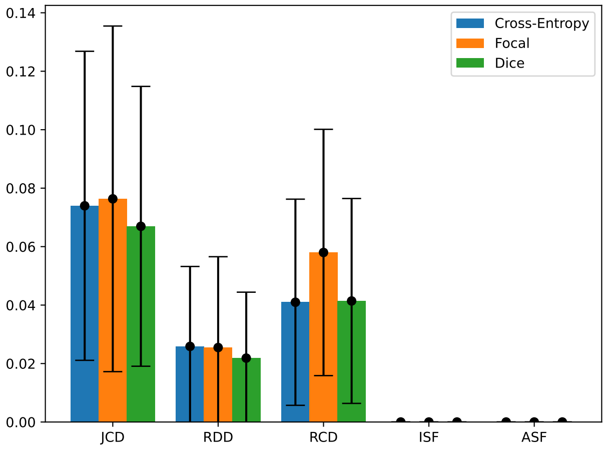

Loss function	JCD	RDD	RCD	ISF	ASF
Cross-Entropy	0.074	0.026	0.041	**0.000**	**0.000**
±	0.053	0.027	0.035	0.000	0.000
Focal	0.076	0.025	0.058	**0.000**	**0.000**
±	0.059	0.031	0.042	0.000	0.000
Dice	**0.067**	**0.022**	**0.041**	**0.000**	**0.000**
±	0.048	0.023	0.035	0.000	0.000

Figure SM.11. Values of evaluation metrics for different loss functions, used by the U-Net. Bold values highlight the optimum in each column. The other hyperparameters are fixed at Backbone: ResNet 34, Optimizer: Adam, Resize factor: 1/2, Transfer learning: Yes, Data augmentation: Yes. The U-Net can achieve the highest accuracy when the Dice loss is used.

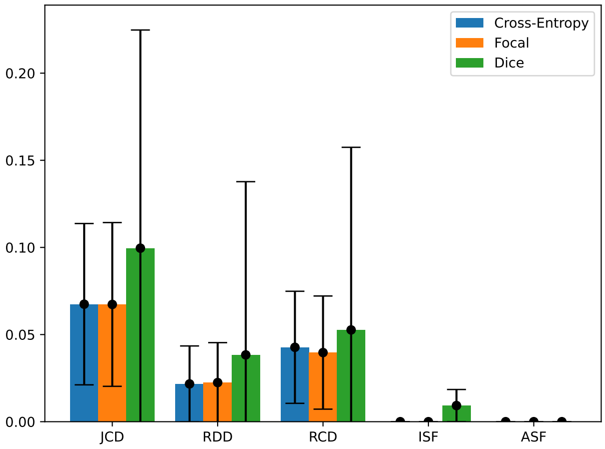

Loss function	JCD	RDD	RCD	ISF	ASF
Cross-Entropy	**0.067**	**0.022**	0.043	**0.000**	**0.000**
±	0.046	0.022	0.032	0.000	0.000
Focal	**0.067**	**0.022**	**0.040**	**0.000**	**0.000**
±	0.047	0.023	0.032	0.000	0.000
Dice	0.100	0.038	0.053	0.009	**0.000**
±	0.125	0.099	0.105	0.009	0.000

Figure SM.12. Values of evaluation metrics for different loss functions, used by the HRNet. Bold values highlight the optimum in each column. The other hyperparameters are fixed at Backbone: W48, Optimizer: Adam, Resize factor: 1/2, Transfer learning: Yes, Data augmentation: Yes. The HRNet can achieve the highest accuracy, if the Cross-Entropy or the Focal loss is used.

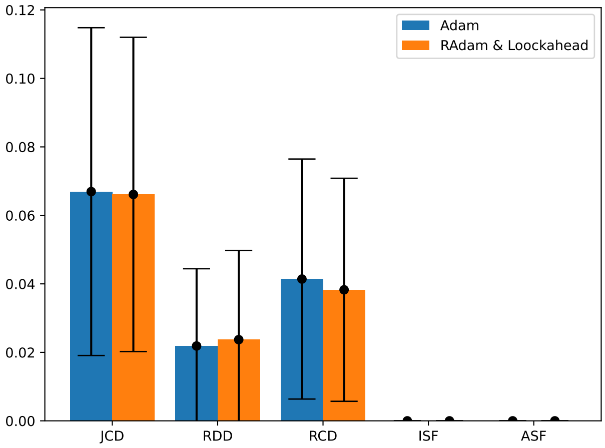

Optimizer function	JCD	RDD	RCD	ISF	ASF
Adam	0.067	**0.022**	0.041	**0.000**	**0.000**
±	0.048	0.023	0.035	0.000	0.000
RAdam & Lookahead	**0.066**	0.024	**0.038**	**0.000**	**0.000**
±	0.046	0.026	0.033	0.000	0.000

Figure SM.13. Values of evaluation metrics for different optimizers, used by the U-Net. Bold values highlight the optimum in each column. The other hyperparameters are fixed at Backbone: ResNet 34, Loss: Dice, Resize factor: 1/2, Transfer learning: Yes, Data augmentation: Yes. The U-Net can achieve the highest accuracy when the optimization is done by RAdam combined with Lookahead.

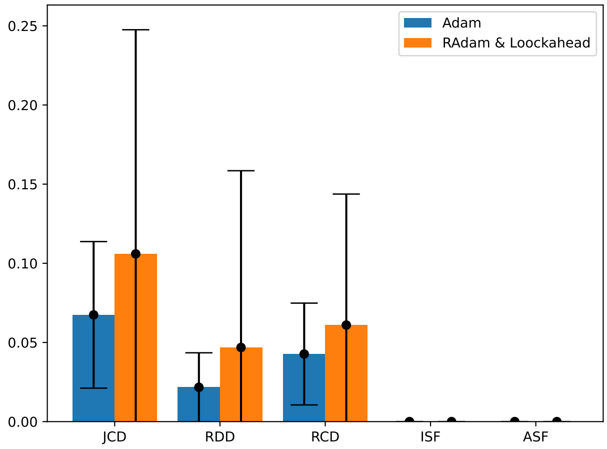

Optimizer function	JCD	RDD	RCD	ISF	ASF
Adam	**0.067**	**0.022**	**0.043**	**0.000**	**0.000**
±	0.046	0.022	0.032	0.000	0.000
RAdam & Lookahead	0.106	0.047	0.061	**0.000**	**0.000**
±	0.142	0.112	0.083	0.000	0.000

Figure SM.14. Values of evaluation metrics for different optimizers, used by the HRNet. Bold values highlight the optimum in each column. The other hyperparameters are fixed at Backbone: W48, Loss: Cross-Entropy, Resize factor: 1/2, Transfer learning: Yes, Data augmentation: Yes. The HRNet can achieve the highest accuracy when the optimization is done by Adam.

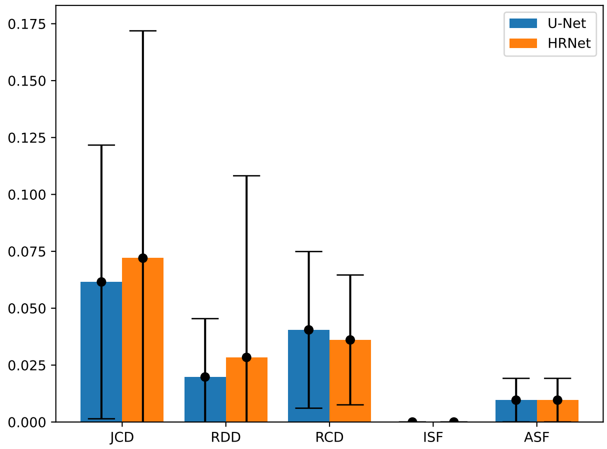

Model	JCD	RDD	RCD	ISF	ASF
U-Net	**0.062**	**0.020**	0.040	**0.000**	**0.010**
±	0.060	0.026	0.034	0.000	0.010
HRNet	0.072	0.028	**0.036**	**0.000**	**0.010**
±	0.100	0.080	0.029	0.000	0.010

Figure SM.15. Evaluation of the segmentation with the optimized U-Net and HRNet models on the test data set shows higher accuracy of the U-Net. The optimal hyperparameter configuration for the U-Net is Backbone: ResNet 34, Optimizer: RAdam & Lookahead, Loss: Dice, Resize factor: 1/2, Transfer learning: Yes, Data augmentation: Yes. The final hyperparameter configuration for the HRNet is Backbone: W48, Optimizer: Adam, Loss: Cross-Entropy, Resize factor: 1/2, Transfer learning: Yes, Data augmentation: Yes.

(a) Image with segmentations **(b)** Segmentations with mismatched (gray) areas **(c)** Corresponding circle with rim of width $\triangle r$

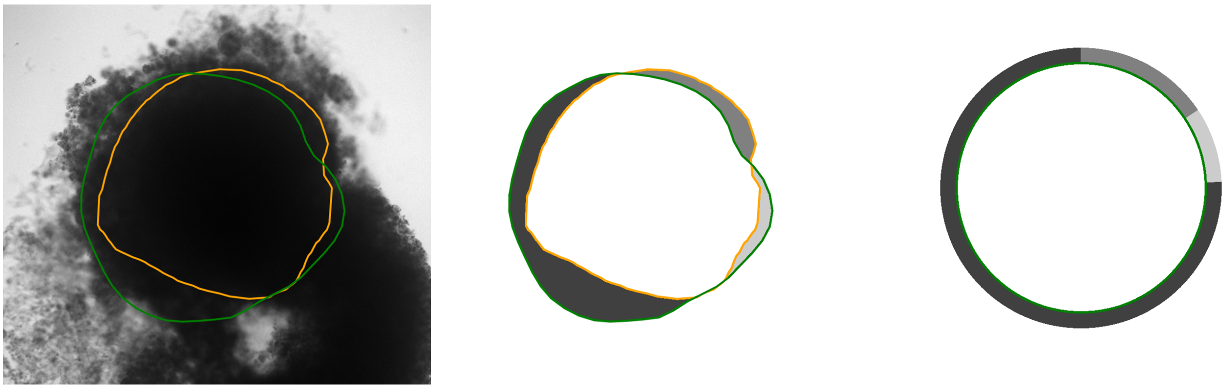

Figure SM.16. Illustration of the average radial error $\triangle r$ defined in Eq. (3) (example with large JCD > 0.2 for visibility). (a) Zoom of image from Fig. SM.4 with JCD = 0.24 with corresponding automatic (orange line containing predicted area P) and manual segmentation (green line containing target area T). (b) Segmentations from (a) with mismatched areas shown in gray (missing area $T \setminus P$ in bright and dark gray, additional area $P \setminus T$ in intermediate gray). (c) Circle (green line) and added rim (gray areas) with areas corresponding to (b): Area within green circle is equal to area within manual segmentation in (b) and gray areas within the adjacent rim are equal to corresponding mismatched areas in (b). Then the radial thickness of the rim is equal to the average radial error $\triangle r$ defined in Eq. (3).

(a) RDD over spheroid diameter

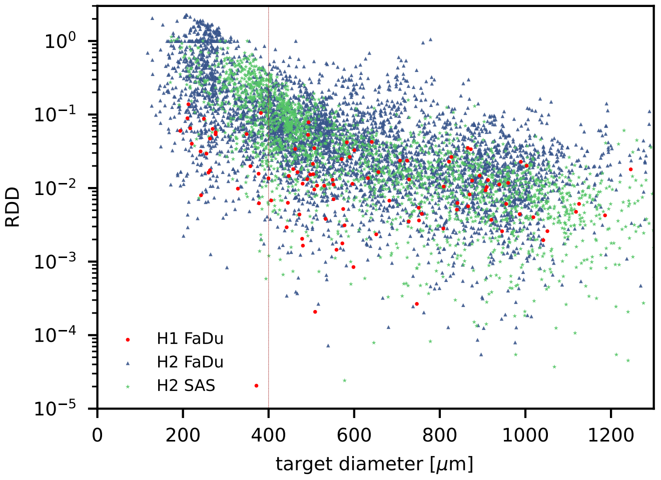

(b) Comparison diameters from manual and automatic segmentation

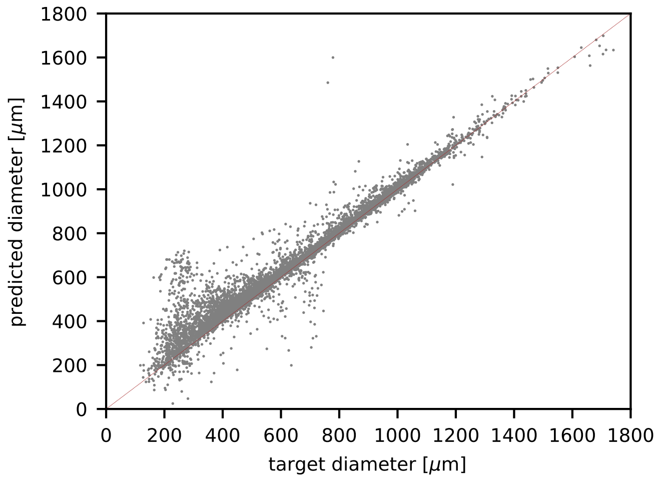

Figure SM.17. Validation based on diameter of automatic segmentation with the optimized U-Net on larger, independent data sets, analogous to Fig. 4. (a) RDD shows that majority of deviations are below 10% and higher deviations occur mostly below $d_T < 400$ µm. (b) Direct comparison of diameters (gray points) resulting from automatic segmentation (predicted diameter) and manual segmentation (target diameter) shows high accuracy (red line represents perfect match) with larger deviations mostly at $d_T < 400$ µm.

Reviewer 1-2 remarks and author replies

We thank the referees for their insightful and constructive reviews, which improved the clarity and value of the manuscript considerably. We followed the excellent comments and suggestions of the reviewers and revised our manuscript as detailed in the following.

Reviewer #1:
Dear Editor and Dear Colleagues, I have carefully reviewed the manuscript titled "Image segmentation of treated and untreated tumor spheroids by Fully Convolutional Networks" submitted for consideration in GigaScience. I really liked the article and how the information have been presented. Here are a few suggestions to improve the clarity of this manuscript.
1. In the "Methods" section there is the following sentence: "These metrics are used to optimize the hyperparameters, Section, and data augmentation, Section, of the selected FCN models U-Net and HRNet for maximal accuracy.". I think you may have forgotten to specify the section where the information is described more in depth. It could be useful to re-write it for clarity

Indeed, the section references were broken. We fixed the issue and throughout the manuscript sections are now correctly referenced by their title.

2. In the "Evaluation metrics" section there is the following sentence: "In the rare case that several spheroids are detected in an image, the largest one is chosen for computation of the JCD." Why don't you calculate all of the spheroids detected? And why do you choose the biggest one?

We updated the paragraph to:

"In the rare case that several spheroids are detected in an image, the largest one is chosen for computation of the JCD. This is in accordance with experimental standard practice for spheroid-based assays, as the largest regrowing spheroid is sufficient to detect relapse after treatment. Note that if there were several spheroids present and of interest, also all of them could be segmented."

We also marked in the code repo, where the case of several relevant spheroids could be incorporated.

3. In the "Hyperparameter optimization" section, you didn't insert the number of the section where the data augmentation is described.

Has been fixed, see reply to point 1.

4. In table SM.2, the standard deviation related to the Otsu thresholding segmentation of the AnaSP line is 0.0. How is that possible?

We only performed additional Otsu thresholding for images of type (i, ambiguous ground truth) and (ii, semi-transparent) to demonstrate that cases where our segmentation fails, are actually cases for which classical segmentation methods are sufficient and no deep-learning approach is required. For the AnaSP sample data only 2/18 images belong to type (ii) and for these two images Otsu thresholding achieves values

$$IoU = (0.9728834983597676,\ 0.970423820294027)$$

which averages to

$$0.9716536593268973\ (0.9716536593268973)\ \pm\ 0.0012298390328703\,28,$$

which rounds to the reported 0.97(0.97) $\pm$ 0.00.
To avoid confusion we added another significant digit to the standard deviation related to the Otsu thresholding segmentation of the AnaSP line and added dashes to lines for which no additional Otsu thresholding was performed. We also added *"for images of type (i) and (ii)"* to the respective passage in the caption of Tab. SM.2.:

"To demonstrate this, the last two columns report the performance of the classical approach from the original publication and simple Otsu thresholding (sometimes after some image erosion for (ii)) performed by us for images of type (i) and (ii)."

5. At the end of the "Discussion" section there is the following sentence: "... which is consistent with previously reported optimizations of deep-learning models [31, Tab. 2] [29, Tab. 3].". I wasn't able to find Tab. 2 and Tab. 3 in the text or in the supplementary materials.

We refer to the tables within the cited references for the convenience of the reader, i.e., [1, Tab. 2] means Tab. 2 in Ref. [1] and [2, Tab. 3] means Tab. 3 in Ref. [2]. To our knowledge this follows APA style guidelines, but we are open for other syntax.

Thank you for considering my feedback, and I am available for further clarification or discussion if needed. Sincerely.

Reviewer #2:
In this paper, the authors thoroughly study two segmentation architectures to analyse images of treated and untreated tumor spheroids. It was a pleasure to read this paper since the authors made an amazing job explaining the methodology and all the steps that they followed. The authors deal with more complex images of spheroids than those usually found in the literature of spheroid segmentation, and their approach obtained good results. Besides, they released both the dataset and code to train and use their models.
The only small issue that I have detected in the manuscript is that references to sections are missing.

Indeed, the section references were broken. We fixed the issue and throughout the manuscript sections are now correctly referenced by their title.

References

[1] D. Lacalle, H. A. Castro-Abril, T. Randelovic, C. Domínguez, J. Heras, E. Mata, G. Mata, Y. MÃ©ndez, V. Pascual, and I. Ochoa, SpheroidJ: An open-source set of tools for spheroid segmentation, Comput. Methods Programs Biomed. **200**, 105837 (2021).

[2] F. Piccinini, A. Peirsman, M. Stellato, J.-C. Pyun, M. M. Tumedei, M. Tazzari, O. De Wever, A. Tesei, G. Martinelli, and G. Castellani, Deep learning-based tool for morphotypic analysis of 3d multicellular spheroids, J. Mech. Med. Biol. **23**, 2340034 (2023).
