# Supplementary material for: Image segmentation of treated and untreated tumor spheroids by fully convolutional networks
Source: Gigascience. 2025 May 7;14:giaf027. doi: 10.1093/gigascience/giaf027 (PMC12056507; doi:10.1093/gigascience/giaf027)
Supplement: giaf027_Authors_Response_To_Reviewer_Comments_Original_Submission [file giaf027_authors_response_to_reviewer_comments_original_submission.pdf]

Author's Response To Reviewer Comments

We detailed our revisions and reply to reviewers in attached review_reply_giga.pdf.
We also attached a compiled version of the revised manuscript with changes highlighted (diff.pdf)
