# Supplementary material for: Image segmentation of treated and untreated tumor spheroids by fully convolutional networks
Source: Gigascience. 2025 May 7;14:giaf027. doi: 10.1093/gigascience/giaf027 (PMC12056507; doi:10.1093/gigascience/giaf027)
Supplement: giaf027_Authors_Response_To_Reviewer_Comments_Revision_1 [file giaf027_authors_response_to_reviewer_comments_revision_1.pdf]

Author's Response To Reviewer Comments

As requested by Qing Lan I update all files associated with the new LaTex version along with a separate pdf of the main text.
