# Supplementary material for: Image segmentation of treated and untreated tumor spheroids by fully convolutional networks
Source: Gigascience. 2025 May 7;14:giaf027. doi: 10.1093/gigascience/giaf027 (PMC12056507; doi:10.1093/gigascience/giaf027)
Supplement: giaf027_Reviewer_1_Report_Original_Submission — Mariachiara Stellato -- 2/3/2025 [file giaf027_reviewer_1_report_original_submission.pdf]

**Reviewer Report**

**Title: Image segmentation of treated and untreated tumor spheroids by Fully Convolutional Networks**

**Version: Original Submission** **Date:** 2/3/2025

**Reviewer name: Mariachiara Stellato**

**Reviewer Comments to Author:**

Title: Review of Manuscript GIGA-D-24-00570Dear Editor and Dear Colleagues,I have carefully reviewed the manuscript titled "Deep Learning-based Automatic Segmentation of Brain Structures on MRI: A Test-Retest Reproducibility Analysis" submitted for consideration in GigaScience. I really liked the article and how the information have been presented. Here are a few suggestions to improve the clarity of this manuscript.1. In the "Methods" section there is the following sentence: "These metrics are used to optimize the hyperparameters, Section, and data augmentation, Section, of the selected FCN models U-Net and HRNet for maximal accuracy.". I think you may have forgotten to specify the section where the information is described more in depth. It could be useful to re-write it for clarity2. In the "Evaluation metrics" section there is the following sentence: "In the rare case that several spheroids are detected in an image, the largest one is chosen for computation of the JCD." Why don't you calculate all of the spheroids detected? And why do you choose the biggest one?3. In the "Hyperparameter optimization" section, you didn't insert the number of the section where the data augmentation is described.4. In table SM.2, the standard deviation related to the Otsu thresholding segmentation of the AnaSP line is 0.0. How is that possible?5. At the end of the "Discussion" section there is the following sentence: "… which is consistent with previously reported optimizations of deep-learning models [31, Tab. 2] [29, Tab. 3].". I wasn't able to find Tab. 2 and Tab. 3 in the text or in the supplementary materials.Thank you for considering my feedback, and I am available for further clarification or discussion if needed.Sincerely.

**Level of Interest**

Please indicate how interesting you found the manuscript: Choose an item.

**Quality of Written English**

Please indicate the quality of language in the manuscript: Choose an item.

**Declaration of Competing Interests**

Please complete a declaration of competing interests, considering the following questions:

- Have you in the past five years received reimbursements, fees, funding, or salary from an organisation that may in any way gain or lose financially from the publication of this manuscript, either now or in the future?

- Do you hold any stocks or shares in an organisation that may in any way gain or lose financially from the publication of this manuscript, either now or in the future?
- Do you hold or are you currently applying for any patents relating to the content of the manuscript?
- Have you received reimbursements, fees, funding, or salary from an organization that holds or has applied for patents relating to the content of the manuscript?
- Do you have any other financial competing interests?
- Do you have any non-financial competing interests in relation to this paper?

If you can answer no to all of the above, write 'I declare that I have no competing interests' below. If your reply is yes to any, please give details below.

I declare that I have no competing interests.

I agree to the open peer review policy of the journal. I understand that my name will be included on my report to the authors and, if the manuscript is accepted for publication, my named report including any attachments I upload will be posted on the website along with the authors' responses. I agree for my report to be made available under an Open Access Creative Commons CC-BY license (http://creativecommons.org/licenses/by/4.0/). I understand that any comments which I do not wish to be included in my named report can be included as confidential comments to the editors, which will not be published.

Choose an item.

To further support our reviewers, we have joined with Publons, where you can gain additional credit to further highlight your hard work (see: https://publons.com/journal/530/gigascience). On publication of this paper, your review will be automatically added to Publons, you can then choose whether or not to claim your Publons credit. I understand this statement.

Yes Choose an item.
